# Atezolizumab–bevacizumab in very elderly with hepatocellular carcinoma: Age alone is not a limiting factor except in ALBI grade 3

**DOI:** 10.1016/j.jhepr.2026.101827

**Published:** 2026-03-17

**Authors:** Chloé Métivier, Claudia Campani, Manon Allaire, Rémy Morello, Sarah Mouri, Eleonore Spitzer, Mohamed Bouattour, Clémence Hollande, Sabrina Sidali, Jean Charles Nault, Nathalie Ganne-Carrié, Pierre Nahon, Giuliana Amaddeo, Hélène Regnault, Paul Vigneron, Jean Marie Péron, Leila Sadek, Cecile Cussac, Marie Lequoy, Violaine Ozenne, Marie-Pierre Galais, Claire Pérignon, Louise Lebedel, Marion Habireche, Apolline Commin, Thông Dao, Charlotte Costentin, Aurore Baron, Isabelle Ollivier Hourmand

**Affiliations:** 1 Department of Hepatogastroenterology, Caen University Hospital, Caen, France; 2 INSERM Unit U1086 ANTICIPE, Caen, France; 3 Functional Genomics of Solid Tumors Team, Cordeliers Research Center, Sorbonne University, Inserm, Paris University, Paris, France; 4 Department of Hepatology, AP-HP Avicenne, Training and Research Unit for Health, Medicine and Human Biology, Paris 13 University, Sorbonne, Paris, Cité Community of Universities and Institutions, Paris, France; 5 Department of Hepatogastroenterology, AP-HP Sorbonne University, Pitié-Salpêtrière Hospital, Paris, France; 6 Biostatistics Department, Caen University Hospital, Caen, France; 7 AP-HP, Beaujon Hospital, Liver Cancer and Innovative Therapy, Clichy, France; 8 INSERM U1149, Inflammation Research Center (CRI), Paris, France; 9 Department of Hepatogastroenterology, AP-HP, Henri Mondor Hospital, Créteil, France; 10 Department of Hepatology, Toulouse University Hospital, Toulouse, France; 11 Department of Hepatology, AP-HP, Saint Antoine Hospital, INSERM UMRS 938, Saint Antoine Research Center, Sorbonne University, Paris, France; 12 Digestive Oncology Department, Francois Baclesse Center, Caen, France; 13 Grenoble Alpes University, Institute of Advanced Biosciences, UGA/Inserm U 1209/CNRS 5309 Research Center, La Tronche, France; 14 Department of Hepatogastroenterology and Digestive Oncology, Grenoble Alpes University Hospital, La Tronche, France; 15 Hepato-biliary Center, Paul Brousse hospital, Inserm uURM, Villejuif, Paris Saclay University, Paris, France

**Keywords:** Hepatocellular carcinoma, Atezolizumab–bevacizumab, Very elderly, ≥75 years

## Abstract

**Background & Aims:**

Evidence on atezolizumab plus bevacizumab (AtezoBev) in very elderly people with hepatocellular carcinoma (HCC) remains rare in the European ancestry population. We compared outcomes in patients aged ≥75 years with younger patients.

**Methods:**

This multicenter retrospective study included patients treated with first-line AtezoBev for advanced HCC. Patients aged ≥75 years were matched 1:1 with those <75 years using propensity scores. Overall survival (OS), progression-free survival (PFS), tumor response, and adverse events (AEs) were analyzed.

**Results:**

Among 814 patients, 566 (69.5%) were <75 years (median age 64, 84% male) and 248 (30.5%) ≥75 years (median age 78, 86% male). After matching, 484 were analyzed. After a median follow-up of 28.0 months, OS and PFS were similar in ≥75 and <75 (15.4 *vs*. 16.07 months; *p* = 0.936), (7.2 vs. 6.5 months; *p* = 0.706) respectively. Age ≥ 75 years was not associated with PFS nor OS. In patients aged ≥75 years, modified albumin–bilirubin grade 3 (mALBI) was the only factor associated with disease progression (HR 4.37, 95% CI 2.04,9.37; *p* <0.001) and mortality (HR 5.62, 95% CI 2.47,12.8; *p* <0.001). In mALBI 3 median OS and PFS were 5.43 and 2.3 months, respectively. Immune-related AEs were less frequent in ≥75 including (22.1% *vs.* 36.9%; *p* <0.001) or excluding dysthyroidism (15.9% *vs.* 25.9%; *p* = 0.01). In univariate analysis, OS and PFS were longer in patients ≥75 years who developed hypertension (*p* = 0.04 and *p* = 0.09), proteinuria (*p* <0.0001 and *p* = 0.015) and Immune-related AEs (*p* = 0.02 and *p* = 0.007). Hypertension during treatment was associated with proteinuria (odds ratio = 13.1; 95% CI 4.1–42.4), without difference at baseline (*p* = 0.35).

**Conclusions:**

AtezoBev is effective and safe in patients ≥75 years but mALBI 3 warrants particular caution.

**Impact and implications:**

Very elderly patients (≥75) are underrepresented in immunotherapy trials, leaving uncertainty about the benefit–risk of atezolizumab plus bevacizumab in this subgroup. In real-world practice, European ancestry patients ≥75 with advanced hepatocellular carcinoma are common. Our study indicates that atezolizumab–bevacizumab is generally safe and effective in this population, with mALBI grade 3 as a notable high-risk exception. Although retrospective, the use of propensity score methods mitigates confounding and supports the robustness of our conclusions.

**Clinical trials registration number:**

NCT06416683.

## Highlights

• Patients aged ≥75 years with advanced hepatocellular carcinoma are common.
• Evidence on the safety and efficacy of atezolizumab plus bevacizumab in this age group remains limited.
• Overall survival and progression-free survival appear comparable between patients aged <75 and those ≥75 years.
• Particular caution is warranted in patients aged ≥75 years with mALBI grade 3.

## Graphical abstract

**Figure:**
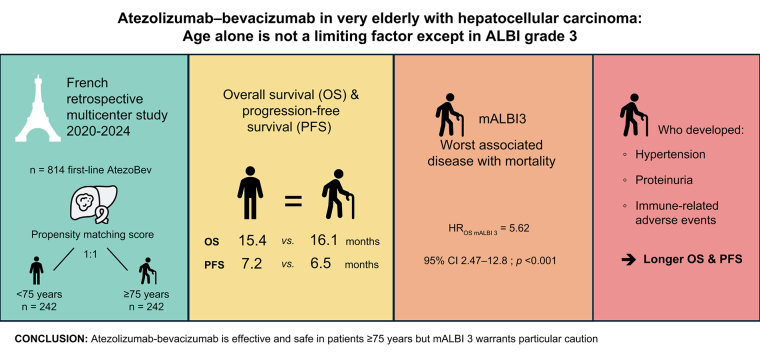

## Introduction

Primary liver cancer is the sixth most common cancer and the third leading cause of cancer-related death worldwide, with hepatocellular carcinoma (HCC) accounting for 75–85%.^1^ Half of patients are diagnosed at an advanced and/or unresectable stage, for which immunotherapy has become the standard first-line systemic therapy since the publication of the IMbrave150 trial in 2020.^2^^,^^3^ In this study, the median age was 64 years, whereas French national registry reports a median age at HCC diagnosis of 69 years in men and 73 years in women.^4^

Despite immunotherapy widespread adoption, pivotal trials included few elderly patients, and data on the safety and efficacy of immune checkpoint inhibitor therapy in this population remain limited. A subgroup analysis of the IMbrave150 trial confirmed overall survival (OS) and progression-free survival (PFS) benefit in patients aged ≥65 years with a similar safety profile, but 55 patients only were older than 75 years.^5^ Japanese studies have reported similar efficacy and good tolerability of immunotherapy (>90% atezolizumab plus bevacizumab [AtezoBev]) beyond 75 years of age^6^^,^^7^ while an international study by Vithayathil *et al.*^8^ showed no significant OS differences according to age, albeit using a 65-year cut-off. In the French CHIEF cohort study, the mean age was 69 years, with only 49 patients over 70 years.^9^

In real-world practice, elderly patients are frequently underrepresented or excluded from immunotherapy, leading to uncertainty about the benefit–risk balance of AtezoBev in this subgroup. Given the frailty, multiple comorbidities and modified pharmacokinetics^10^^,^^11^ associated with advanced age, further evidence is needed to guide treatment decisions. The aim of the study was to assess, in real-world practice, the efficacy, safety, and prognostic factors of AtezoBev in European ancestry patients aged ≥75 years with advanced and/or unresectable HCC, compared with those younger than <75 years.

## Materials and methods

### Study design

This was a retrospective, observational, multicenter study conducted across eight academic tertiary care setting centers in France (Beaujon Hospital, Avicenne Hospital, La Pitié-Salpêtrière Hospital, Henri Mondor Hospital, Saint Antoine Hospital, Sud Francilien Hospital Center, Toulouse University Hospital, and Caen University Hospital) over the period from January 2020 to December 2024. In accordance with Commission nationale de l'informatique et des libertés (CNIL) guidelines, the database was pseudo-anonymized and recorded on the secure server of the University Hospital of Caen with restricted access to the project team. This research project constitutes ‘Research on Data’ according to the Article L. 1121-1 of the Public Health Code (Law No. 2012-3000 of March 5, 2012, and its implementing decree No. 2016-1537 of November 16, 2016). This automated processing of health data complies with the European Regulation of April 27, 2016, concerning the protection of individuals regarding the processing of personal data and the free movement of such data. The protocol was approved by the following Research Ethics Committees (ID Number 2024112619344200000180001697, APHP210978/IDRCB Number: 2021-A01343-38).

### Patient selection

Adults (≥18 years) who received first-line AtezoBev at participating centers, identified through pharmacy records, were eligible if they had advanced or unresectable HCC confirmed histologically or on imaging according to EASL criteria.^2^ When no hepatic target was available, diagnosis was confirmed on histological analysis of metastasis. Cirrhosis was diagnosed based on histological findings or a combination of clinical, radiological, and biological criteria, and its etiology was documented by the treating physician. Patients with benign liver tumors, other primary liver malignancies, or prior systemic therapy were excluded. Patients aged 75 years or older were considered elderly.

### Data collection

For each patient, baseline clinical, radiological, and laboratory data were collected immediately before initiation of AtezoBev. Variables included age, sex, comorbidities (type 2 diabetes, arterial hypertension, body mass index [BMI]), etiology of liver disease, presence of cirrhosis and its complications; tumor characteristics (number of nodules, size of the largest lesion, macrovascular invasion, and extrahepatic metastases); Barcelona Clinic Liver Cancer (BCLC) stage; and liver function parameters (Child–Pugh score and modified albumin–bilirubin [mALBI] grade). The ALBI grade was calculated based on serum albumin and total bilirubin values using the following formula: ALBI score: (log_10_ bilirubin (μmol/L) × 0.66) + (albumin (g/L) × -0.085). ALBI grade was defined by the score of the following: ≤-2.60 = grade 1, >-2.60 to ≤-1.39 = grade 2, >-1.39 = grade 3. ALBI grade 2 was further divided into two subgrades (2a and 2b) using ALBI score -2.270 as the cut-off value. The four ALBI grades were named as mALBI grade. Blood tests comprised total bilirubin, INR, alpha-fetoprotein (AFP), albumin, creatinine, and platelet count. The diagnosis of metabolic dysfunction-associated steatotic liver disease (MASLD) was made according to the presence of hepatic steatosis (identified either through imaging or histological analysis) in combination with at least one cardiometabolic risk factor (current or prior BMI >30 kg/m^2^, type 2 diabetes mellitus, hypertension, or abnormal lipid profiles) and none or low alcohol intake (<20 g/day in women and <30 g/day in men). Cirrhosis was defined as having at least an alcoholic, viral, or metabolic etiology when it was related, respectively, to chronic alcohol consumption, viral infection, or metabolic syndrome, with or without an associated additional cause of chronic liver disease (alcoholic, viral, metabolic). During follow-up, adverse events (AEs) were graded according to the Common Terminology Criteria for AEs, version 5.0. Tumor response to treatment was assessed and categorized as complete response (CR), partial response (PR), stable disease (SD), or progressive disease (PD) according to RECIST 1.1 criteria.^2^ Objective response rate (ORR) was defined as CR or PR, while disease control rate (DCR) was defined as CR, PR, or SD considering the best response during follow-up. In addition, subsequent treatments administered after AtezoBev were also collected. Patients were followed until death, progression, or December 20, 2024 for patients who remained alive and progression-free. The causes of death were recorded, as well as the time between death and the end of treatment, when applicable.

### Statistical analysis

Continuous variables are described as median and IQR, and categorical variables as numbers and percentages. The non-parametric Mann–Whitney *U* test was used to compare quantitative variables and Χ^2^ test for categorical variables. Follow-up of the patients was recorded until December 20, 2024. OS was calculated from the date of AtezoBev initiation to the date of death or at the time of the last follow-up. PFS was calculated from the date of AtezoBev initiation to the date of disease progression or death. Kaplan–Meier curves and the log-rank test were used to evaluate OS and PFS. Univariable and multivariable survival analyses were carried out using the Cox model. Variables included in the multivariable models were selected based on univariable analyses, and all covariates with a value of *p* <0.05 were entered into the multivariable models. Collinearity among covariates was evaluated using variance inflation factors (VIFs), and no significant multicollinearity was observed, with all VIF values <5. The proportional hazards assumption was assessed using Schoenfeld residuals. To account for baseline differences between very elderly patients (≥75 years) and younger counterparts (<75 years), a propensity score matching (PSM) approach was applied. The propensity score for being aged ≥75 years was estimated using a multivariable logistic regression model including clinically relevant covariates: presence of cirrhosis, serum albumin and bilirubin concentrations, size of the largest tumor nodule, presence of more than three nodules, extrahepatic metastases, macrovascular invasion, viral etiology (hepatitis B and C), alcohol- or metabolism-related etiology, and history of hepatic decompensation. Eastern Cooperative Oncology Group (ECOG) performance status was not included in the propensity score model because of the high proportion of missing data, which precluded reliable imputation. Missing data for covariates included in the propensity score model were imputed before matching. Continuous variables with <10% missing data were imputed using the median value, and categorical variables with >10% missing data were imputed using the most frequent category (mode). Matching was performed using the nearest-neighbor method without replacement, with a 1:1 ratio and a caliper width of 0.2 of the standard deviation of the logit of the propensity score. Baseline characteristics were compared before and after matching and graphical assessment of balance was performed using density plots and Love plot (Supplementary Data 1). A two-sided value of *p* <0·05 was considered statistically significant. All statistical analyses were performed using R version 4.5.1 (R Foundation for Statistical Analysis, Vienna, Austria).

## Results

### Baseline characteristics of patients

Between January 2020 and December 2024, 814 patients with advanced and/or unresectable HCC were treated with AtezoBev as first-line systemic therapy. A total of 169 patients were included from Beaujon Hospital, 140 patients from Avicenne Hospital, 125 patients from La Pitié-Salpêtrière Hospital, 109 patients from Henri Mondor Hospital, 64 patients from Saint Antoine Hospital, six patients from Sud Francilien Hospital Center, 96 patients from Toulouse University Hospital, and 105 patients from Caen University Hospital. No differences in patient characteristics were observed between centers (Supplementary Data 2). Of these, 566 patients (69.5%) were <75 years (median age 64; 84% male) and 248 (30.5%) were ≥75 years, defined as the elderly group (median age 78; 86% male) (Supplementary Data 3). Compared with non-elderly patients, elderly patients had more arterial hypertension (75% *vs.* 54%), dyslipidemia (36% *vs.* 22%), and anticoagulation use (23% *vs.* 15%), but less cirrhosis (65% *vs.* 78%), fewer prior decompensations (12% *vs.* 24%), and less viral etiology (22% *vs.* 48%). Patients aged ≥75 years had more Child–Pugh A disease (90% *vs.* 80%); tumor burden was broadly similar, but AFP was lower (median 54 *vs.* 95 ng/ml) and vascular invasion less frequent (27% *vs.* 42%) (Supplementary Data 4). Median OS (<75 years *vs.* elderly: 14.4 *vs.* 15.5 months) and PFS (<75 years *vs.* elderly: 6.1 *vs.* 7.3 months) were similar regardless of the age status in the whole cohort Supplementary Data 5.

Owing to significant differences in liver function, cause of liver disease, and HCC burden, we performed PSM and 484 patients were included in the final analysis (Supplementary Data 1). After matching, patients aged ≥75 years more often received anticoagulation (22% *vs.* 10%; *p* <0.001), had more hypertension (74% *vs.* 60%; *p* <0.001) and less obesity (18% *vs.* 26%; *p* = 0.043), while liver disease parameters and HCC characteristics were otherwise comparable between groups (Table 1).

**Table 1:** Baseline characteristics of patients treated with AtezoBev included in the analysis after propensity score matching (n = 484).

Baseline characteristics	Available data	Elderly patients (≥75 years) n = 242	Available data	Non-elderly patients (<75 years) n = 242	*p* value
Sex (male)	242	209 (86)	242	203 (84)	0.443
Body mass index (kg/m^2^) °	239	26.0 (23.8, 29.1)	242	26.0 (22.5, 30.1)	0.669
Obesity	239	43 (18)	241	62 (26)	**0.043**
Type 2 diabetes	242	117 (48)	242	104 (43)	0.236
Arterial hypertension	242	179 (74)	242	144 (60)	**<0.001**
Dyslipidemia	242	88 (36)	242	70 (29)	0.081
Anticoagulation	237	53 (22)	233	24 (10)	**<0.001**
Cirrhosis	242	160 (66)	242	171 (71)	0.252
ECOG 0–1 ECOG 2–3	224	199 (89) 25 (11)	200	186 (93) 14 (7)	0.139
Etiologies of liver disease					
At least ALD	242	107 (44)	242	99 (41)	0.462
At least MASLD	242	110 (45)	242	110 (45)	>0.999
At least viral	242	54 (22)	242	67 (28)	0.172
Mixed etiologies	242	99 (41)	242	97 (40)	0.853
Liver function					
Previous cirrhosis decompensation	238	28 (12)	234	24 (10)	0.567
MELD		8.0 (7.0, 10.0)		8.0 (7.0, 10.0)	
Child–Pugh A	239	214 (90)	240	219 (91)	0.485
Child–Pugh B		25 (10)		20 (8.6)	
Child–Pugh C		0 (0)		1 (0)	
mALBI grade 1	233	69 (30)	232	86 (37)	**0.038**
mALBI grade 2a		73 (31)		46 (20)	
mALBI grade 2b		83 (36)		92 (40)	
mALBI grade 3		8 (3.4)		8 (3.4)	
No EV	225	138 (61)	231	138 (60)	0.876
EV (regardless the size)		88 (39)		92 (40)	
Large size EV		41 (18)		44 (19)	0.317
Platelet count ( × 10^3^/mm^3^)	237	179 (125, 250)	234	173 (117, 252)	0.547
Creatinine (μmol/L)	235	81 (66, 103)	234	70 (59, 87)	**<0.001**
Total bilirubin (μmol/L)	236	12 (9, 19)	233	13 (9, 18)	0.472
Albumin (g/L)	233	36 (33, 39)	236	36 (33, 40)	0.566
INR°	227	1.1 (1.0, 1.2)	222	1.1 (1.0, 1.2)	0.148
HCC features					
Previous HCC treatment	220	145 (66)	235	151 (64)	0.712
BCLC-A	241	2 (1)	242	4 (2)	0.173
BCLC-B		80 (33)		93 (38)	
BCLC-C		160 (66)		145 (60)	
AFP (ng/ml)	229	59 (5, 9)	234	31 (6, 6)	0.801
AFP >20 ng/ml	229	132 (58)	234	123 (53)	0.272
AFP >400 ng/ml	229	74 (32)	234	67 (29)	0.389
>3 lesions	241	129 (54)	238	120 (51)	0.591
Size of the largest lesion (mm)	223	51 (24, 80)	214	47 (24, 80)	0.684
Tumor size >5 cm	223	116 (52)	214	105 (49)	0.537
Extrahepatic lesions	242	74 (31)	242	71 (29)	0.766
Vascular invasion	242	65 (27)	242	69 (29)	0.684
AtezoBev treatment					
Treatment duration (months)	242	4.8 (1.5, 12)	242	5.1 (2.1, 11.7)	0.207
Second-line treatment	205	62 (30)	203	79 (39)	0.066

Data are presented as n (%) or median (IQR). *p* values in bold indicate statistical significance (*p* <0.05). AFP, alpha-fetoprotein; ALD, alcoholic liver disease; AtezoBev, atezolizumab–bevacizumab; BCLC: Barcelona Clinic Liver Cancer; ECOG, Eastern Cooperative Oncology Group; EV, esophageal varices; HCC, hepatocellular carcinoma; INR, international normalized ratio; mALBI grade, modified albumin–bilirubin grade; MASLD, metabolic dysfunction-associated steatotic liver disease; MELD, model for end-stage liver disease.

### Comparable outcomes in patients ≥75 years *vs*. <75 years after PSM

**Fig. 1:**
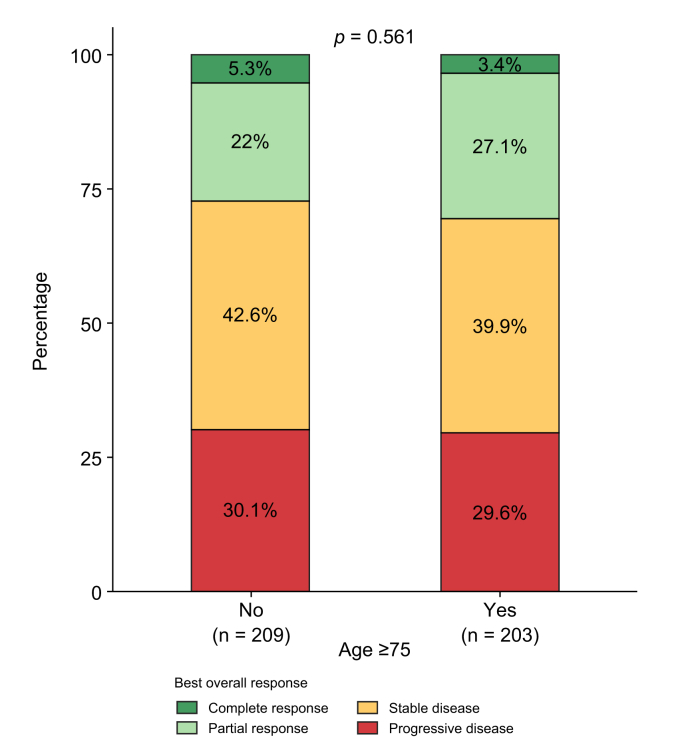
Overall response and disease control by age group (n = 412). Stacked bar plots compare outcomes between patients aged <75 years (‘No’) and ≥75 years (‘Yes’) after propensity score matching. Distribution of best overall response categories (Complete Response, Partial Response, Stable Disease, Progressive Disease). Percentages of each class are reported within the bars, and sample sizes (n) are shown for each age group on the x-axis. Only patients who underwent radiological tumor assessment were included; patients who died or experienced symptomatic progression before radiological evaluation were excluded corresponding to the exclusion of 33 non-elderly and 39 elderly patients, respectively.

Median duration of AtezoBev was similar between patients ≥75 years (4.8 months [1.5–12.0]) and those <75 years (5.1 months [2.1–11.7]) (*p* = 0.207). Atezolizumab was continued as monotherapy in 21% and 24% of patients, respectively (*p* = 0.162). The best response rate was also comparable (Fig. 1): ORR 30.5% *vs.* 27.3% and DCR 70.4% *vs.* 69.9% in ≥75 *vs.* <75 years, respectively.

**Fig. 2:**
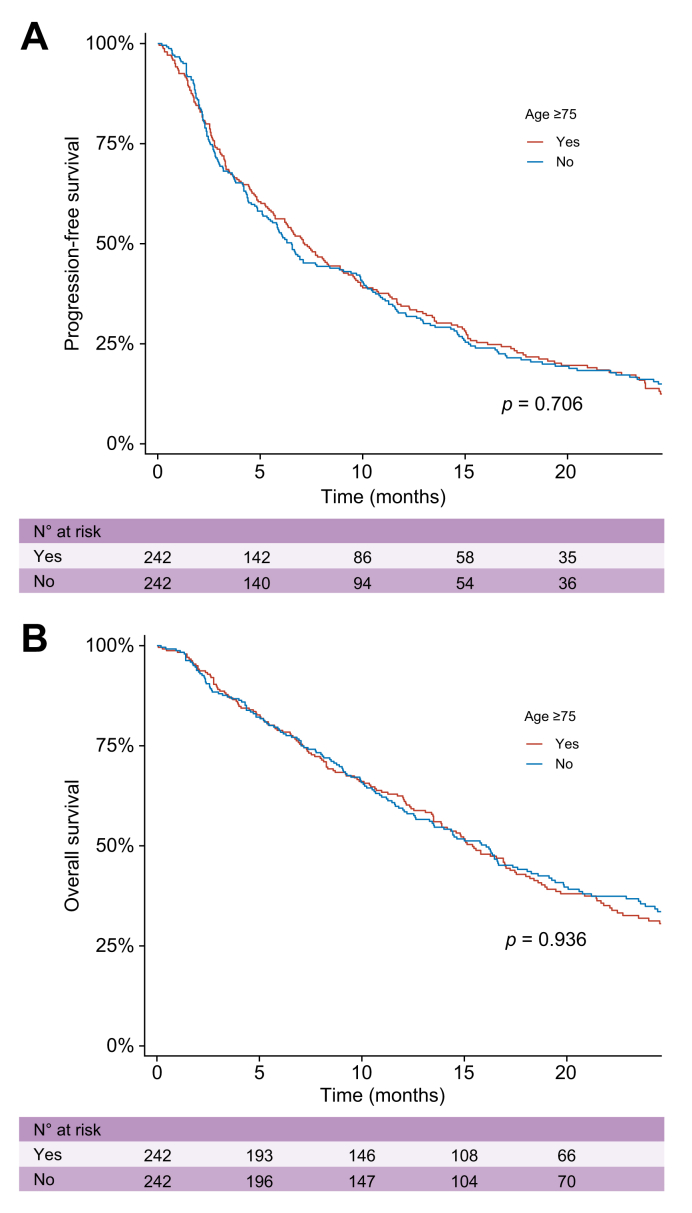
Kaplan–Meier curves for progression-free and overall survival according to age group. Kaplan–Meier estimates of progression-free survival (A) and overall survival (B) according to age group (<75 years *vs.* ≥75 years) after propensity score matching. Survival curves were compared using the log-rank test, n = 412.

After a median follow-up of 28.0 months (95% CI 26.6–30.0), median PFS was 6.7 months (6.0, 8.0) in the whole cohort, 7.2 months (6.2, 9.0) in patients ≥75 years, and 6.5 months (5.5, 9.1) in those <75 years (*p* = 0.706; Fig. 2A).

In the whole cohort, mALBI grade 2b (HR 1.59; 95% CI 1.22, 2.06; *p* <0.001) and mALBI grade 3 (HR 3.77; 95% CI 2.12–6.70; *p* = 0.006), as well as the presence of extrahepatic metastases (HR 1.48; 95% CI 1.17–1.88; *p* = 0.001) and AFP >400 ng/ml (HR 1.48; 95% CI 1.16, 1.88; *p* = 0.002), were independently associated with PFS in multivariate analysis, but not an age ≥75 years (Supplementary Data 6). A trend toward an association was also observed for ECOG 2–3 (HR 1.42; 95% CI 0.97–2.08; *p* = 0.068.

Across 325 deaths, cause distribution was similar in patients ≥75 and <75 years: HCC progression 49.7% *vs.* 48.8%, liver decompensation 8.1% *vs.* 6.7%, non-liver causes 1.9% *vs.* 1.8%, with unknown cause in 40.4% *vs.* 42.7%. Median OS was 15.8 months (14.0, 17.0) in the whole cohort, 15.4 months (13.5, 17.5) in elderly patients and 16.1 months (13.4, 18.6) in those <75 years (*p* = 0.936; Fig. 2B). In the multivariate analysis, mALBI grade 2b (HR 1.60; 95% CI 1.13, 2.26; *p* = 0.007) and mALBI grade 3 (HR 4.58; 95% CI 2.36, 8.88; *p* <0.001), as well as ECOG 2–3 (HR 1.56; 95% CI 1.01–2.42; *p* = 0.046) and AFP >400 ng/ml (HR 1.46; 95% CI 1.09, 1.96; *p* = 0.011), were independently associated with mortality, but not an age ≥75 years (Supplementary Data 7).

### mALBI grade 3 after PSM is predictive of poorer outcomes in patients ≥75 years treated with AtezoBev

**Fig. 3:**
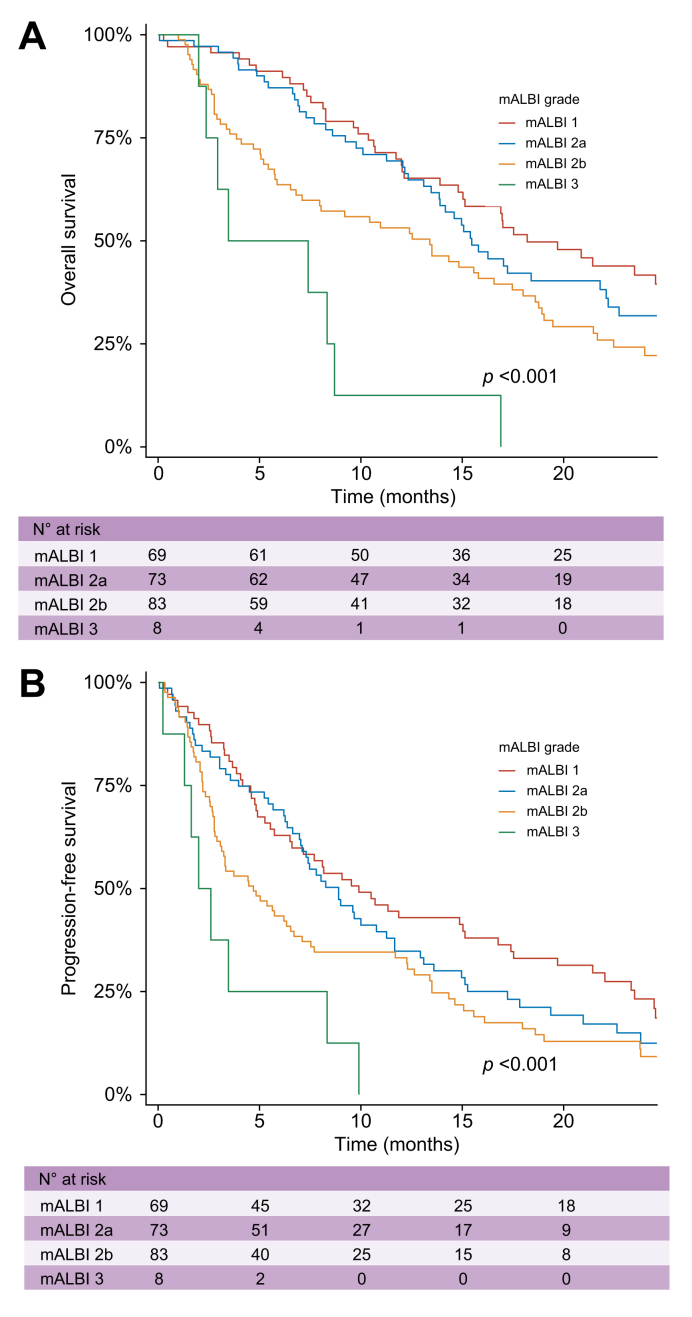
Overall and progression-free survival according to mALBI grade in the elderly group. Kaplan–Meier curves showing (A) overall survival (OS) and (B) progression-free survival stratified by mALBI grade at treatment initiation in patients ≥75 years old after propensity score matching. The number of patients at risk is shown below each plot. mALBI, modified albumin–bilirubin grade.

**Fig. 5:**
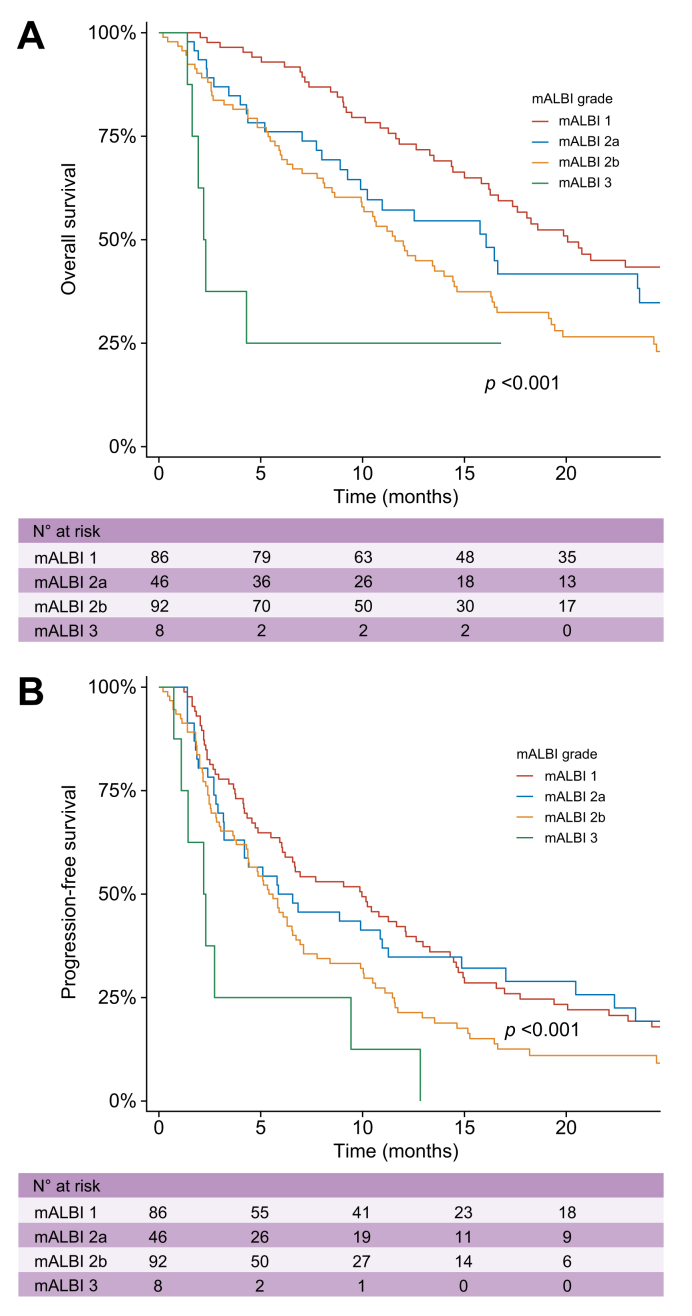
Overall and progression-free survival according to mALBI grade in the non-elderly group. Kaplan–Meier curves showing (A) overall survival and (B) progression-free survival stratified by mALBI grade at treatment initiation in patients <75 years old after propensity score matching. The number of patients at risk is shown below each plot. mALBI: modified albumin–bilirubin grade.

In the cohort of patients aged ≥75 years treated with AtezoBev (n = 242), univariate analysis identified several baseline factors associated with both progression and mortality, particularly parameters reflecting liver function (Table 2, Table 3). A clear gradient in mortality was observed across mALBI grades (*p* <0.001), ranging from 55.1% in mALBI 1 to 100% in mALBI 3 (four from HCC progression, four unknown). The mALBI classification showed a clear stepwise association with survival outcomes. In multivariable Cox models adjusted for significant covariates, the mALBI grade remained independently associated with both PFS and OS, confirming that impaired hepatic reserve strongly influences long-term outcomes in elderly patients receiving AtezoBev. Compared with patients with mALBI 1, those classified as mALBI 3 exhibited significantly higher risks of disease progression (HR 4.37, 95% CI 2.04, 9.37, *p* <0.001) (Table 2) and death (HR 5.62, 95% CI 2.47, 12.8, *p* <0.001) (Table 3). In mALBI 3 median OS and PFS were 5.43 months and 2.3 months respectively (Fig. 3). Baseline characteristics of patients with an mALBI score of 3 were similar between elderly and non-elderly patients, except for a higher prevalence of dyslipidemia and use of beta-blockers among elderly patients (Supplementary Data 8). mALBI grade 3 was also associated with a poor prognosis in non-elderly patients (Fig. 5).

**Table 2:** Baseline predictive factors of progression-free survival in patients aged ≥75 years treated with AtezoBev included in the analysis after propensity score matching (Cox proportional hazard regression models, n = 242).

	Patients	Events	Univariate analysis			Multivariate analysis		
			HR	95% CI	*p* value	HR	95% CI	*p* value
Sex (male)	209	170	0.77	0.52, 1.13	0.186			
Obesity	239	197	0.97	0.67, 1.41	0.878			
Type 2 diabetes	243	200	0.87	0.66, 1.15	0.340			
Arterial hypertension	242	200	0.76	0.55, 1.06	0.104			
Dyslipidemia	242	200	1.18	0.89, 1.57	0.254			
Anticoagulation	237	195	0.91	0.65, 1.27	0.572			
Cirrhosis	242	200	0.85	0.63, 1.14	0.276			
ECOG 0–1	199	164	—	—	—			
ECOG 2–3	25	22	1.28	0.82, 2.00	0.274			
At least ALD	242	200	1.07	0.81, 1.41	0.637			
At least MASLD	242	200	1.03	0.78, 1.37	0.815			
At least viral	242	200	0.89	0.63, 1.24	0.484			
Mixed etiologies	242	200	0.99	0.75, 1.32	0.960			
Esophageal varices	225	186	1.12	0.84, 1.50	0.452			
Large size EV	41	36	1.07	0.91, 1.27	0.400			
Previous cirrhosis decompensation	237	196	1.54	0.97, 2.46	0.067			
Platelet count	237	196	1.00	1.00, 1.00	0.234			
Albumin	233	193	0.94	0.91, 0.97	**<0.001**			
Total bilirubin	236	195	1.02	1.00, 1.03	**0.025**			
Creatinine	235	194	1.00	1.00, 1.00	0.530			
INR	227	186	1.57	0.83, 2.93	0.162			
MELD	231	190	1.01	0.97, 1.05	0.537			
Child–Pugh A	214	175	-2.24	-1.44, 3.48	**-<0.001**			
Child–Pugh B	25	23						
mALBI grade 1	69	52	—	—	—	—	—	—
mALBI grade 2a	73	59	1.32	0.90, 1.92	0.150	1.36	0.92, 2.01	0.124
mALBI grade 2b	83	74	1.77	1.24, 2.53	**0.002**	1.81	1.25, 2.62	**0.002**
mALBI grade 3	8	8	4.14	1.94, 8.81	**<0.001**	4.37	2.04, 9.37	**<0.001**
Previous HCC treatment	220	185	1.14	0.84, 1.56	0.404			
BCLC-A	1	1	—	—	—			
BCLC-B	80	63	0.13	0.02, 0.93	0.052			
BCLC-C	160	136	0.16	0.02, 1.19	0.073			
AFP (ng/ml)	229	187	1.00	1.00, 1.00	**0.030**	1.00	1.00, 1.00	**0.012**
AFP >20 ng/ml	229	110	1.32	0.98, 1.76	0.064			
AFP >400 ng/ml	229	65	1.31	0.97, 1.77	0.083			
>3 lesions	241	200	1.09	0.83, 1.44	0.540			
Size of the largest lesion (mm)	223	183	1.00	1.00, 1.00	0.324			
Tumor size >5 cm	223	183	0.91	0.68, 1.21	0.507			
Extrahepatic lesions	242	200	1.32	0.98, 1.79	0.068			
Vascular invasion	242	200	1.01	0.74, 1.38	0.928			

The ECOG performance status was not included in the multivariate analysis because the proportion of missing values exceeded the predefined 10% threshold for imputation. *p* values in bold indicate statistical significance (*p* <0.05).

The Child–Pugh score, MELD score, albumin, and bilirubin were not entered in the multivariate analysis to avoid collinearity with ALBI grade. AFP, alpha-fetoprotein; ALBI, albumin–bilirubin grade; ALD, alcoholic liver disease; AtezoBev, atezolizumab–bevacizumab; BCLC: Barcelona Clinic Liver Cancer; EV, esophageal varices; HCC, hepatocellular carcinoma; HR, hazard ratio; INR, international normalized ratio; mALBI grade, modified albumin–bilirubin grade; MASLD, metabolic dysfunction-associated steatotic liver disease; MELD, model for end-stage liver disease.

**Table 3:** Baseline predictive factors of mortality in patients aged ≥75 years treated with AtezoBev included in the analysis after propensity score matching (Cox proportional hazard regression models, n = 242).

	Patients	Events	Univariate analysis			Multivariate analysis		
			HR	95% CI	*p* value	HR	95% CI	*p* value
Sex (male)	242	140	1.02	0.64, 1.61	0.948			
Obesity	239	158	0.81	0.52, 1.26	0.351			
Type 2 diabetes	243	161	0.91	0.67, 1.24	0.564			
Arterial hypertension	242	161	0.87	0.62, 1.22	0.424			
Dyslipidemia	242	161	1.36	0.99, 1.87	0.056			
Anticoagulation	237	156	1.05	0.73, 1.52	0.775			
Cirrhosis	242	161	0.84	0.60, 1.16	0.289			
ECOG 0–1	199	128	—	—	—			
ECOG 2–3	25	20	1.60	1.00, 2.57	0.051			
At least ALD	242	161	1.54	0.98, 2.41	0.062			
At least MASLD	242	161	0.79	0.52, 1.19	0.256			
At least viral	242	161	0.67	0.39, 1.15	0.143			
Mixed etiologies	242	161	1.06	0.78, 1.45	0.697			
Esophageal varices	225	149	1.21	0.87, 1.67	0.259			
Large size EV	41	30	1.11	0.92, 1.33	0.280			
Previous cirrhosis decompensation	237	157	1.28	0.76, 2.14	0.356			
Platelet count	237	159	1.00	1.00, 1.00	0.270			
Albumin	233	158	0.93	0.90, 0.96	**<0.001**			
Total bilirubin	236	158	1.01	1.00, 1.03	**0.040**			
Creatinine	235	158	1.00	1.00, 1.00	0.633			
INR	227	152	1.92	1.07, 3.43	**0.029**	1.42	0.69, 2.93	0.337
MELD	231	156	1.04	1.00, 1.07	0.062			
Child–Pugh A	214 25	137	—	—	—			
Child–Pugh B		22	2.24	1.42–3.54	**<0.001**			
mALBI grade 1	69	38	—	—	—	—	—	**–**
mALBI grade 2a	73	51	1.49	0.98, 2.28	0.062	1.59	1.02, 2.50	**0.043**
mALBI grade 2b	83	61	1.91	1.27, 2.88	**0.002**	1.90	1.20, 2.93	**0.006**
mALBI grade 3	8	8	5.53	2.54, 12.0	**<0.001**	5.62	2.47, 12.8	**<0.001**
Previous HCC treatment	220	148	0.81	0.58, 1.13	0.210			
BCLC-A	1	1	—	—	—			
BCLC-B	80	46	0.12	0.02, 0.91	**0.040**			
BCLC-C	160	114	0.18	0.02, 1.32	0.092			
AFP (ng/ml)	229	150	1.00	1.00, 1.00	**<0.001**			
AFP >20 ng/ml	229	92	1.44	1.03, 2.00	**0.031**	1.38	0.98, 1.94	0.068
AFP >400 ng/ml	229	59	1.30	0.93, 1.82	0.124			
>3 lesions	241	161	1.03	0.75, 1.40	0.865			
Size of the largest lesion (mm)	223	148	1.00	1.00, 1.00	0.247			
Tumor size >5 cm	223	148	1.32	0.95, 1.82	0.096			
Extrahepatic lesions	242	161	1.25	0.89, 1.75	0.194			
Vascular invasion	242	161	1.29	0.92, 1.81	0.136			

ECOG performance status was not included in the multivariate analysis because the proportion of missing values exceeded the predefined 10% threshold for imputation. *p* values in bold indicate statistical significance (*p* <0.05). Child–Pugh score, MELD score, albumin, and bilirubin were not entered in the multivariate analysis to avoid collinearity with ALBI grade. AFP, alpha-fetoprotein; ALD, alcoholic liver disease; AtezoBev, atezolizumab–bevacizumab; BCLC, Barcelona Clinic Liver Cancer; EV, esophageal varices; HCC, hepatocellular carcinoma; INR, international normalized ratio; mALBI grade, modified albumin–bilirubin grade; MASLD, metabolic dysfunction-associated steatotic liver disease; MELD, model for end-stage liver disease.

### Distribution of adverse events in ≥75 years after PSM

**Fig. 4:**
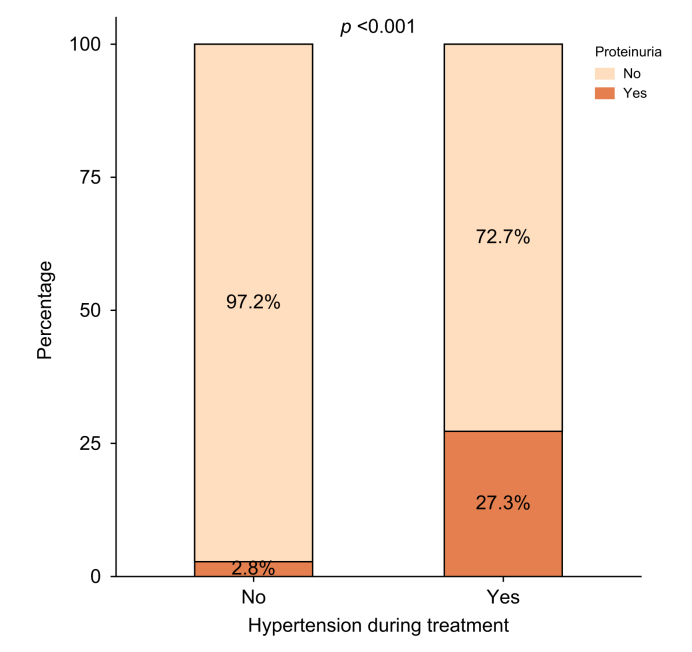
Association between hypertension and proteinuria during treatment. Stacked bar plot showing the proportion of patients who developed proteinuria according to the presence or absence of hypertension during treatment.

Any grade AEs occurred in 80% and 75% of patients, in <75 years and ≥75 years (*p* = 0.254). Acute variceal bleeding occurred in 16% and 13% (*p* = 0.336). The only difference between groups concerned the immune-related adverse events (IRAEs) which were more frequent in patients <75 years compared with those ≥75 years, both when including thyroid dysfunction (37% *vs.* 22%; *p* <0.001) and when excluding it (25.9% *vs.* 15.9%; *p* = 0.010) (Table 4). In univariate analysis among the 242 elderly patients, OS and PFS were longer in those who developed hypertension (*p* = 0.040 and *p* = 0.090, respectively), proteinuria (*p* <0.001 and *p* = 0.015, respectively) and IRAEs (*p* = 0.020 and *p* = 0.007, respectively). Among them, 39 developed on-treatment hypertension and 184 did not. Baseline hypertension was similar in the two groups (79% [31/39] *vs.* 72% [133/184]; *p* = 0.354) (Supplementary Data 9). Proteinuria occurred in 27.3% of patients who developed hypertension during treatment, compared with only 2.8% among those without hypertension (*p* <0.001) (Fig. 4). The presence of hypertension was strongly associated with the occurrence of proteinuria (OR [odds ratio] = 13.1; 95% CI 4.1–42.4). Treatment duration was longer in patients who developed hypertension (median 8.3 months [2.8–14.8] *vs.* 4.1 months [1.4–9.7]; *p* = 0.009). Notably, baseline hypertension did not predict on-treatment hypertension (*p* = 0.400) (Supplementary Data 10).

**Table 4:** Incidence of treatment-related adverse events in elderly *vs*. non-elderly patients treated with AtezoBev.

Adverse events	Overall N = 484 (%)	Elderly patients (≥75 years) n = 242 (%)	Non-elderly patients (<75 years) n = 242 (%)	*p* value
Any AE	337 (77)	168 (75)	169 (80)	0.254
Grade 3–4 AE	100 (20)	60 (21)	40 (19)	0.721
Asthenia	199 (46)	110 (50)	89 (41)	0.079
Ascites	94 (20)	52 (22)	42 (18)	0.268
Hepatic encephalopathy	38 (8)	20 (8.5)	18 (7.7)	0.756
Variceal bleeding	67 (14)	30 (13)	37 (16)	0.336
Arterial hypertension	66 (15)	39 (17)	27 (13)	0.144
Proteinuria	39 (9)	20 (9)	19 (9)	0.949
Hand–foot syndrome	11 (3)	5 (2)	6 (3)	0.720
Immune-related AE including dysthyroidism				
Any immune-related AE	130 (29)	50 (22)	80 (37)	**<0.001**
Steroid use	31 (25)	16 (31)	15 (21)	0.207
Hepatitis	10 (2)	4 (2)	6 (3)	0.538
Myositis	12 (3)	3 (1)	9 (4)	0.079
Skin rash	58 (13)	24 (11)	34 (16)	0.114
Thyroid dysfunction	51 (12)	19 (9)	32 (15)	**0.040**
Immune-related AE excluding dysthyroidism	92 (21)	36 (15.9)	56 (25.9)	**0.010**

AE, adverse events. *p* values in bold indicate statistical significance (*p* <0.05)

In addition, 20 patients also developed on-treatment proteinuria. Baseline hypertension was similar between patients who developed or who did not develop proteinuria under treatment (85% [17/20] *vs.* 72% [147/203]; *p* = 0.23) (Supplementary Data 11). However, hypertension occurred more frequently in those with proteinuria than in those without (55% *vs.* 14%; *p* <0.001). Treatment duration was also longer in patients who developed proteinuria (median 13.0 [5.1–15.2] months) than in those without proteinuria (4.2 [1.4–10.3 months]; *p* = 0.003).

IRAEs occurred in 130 patients. Notably, acute variceal bleeding was more common in those who developed such IRAEs (27% [13/50]) than in those who did not (8% [14/176]; *p* <0.001). Treatment duration was also longer in patients with IRAEs (median 10.6 [2.8–19.0] months *vs.* 4.1 [1.5–8.9] months; *p* <0.001) (Supplementary Data 12).

## Discussion

In our large European ancestry cohort, patients aged ≥75 years with advanced or unresectable HCC treated with AtezoBev had a median OS of 15.1 months, comparable to that in younger patients, without excess toxicity and similar response rate. However, mortality was significantly higher in elderly patients with mALBI grade 3, supporting cautious use of AtezoBev in this subgroup, while the occurrence of treatment-related AEs correlated with better outcomes.

Advanced age is associated with higher rates of tyrosine-kinase inhibitor dose reductions and treatment discontinuation, often because of intolerance, thereby challenging the initiation of systemic therapy in the elderly population.^11^^,^^12^ Consistent with previous reports, our results indicate that AtezoBev remains safe and effective in older patients without major bevacizumab discontinuation. However, real-world data in very elderly patients, particularly in populations of European ancestry, are scarce. Extending conclusions from studies with lower age cut-offs, smaller sample size, or Asian-only cohorts remains controversial given the frailty and comorbidities characteristics of this age group.^3^^,^[5], [6], [7], [8] In the recent Japanese propensity score matched study by Kaneko *et al.*,^7^ outcomes were similar between 112 patients aged ≥75 years and 112 patients aged <75 years, with a median OS of 25.6 months and 19.7 months respectively (*p* = 0.24). The longer survival observed in Japanese cohort compared with ours likely reflects differences in baseline characteristics, including a greater proportion of viral diseases (34% *vs.* 22%), earlier BCLC stages (BCLC B60.7% *vs.* 33 %), and a lower baseline AFP level (15.1 *vs.* 59 ng/ml) (7). Liver function, as reflected by mALBI grade, was independently associated with both OS and PFS. In our study mALBI 3 was the strongest prognostic factor for adverse outcomes, confirming its pivotal role in patients’ prognosis. Nevertheless, the original ALBI classification has recognized limitations, as most real-world patients cluster within ALBI 2, making this group highly heterogeneous in terms of residual liver function and prognosis. To overcome this limitation, the mALBI grade was developed. In a large cohort of patients with unresectable HCC treated with lenvatinib, median OS was approximately 22.5 months for mALBI 2a, 10.3 for mALBI 2b and 5.1 for mALBI3, while Child–Pugh failed to distinguish outcomes as effectively.^13^ Our findings confirm the independent prognostic value of mALBI even in the setting of AtezoBev, particularly among elderly patients. In our cohort aged ≥75 years, mALBI grades showed a stepwise and significant association with OS and PFS. Patients with mALBI 3 had a significantly greater risk of disease progression (HR 4.37) and mortality (HR 5.62) compared with those with mALBI 1, even after adjustment for clinical covariates.

In our series treatment duration and overall tolerability were comparable across age groups, except for immune-related AEs which were more frequent in younger patients. This observation may reflect immunosenescence and reduce immune-reactive profile beyond 75 years.^14^ Moreover, a similar proportion of elderly and younger patients received second-line therapy. Together, these findings support the safety of AtezoBev in very elderly European ancestry patients and confirm that immunotherapy does not compromise subsequent treatment. Treatment duration was longer among patients who developed hypertension, proteinuria or IRAEs (including or excluding thyroid dysfunction). No relationship was observed between baseline hypertension and the occurrence of hypertension or proteinuria during treatment. Yang *et al.*^15^ reported that lower baseline vascular endothelial growth factor-D in patients with proteinuria were related to a better prognosis^7^^,^^15^^,^^16^ suggesting that proteinuria may be a surrogate marker of effective anti-angiogenic treatment. IRAEs have already been correlated with improved radiological response, particularly when hypothyroidism develops, although associations with OS remain to be elucidated.^16^^,^^17^

Despite relative good tolerance and efficacy of AtezoBev in this series of very elderly patients, mALBI 3 could not really benefit from immunotherapy. Other recent evidence shows that systemic chemotherapy often provides little survival or functional benefit for very old, while causing substantial toxicity.^18^ Consequently, ASCO’s recent geriatric oncology guidance emphasizes routine geriatric assessment to identify vulnerabilities and to individualize or withhold systemic treatment when expected benefit is minimal, supporting avoidance of potentially futile, costly chemotherapy in appropriately selected very old patients.^19^ For this reason our results raise the question of AtezoBev futility in patients with altered hepatic function in this particular population.

The strengths of our study include its real-world, multicenter design within a European ancestry population, the large sample size and the use of PSM to minimize confounding. However, several limitations should be acknowledged. First, the retrospective design inherently limits data completeness and standardization across centers. Detailed information on previous antihypertensive therapy, treatment adherence, and dose adjustments of bevacizumab was not systematically available. Central radiological review could not be performed, which may have introduced variability in response assessment. In addition, multivariate analysis including time-dependent factors were not conducted, preventing assessment of how on-treatment events such as hypertension, proteinuria, or immune-related AE influenced survival outcomes. Lastly, although our results suggest a particularly poor prognosis in patients with mALBI grade 3, potentially questioning the initiation of immunotherapy in this subgroup, these findings must be interpreted with caution given the small sample size in both elderly and non-elderly patients. Larger studies are warranted to confirm these observations.

AtezoBev is efficient and safe as first-line treatment in very elderly patients of European ancestry with advanced and/or unresectable HCC when liver function is preserved. Caution is highly warranted in elderly patients with mALBI grade 3.

## Abbreviations

AEs, adverse events; AFP: alpha-fetoprotein; ALD, alcoholic liver disease; AtezoBev, atezolizumab–bevacizumab; BCLC, Barcelona Clinic Liver Cancer; BMI, body mass index; CR, complete response; DCR, disease control rate; ECOG, Eastern Cooperative Oncology Group; HCC, hepatocellular carcinoma; HR, hazard ratio; IRAEs, immune-related adverse events; mALBI, modified albumin–bilirubin grade; MASLD, metabolic dysfunction-associated steatotic liver disease; MELD, model for end-stage liver disease; OR, odds ratio; ORR, objective response rate; OS, overall survival; PD, progressive disease; PFS, progression-free survival; PR, partial response; PSM, propensity score matching; SD, stable disease; VIFs, variance inflation factors.

## Authors’ contributions

Study conceptualization: CM, IOH, MA. Acquisition of data: all authors. Data statistical analysis: CC, CM, RM. Data interpretation: CM, IOH, CC, MA. Drafted the manuscript: CM, IOH. Critical revision of the manuscript: all authors.

## Data availability

Data that support the findings of this study are available upon reasonable request from the corresponding author.

## Financial support

No financial support was received to produce this manuscript.

## Conflicts of interest

PN has received honoraria from and/or consults for AstraZeneca, Bristol-Myers Squibb, Eisai, and Roche. He received research grants from AstraZeneca, Bristol-Myers Squibb, and Eisai. IOH has received honoraria from and/or travel expenses from AstraZeneca, Roche, and AbbVie. JMP has received honoraria from AstraZeneca, Roche, AbbVie, Gilead, and IPSEN. CH has received honoraria from AbbVie, Roche, AstraZeneca, Jazz Pharmaceuticals, and Servier. PV has received honoraria from Roche. MB declares consulting fees from Bayer, MSD, Sirtex Medical, and Roche; advisory board fees from Bayer, MSD, Sirtex Medical, Eisai, AstraZeneca, Ipsen, Servier, Taiho, BMS, and Terumo, payment or honoraria for lectures from Bayer, Roche, MSD, Sirtex Medical, and AstraZeneca. CC declares research grants, speaker or travel expenses from Roche, Ipsen, AbbVie, and Gilead. J-CN has received research grants from Bayer and Ipsen. Other authors declare that they have no conflicts of interest related to the study. NG-C received travel and congress fees, consulting fees or honoraria for lectures, presentations, speakers bureaus from AbbVie, Gilead, and Roche.

Please refer to the accompanying ICMJE disclosure forms for further details.
