# Supplementary material for: Atezolizumab–bevacizumab in very elderly with hepatocellular carcinoma: Age alone is not a limiting factor except in ALBI grade 3
Source: JHEP Rep. 2026 Mar 17;8(6):101827. doi: 10.1016/j.jhepr.2026.101827 (PMC13195595; doi:10.1016/j.jhepr.2026.101827)
Supplement: Multimedia component 1 [file mmc1.pdf]

Atezolizumab–bevacizumab in very elderly with hepatocellular carcinoma: age alone is not a limiting factor except in ALBI grade 3

Chloé Métivier, Claudia Campani, Manon Allaire, Rémy Morello, Sarah Mouri, Eleonore Spitzer, Mohamed Bouattour, Clémence Hollande, Sabrina Sidali, Jean Charles Nault, Nathalie Ganne-Carrié, Pierre Nahon, Giuliana Amaddeo, Hélène Regnault, Paul Vigneron, Jean Marie Péron, Leila Sadek, Cecile Cussac, Marie Lequoy, Violaine Ozenne, Marie-Pierre Galais, Claire Pérignon, Louise Lebedel, Marion Habireche, Apolline Commin, Thông Dao, Charlotte Costentin, Aurore Baron, Isabelle Ollivier Hourmand

Table of contents

Supplementary data 1……………………………………………………………………….2

Supplementary data 2……………………………………………………………………….3

Supplementary data 3……………………………………………………………………….4

Supplementary data 4……………………………………………………………………….5

Supplementary data 5……………………………………………………………………….6

Supplementary data 6……………………………………………………………………….7

Supplementary data 7……………………………………………………………………….8

Supplementary data 8……………………………………………………………………….9

Supplementary data 9……………………………………………………………………...10

Supplementary data 10…………………………………………………………………….11

Supplementary data 11…………………………………………………………………….12

Supplementary data 12…………………………………………………………………….13

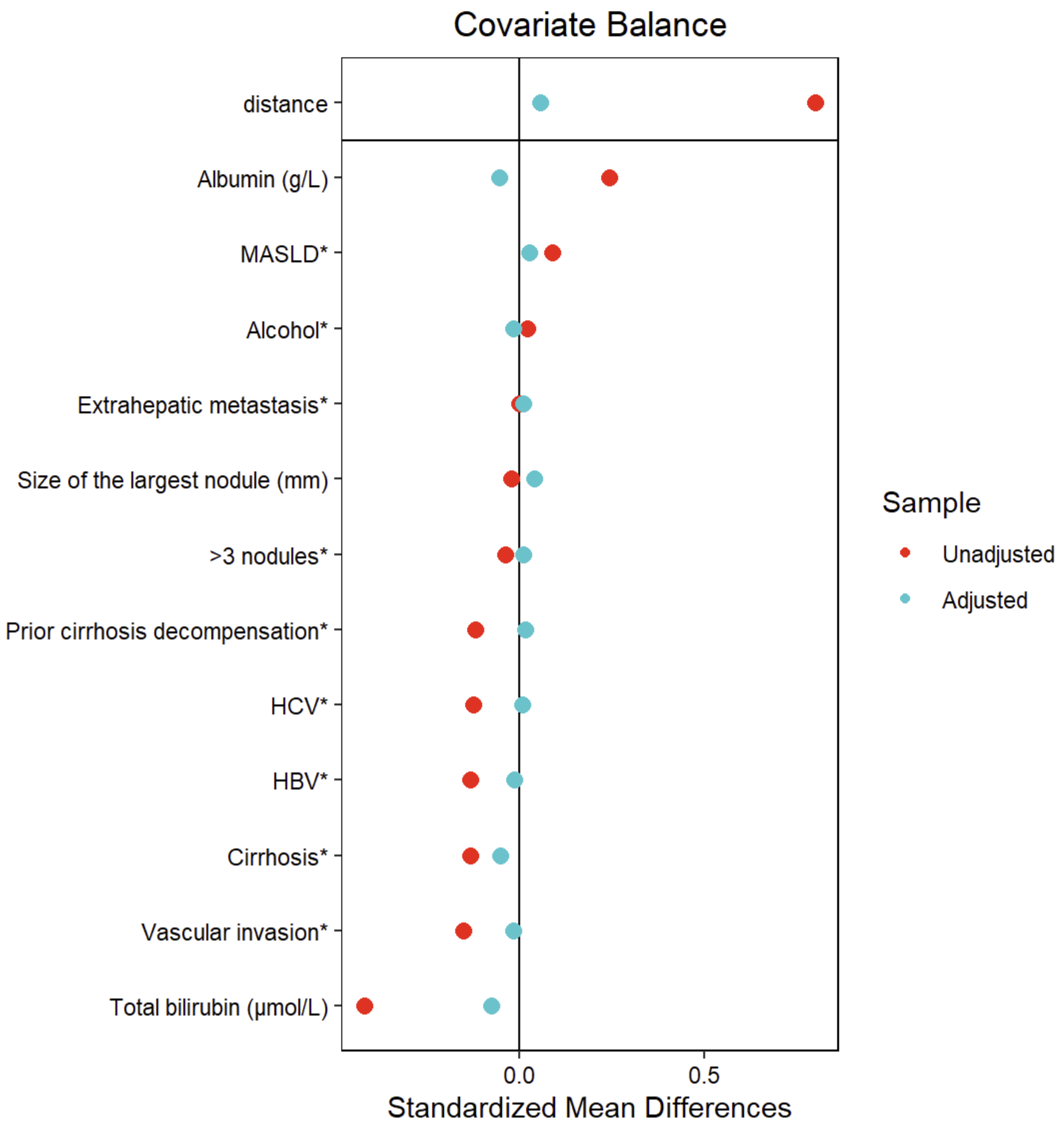

Supplementary data 1: Covariate balance before and after propensity score matching

Love plot showing standardized mean differences for baseline covariates before (red dots) and after (blue dots) matching. Each dot represents the difference in means (for continuous variables) or proportions (for categorical variables) between treatment groups. The vertical line at zero indicates perfect balance. After matching, all covariates demonstrated improved balance with standardized mean differences approaching zero. An asterisk (*) denotes categorical variables. HBV, hepatitis B virus; HCV, hepatitis C virus; MASLD, metabolic dysfunction–associated steatotic liver disease.

Supplementary data 2: Baseline characteristics of patients treated with atezolizumab-bevacizumab before propensity score matching according to the center (n=814)

Baseline characteristics		N	Overall N = 814	Avicenne N = 140	Beaujon N = 169	Sud Francilien N = 6	CHU Caen N = 105	Henri Mondor N = 109	Pitié Salpêtrière N = 125	Saint Antoine N = 64	Toulouse N = 96	p-value
	Elderly patient	814	248 (30%)	35 (25%)	46 (27%)	5 (83%)	60 (57%)	32 (29%)	35 (28%)	11 (17%)	24 (25%)	>0.999
	Gender (male)	814	698 (86%)	115 (82%)	149 (88%)	6 (100%)	87 (83%)	98 (90%)	105 (84%)	59 (92%)	79 (82%)	>0.999
	Obesity	810	172 (21%)	26 (19%)	30 (18%)	1 (17%)	33 (31%)	16 (15%)	25 (20%)	11 (17%)	30 (31%)	>0.999
	Type 2 diabetes	814	336 (41%)	51 (36%)	64 (38%)	5 (83%)	41 (39%)	52 (48%)	52 (42%)	28 (44%)	43 (45%)	>0.999
	Arterial hypertension	814	490 (60%)	86 (61%)	91 (54%)	6 (100%)	45 (65%)	66 (61%)	74 (59%)	38 (59%)	61 (64%)	>0.999
	Dyslipidemia	814	215 (26%)	36 (26%)	38 (22%)	5 (83%)	31 (30%)	38 (35%)	30 (24%)	13 (20%)	24 (25%)	>0.999
	Anticoagulation	794	136 (17%)	23 (16%)	27 (17%)	2 (33%)	15 (14%)	19 (19%)	27 (22%)	7 (11%)	16 (17%)	>0.999
	Cirrhosis	813	605 (74%)	100 (72%)	112 (66%)	5 (83%)	70 (67%)	83 (76%)	107 (86%)	53 (83%)	75 (78%)	>0.999
	ECOG 0-1<br>ECOG 2-3	700	644 (93%)<br>56 (7%)	125 (89%)<br>8 (11%)	141 (83%)<br>16 (17%)	4 (67%)<br>2 (3%)	100 (95%)<br>5 (5%)	65 (87%)<br>10 (13%)	117 (94%)<br>8 (6%)	3 (100%)<br>0 (0%)	89 (93%)<br>7 (7%)	>0.999
Etiologies of liver disease	At least ALD	814	326 (40%)	48 (34%)	49 (29%)	3 (50%)	42 (40%)	54 (50%)	54 (43%)	24 (38%)	52 (54%)	>0.999
	At least MASLD	814	339 (42%)	62 (44%)	57 (34%)	3 (50%)	39 (37%)	42 (39%)	62 (50%)	31 (48%)	43 (45%)	>0.999
	At least viral	814	324 (40%)	66 (47%)	66 (39%)	0 (0%)	27 (26%)	46 (42%)	55 (44%)	31 (48%)	33 (34%)	>0.999
	Mixed etiologies	814	366 (45%)	105 (75%)	36 (21%)	3 (50%)	20 (19%)	38 (35%)	98 (78%)	24 (38%)	42 (44%)	>0.999
Liver function	Previous cirrhosis decompensation	798	159 (20%)	21 (15%)	23 (14%)	1 (17%)	16 (15%)	17 (17%)	40 (32%)	13 (20%)	28 (29%)	>0.999
	Child-Pugh A	807	671 (83%)	97 (71%)	147 (89%)	5 (83%)	95 (90%)	87 (80%)	99 (79%)	50 (79%)	91 (95%)	>0.999
	Child-Pugh B		132 (16%)	39 (28%)	19 (11%)	1 (17%)	10 (10%)	22 (20%)	23 (18%)	13 (21%)	5 (5.2%)	
	Child-Pugh C		4 (0.5%)	1 (0.7%)	0 (0%)	0 (0%)	0 (0%)	0 (0%)	3 (2.4%)	0 (0%)	0 (0%)	
	mALBI grade 1	787	227 (29%)	34 (25%)	56 (33%)	4 (67%)	38 (36%)	23 (21%)	35 (28%)	14 (22%)	23 (30%)	>0.999
	mALBI grade 2a		182 (23%)	28 (20%)	31 (18%)	1 (17%)	25 (24%)	32 (30%)	30 (24%)	17 (27%)	18 (24%)	
	mALBI grade 2b		322 (41%)	62 (45%)	67 (40%)	0 (0%)	38 (36%)	48 (44%)	47 (38%)	29 (46%)	31 (41%)	
	mALBI grade 3		56 (7.1%)	13 (9.5%)	14 (8.3%)	1 (17%)	4 (3.8%)	5 (4.6%)	12 (9.7%)	3 (4.8%)	4 (5.3%)	
	No EV	770	416 (54%)	70 (53%)	71 (47%)	3 (50%)	72 (63%)	55 (56%)	62 (50%)	28 (47%)	55 (57%)	>0.999
	EV (regardless the size)		354 (46%)	62 (47%)	80 (53%)	3 (50%)	33 (37%)	43 (44%)	61 (50%)	31 (53%)	41 (43%)	>0.999
	Large size EV		177 (23%)	32 (25%)	45 (30%)	2 (33%)	8 (13%)	21 (12%)	30 (24%)	14 (23%)	22 (23%)	>0.999
	Creatinine (µmol/l)°	793	73.0 (62.0, 91.0)	72.0 (60.0, 96.5)	74.0 (62.0, 90.0)	81.0 (66.0, 123.0)	72.0 (59.3, 88.0)	76.0 (65.0, 92.0)	74.5 (64.0, 89.5)	72.0 (60.0, 91.0)	72.5 (61.0, 93.0)	0.646
	Total bilirubin (µmol/l)°	793	13.4 (9.0, 21.0)	15.0 (9.0, 21.5)	13.5 (9.0, 19.0)	13.8 (11.0, 19.0)	15.0 (10.0, 21.8)	13.8 (9.0, 23.7)	14.0 (9.0, 23.0)	13.0 (9.0, 23.0)	12.0 (9.5, 17.6)	0.723
	Albumin (g/L)°	791	36.0 (32.0, 39.0)	34.5 (31.0, 39.0)	35.2 (30.0, 40.0)	38.5 (37.0, 40.8)	37.0 (33.0, 40.0)	35.0 (32.0, 38.0)	36.0 (32.0, 39.0)	35.0 (31.0, 38.0)	36.5 (32.0, 39.0)	0.074
	INR°	756	1.1 (1.0, 1.2)	1.1 (1.1, 1.3)	1.1 (1.0, 1.2)	1.1 (1.0, 1.1)	1.1 (1.0, 1.3)	1.2 (1.1, 1.3)	1.1 (1.0, 1.3)	1.2 (1.1, 1.3)	1.0 (1.0, 1.1)	>0.999
HCC features	Previous HCC treatment	775	454 (59%)	77 (55%)	95 (56%)	1 (17%)	64 (93%)	54 (50%)	61 (49%)	44 (71%)	58 (60%)	>0.999
	BCLC-A	813	6 (0.7%)	1 (0.7%)	0 (0%)	0 (0%)	0 (0%)	3 (2.8%)	1 (0.8%)	0 (0%)	1 (1.0%)	>0.999
	BCLC-B		253 (31%)	44 (31%)	63 (37%)	0 (0%)	11 (10%)	32 (29%)	47 (38%)	28 (44%)	28 (29%)	
	BCLC-C		554 (68%)	95 (68%)	106 (63%)	5 (100%)	94 (90%)	74 (68%)	77 (62%)	36 (56%)	67 (70%)	
	AFP (ng/mL)°	786	76.0 (6.7, 1,763)	174.5 (13.0, 3,511)	115.0 (6.7, 2,940)	12.0 (4.4, 458.8)	25.7 (5.6, 251.5)	79.0 (9.1, 1,348.0)	118.0 (7.0, 1,804.0)	43.0 (6.0, 950.0)	12.8 (4.0, 975.0)	0.026
	AFP > 20 ng/mL	786	480 (61%)	98 (71%)	112 (66%)	2 (33%)	52 (50%)	70 (64%)	75 (60%)	33 (53%)	38 (45%)	>0.999
	AFP > 400 ng/mL	786	280 (36%)	57 (41%)	72 (43%)	2 (33%)	23 (22%)	36 (33%)	48 (38%)	18 (29%)	24 (29%)	>0.999
	> 3 lesions	800	443 (55%)	86 (62%)	87 (53%)	4 (67%)	49 (47%)	53 (51%)	58 (48%)	53 (83%)	53 (55%)	>0.999
	Tumor size > 5cm	743	399 (54%)	80 (60%)	92 (60%)	5 (100%)	32 (38%)	57 (56%)	68 (57%)	28 (47%)	37 (46%)	>0.999
	Extrahepatic lesions	814	249 (31%)	33 (24%)	50 (30%)	1 (17%)	40 (38%)	37 (34%)	32 (26%)	18 (28%)	38 (40%)	>0.999
	Vascular invasion	814	301 (37%)	54 (39%)	71 (42%)	4 (67%)	17 (16%)	46 (42%)	49 (39%)	20 (31%)	40 (42%)	>0.999

°median (IQR)

ALD : alcoholic-liver disease; AFP: alpha-fetoprotein; BCLC: Barcelona Clinic Liver Cancer; EV: esophageal varices; HCC : hepatocellular carcinoma ; INR: international normalized ratio; mALBI grade : modified Albumin-Bilirubin grade; MASLD : metabolic dysfunction-associated steatotic liver disease

Supplementary data 3: Flow chart of patients' selection

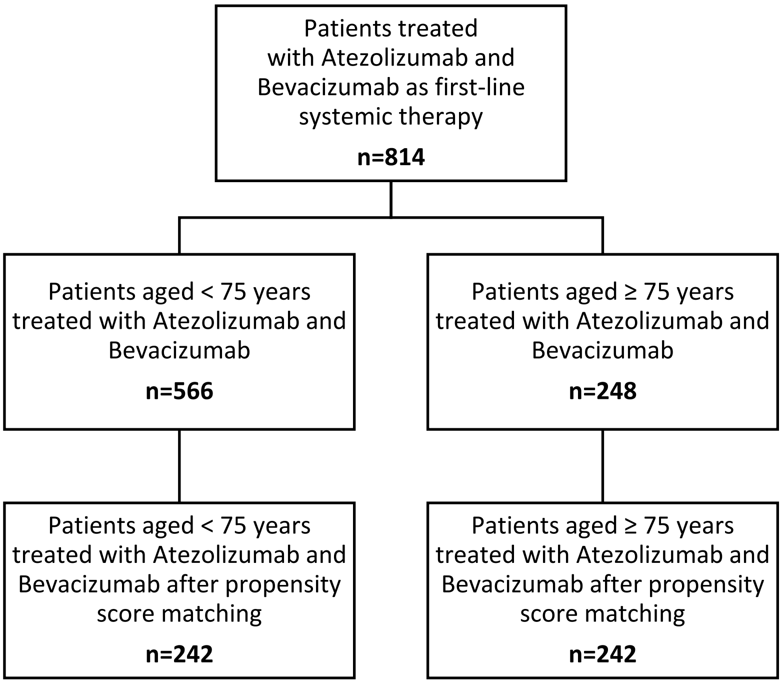

Supplementary data 4: Baseline characteristics of patients treated with atezolizumab-bevacizumab before propensity score matching (n=814)

**Baseline characteristics**		**Available data**	**Whole cohort**	**Available data**	**Elderly patients n=248**	**Available data**	**Non-elderly patients n=566**	**p-value**
Gender (male)		814	698 (86%)	248	214 (86%)	566	484 (86%)	0.770
Body mass index (Kg/m^2) °		809	25.8 (22.8-29.4)	244	26.1 (23.8,29.1)	565	25.6 (22.4,29.8)	0.228
Obesity		810	172 (21%)	244	44 (18%)	566	128 (23%)	0.133
Type 2 diabetes		814	336 (411%)	248	117 (48%)	566	104 (43%)	0.236
Arterial hypertension		814	490 (605)	248	185 (75%)	566	305 (54%)	**<0.001**
Dyslipidemia		814	215 (26%)	248	90 (36%)	566	125 (22%)	**<0.001**
Anticoagulation		794	136 (17%)	242	55 (23%)	552	81 (15%)	**0.006**
Cirrhosis		813	605 (75%)	248	162 (65%)	566	443 (78%)	**<0.001**
ECOG 0-1		700	644 (92%)	229	203 (89%)	471	26 (11%)	**0.023**
ECOG 2-3			56 (8.0%)		26 (11%)		30 (6.4%)	
**Etiologies of liver disease**	At least ALD	813	96 (125)	248	110 (44%)	566	216 (38%)	0.097
	At least MASLD	814	110 (14%)	248	111 (45%)	566	228 (40%)	0.233
	At least viral	813	148 (188)	248	54 (22%)	566	270 (48%)	**<0.001**
	Mixed etiologies	814	366 (45%)	248	99 (40%)	566	267 (47)	0.056
**Liver function Biology**	Previous cirrhosis decompensation	798	159 (20%)	242	28 (12%)	556	131 (24%)	**<0.001**
	MELD score	775	8 (7.0-11.0)	237	8 (7,10)	538	8 (7,11)	0.641
	Child-Pugh A	807	671 (83%)	247	220 (90%)	560	451 (80%)	**0.002**
	Child-Pugh B		132 (16%)		25 (10%)		107 (19%)	
	Child-Pugh C		4 (0.5%)		0 (0%)		4 (1%)	
	mALBI grade 1<br>mALBI grade 2a<br>mALBI grade 2b<br>mALBI grade 3	787	227 (29%)<br>182 (23%)<br>322 (41%)<br>56 (7.1%)	239	70 (29%)<br>76 (32%)<br>85 (36%)<br>8 (3.3%)	548	157 (29%)<br>106 (19%)<br>237 (43%)<br>48 (8.8%)	**<0.001**
	No EV	770	416 (54%)	231	141 (61%)	539	275 (51%)	0.033
	EV (regardless the size)	770	354 (46%)		90 (39%)		264 (49%)	
	Large size EV	770	177 (22.7%)		42 (18%)		135 (25%)	0.317
	Platelet count (x10^3/mm^3)°	795	171 (117, 250)	246	179 (125, 250)	549	166 (115,255)	0.283
	Creatinine (µmol/l)°	793	73 (62-91)	241	81 (66, 103)	552	71 (60,86)	**<0.001**
	Total bilirubin (µmol/l)°	793	13.4 (9-21)	241	12 (9, 18)	552	14 (10, 22)	**<0.001**
	Albumin (g/L)°	791	36 (32-39)	239	36 (33, 39)	552	35 (31, 39)	**0.012**
	INR°	756	1.1 (1.0-1.2)	233	1.1 (1.0, 1.2)	523	1.1 (1.0, 1.3)	**<0.001**
**HCC features**	Previous HCC treatment	775	454 (59%)	226	148 (65%)	549	306 (56%)	**0.012**
	BCLC-A	813	6 (0.7%)	247	1(1%)	566	5(1%)	0.552
	BCLC-B		253 (31%)		83 (33%)		170 (30%)	
	BCLC-C		554 (68%)		163 (66%)		391 (69%)	
	AFP (ng/mL)°	786	76 (6.7-1763)	235	54 (5,919)	551	95 (9,2500)	**0.010**
	AFP>20 ng/mL	786	480 (61%)	235	163 (69%)	551	384 (70%)	0.933
	AFP>400 ng/mL	786	333 (42%)	235	85 (86%)	551	209 (38%)	0.687
	>3 lesions	800	443 (55%)	247	132 (53%)	553	311 (56%)	0.462
	Size of the largest lesion (mm)°	743	52 (26-90)	229	51 (24, 80)	514	54 (28,100)	0.172
	Tumor size > 5cm	743	399 (54%)	229	120 (52%)	514	279 (54%)	0.635
	Extrahepatic lesions	814	249 (31%)	248	76 (31%)	566	173 (31%)	0.982
	Vascular invasion	814	301 (37%)	248	66 (27%)	566	235 (42%)	**<0.001**
**AtezoBev treatment**	Treatment duration (months)°	814	4.7 (1.9-11.3)	248	4.9 (1.5,11.7)	566	4.5 (2.0,10.9)	0.927
	Second-line treatment	695	229 (33%)	210	65 (31%)	485	164 (31%)	0.427

°median (IQR)

ALD : alcoholic-liver disease; AtezoBev : Atezolizumab-Bevacizumab; AFP: alpha-fetoprotein; BCLC: Barcelona Clinic Liver Cancer; EV: esophageal varices; HCC : hepatocellular carcinoma ; INR: international normalized ratio; mALBI grade : modified Albumin-Bilirubin grade; MASLD : metabolic dysfunction-associated steatotic liver disease; MELD score : Model for End-Stage Liver Disease

Supplementary data 5: Overall Survival and Progression-Free Survival in the whole cohort of patients (n=814)

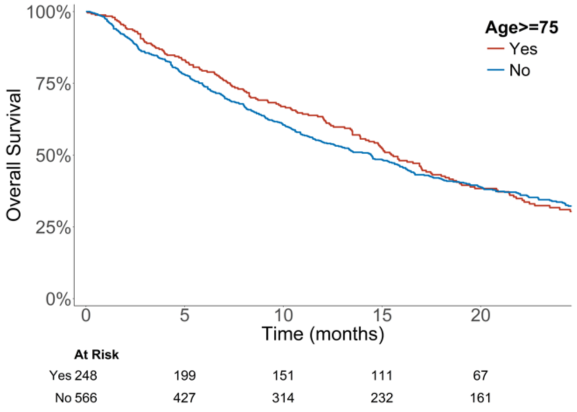

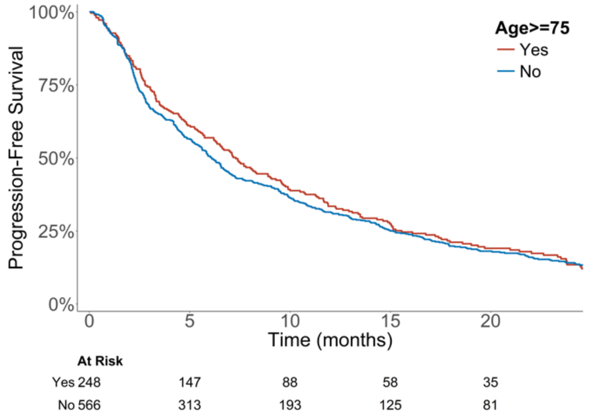

P=0.486

P=0.372

Supplementary data 6: Baseline predictive factors of progression free survival in the propensity-score matched population regardless of age (n=484) (Cox proportional hazard regression models)

	Available data (n=484)	Univariate analysis			Multivariate analysis		
		**HR**	**95 CI**	**p value**	**HR**	**95 CI**	**p value**
Age ≥ 75 years		0.96	0.79, 1.17	0.706			
Gender (male)	484	0.77	0.59, 1.01	**0.050**	0.81	0.60,1.09	0.163
Obesity	481	0.86	0.67, 1.09	0.209			
Type 2 diabetes	484	0.83	0.68, 1.01	0.060			
Arterial hypertension	484	0.82	0.67, 1.01	0.061			
Dyslipidemia	484	1.04	0.85, 1.28	0.677			
Anticoagulation	470	0.91	0.70, 1.18	0.490			
Cirrhosis	483	0.92	0.74, 1.13	0.431			
ECOG 0-1<br>ECOG 2-3	424	-<br>1.68	-<br>1.19, 2.37	-<br>**0.003**	-<br>1.42	-<br>0.97-2.08	-<br>0.068
At least ALD	484	0.99	0.82, 1.21	0.936			
At least MASLD	484	0.89	0.74, 1.09	0.263			
At least viral	484	1.07	0.86, 1.34	0.527			
Mixed etiologies	484	0.97	0.80, 1.19	0.795			
Esophageal varices	456	1.09	0.89, 1.34	0.385			
Large size EV	456	1.04	0.93, 1.16	0.514			
Previous cirrhosis decompensation	473	1.36	1.00, 1.84	**0.048**	1.13	0.80, 1.62	0.486
Platelet count	471	1.00	1.00, 1.00	**0.018**			
Albumin°	469	0.95	0.93, 0.97	**<0.001**			
Total bilirubin°	469	1.01	1.01, 1.02	**0.001**			
Creatinine	469	1.00	1.00, 1.00	0.430			
INR	449	1.34	0.80, 2.25	0.267			
MELD Score°	458	1.01	0.98, 1.05	0.360			
Child-Pugh C°<br>Child-Pugh B<br>Child-Pugh A	479	-<br>1.57<br>0.62	-<br>1.08, 2.27<br>0.43, 0.89	-<br>**0.017**<br>**0.011**			
mALBI grade 1<br>mALBI grade2a<br>mALBI grade2b<br>mALBI grade3	465	-<br>1.20<br>1.63<br>3.74	-<br>0.92, 1.56<br>1.29, 2.07<br>2.21, 6.34	-<br>0.182<br>**<0.001**<br>**<0.001**	-<br>1.27<br>1.59<br>3.77	-<br>0.94, 1.72<br>1.22, 2.06<br>2.12,6.70	**-**<br>0.123<br>**<0.001**<br>**<0.001**
Previous HCC treatment	455	1.07	0.85, 1.35	0.571			
BCLC-A<br>BCLC-B<br>BCLC-C	483	-<br>0.53<br>0.61	-<br>0.21, 1.28<br>0.25, 1.48	-<br>0.158<br>0.275			
AFP (ng/mL)	463	1.00	1.00, 1.00	**0.012**			
AFP >20 (ng/mL)	463	1.46	1.19, 1.79	**<0.001**			
AFP > 400 ng/mL	463	1.50	1.22, 1.86	**<0.001**	1.48	1.16, 1.88	**0.002**
>3 lesions	479	1.03	0.85, 1.26	0.741			
Size of the largest lesion (mm)	437	1.00	1.00, 1.00	0.746			
Tumor size > 5cm	437	1.04	0.85, 1.28	0.678			
Extrahepatic lesions	484	1.24	1.00, 1.53	**0.046**	1.48	1.17, 1.86	**0.001**
Vascular invasion	484	0.95	0.77, 1.19	0.675			

* ECOG performance status was not included in the multivariate analysis because the proportion of missing values exceeded the predefined 10% threshold for imputation.

° Child-Pugh score, MELD score, albumin and bilirubin were not entered in the multivariate analysis in order to avoid collinearity with ALBI grade 3 classification.

ALD : alcoholic-liver disease; AtezoBev : Atezolizumab-Bevacizumab; AFP: alpha-fetoprotein; BCLC: Barcelona Clinic Liver Cancer; EV: esophageal varices; HCC : hepatocellular carcinoma ; INR: international normalized ratio; mALBI grade : modified Albumin-Bilirubin grade; MASLD : metabolic dysfunction-associated steatotic liver disease; MELD score : Model for End-Stage Liver Disease

Supplementary data 7: Baseline predictive factors of mortality in propensity-score matched population regardless of age (n=484) (Cox proportional hazard regression models)

	Available data (n=484)	Univariate analysis			Multivariate analysis		
		**HR**	**95 CI**	**p value**	**HR**	**95 CI**	**p value**
Age ≥ 75 years	484	0.99	0.80-1.23	0.937			
Gender (male)	484	1.00	0.73, 1.35	0.980			
Obesity	481	0.86	0.65, 1.13	0.274			
Type 2 diabetes	484	0.90	0.73, 1.12	0.362			
Arterial hypertension	484	0.95	0.76, 1.19	0.655			
Dyslipidemia	484	1.23	0.97, 1.54	0.083			
Anticoagulation	470	1.01	0.75, 1.35	0.973			
Cirrhosis	483	0.98	0.78, 1.24	0.890			
ECOG 0-1<br>ECOG 2-3	424	-<br>1.89	-<br>1.30, 2.75	-<br>**<0.001**	-<br>1.56	-<br>1.01-2.42	-<br>**0.046**
At least ALD	484	1.30	0.96, 1.77	0.095			
At least MASLD	484	1.01	0.76, 1.33	0.954			
At least viral	484	0.81	0.57, 1.16	0.250			
Mixed etiologies	484	1.00	0.80, 1.25	0.983			
Esophageal varices	456	1.26	1.00, 1.58	**0.045**	1.02	0.77, 1.36	0.867
Large size EV	456	1.13	1.00, 1.28	0.051			
Previous cirrhosis decompensation	473	1.33	0.92, 1.92	0.126			
Platelet count	471	1.00	1.00, 1.00	0.073			
Albumin°	469	0.94	0.92, 0.96	**<0.001**			
Total bilirubin°	469	1.02	1.01, 1.03	**<0.001**			
Creatinine	469	1.00	1.00, 1.00	0.953			
INR	449	1.64	0.99-2.74	0.056			
MELD Score°	458	1.04	1.01, 1.07	**0.014**			
Child-Pugh C°<br>Child-Pugh B<br>Child-Pugh A	479	-<br>2.02<br>0.48	-<br>1.44, 2.85<br>0.34, 0.67	-<br>**<0.001**<br>**<0.001**			
mALBI grade 1<br>mALBI grade2a<br>mALBI grade2b<br>mALBI grade3	465	-<br>1.46<br>1.99<br>5.10	-<br>1.08, 1.97<br>1.52, 2.61<br>2.88, 9.03	-<br>**0.014**<br>**<0.001**<br>**<0.001**	-<br>1.45<br>1.60<br>4.58	-<br>0.99, 2.12<br>1.13, 2.26<br>2.36, 8.88	-<br>0.057<br>**0.007**<br>**<0.001**
Previous HCC treatment	455	0.71	0.57, 0.90	**0.004**	0.98	0.71, 1.35	0.904
BCLC-A<br>BCLC-B<br>BCLC-C	483	-<br>1.15<br>1.54	-<br>0.36, 3.64<br>0.49, 4.83	-<br>0.812<br>0.459			
AFP (ng/mL)	463	1.00	1.00, 1.00	**<0.001**			
AFP> 20 ng/mL		1.46	1.20, 1.91	**0.001**			
AFP > 400 ng/mL	463	1.51	1.20, 1.93	**<0.001**	1.46	1.09, 1.96	**0.011**
>3 lesions	479	1.08	0.87, 1.34	0.500			
Size of the largest lesion (mm)	437	1.00	1.00, 1.00	**0.017**			
Tumor size > 5cm	437	1.37	1.09, 1.73	**0.007**	1.08	0.79, 1.48	0.636
Extrahepatic lesions	484	1.17	0.92, 1.48	0.201			
Vascular invasion	484	1.25	0.98, 1.58	0.069			

* ECOG performance status was not included in the multivariate analysis because the proportion of missing values exceeded the predefined 10% threshold for imputation.

° Child-Pugh score, MELD score, albumin and bilirubin were not entered in the multivariate analysis in order to avoid collinearity with ALBI grade 3 classification.

ALD : alcoholic-liver disease; AtezoBev : Atezolizumab-Bevacizumab; AFP: alpha-fetoprotein; BCLC: Barcelona Clinic Liver Cancer; EV: esophageal varices; HCC : hepatocellular carcinoma ; INR: international normalized ratio; mALBI grade : modified Albumin-Bilirubin grade; MASLD : metabolic dysfunction-associated steatotic liver disease; MELD score : Model for End-Stage Liver Disease

Supplementary data 8: Baseline characteristics of patients with an mALBI score of 3 treated with atezolizumab-bevacizumab (n=16)

Baseline characteristics		Available data	Non-elderly patients (< 75 years) n=8	Elderly patients (≥ 75 years) n=8	p-value
Age (years)		16	57.5 (53.7, 61.7)	78.5 (76.0, 80.5)	**<0.001**
Gender (male)		16	5 (63%)	6 (75%)	>0.999
Body mass index (Kg/m^2) °		16	23.9 (18.2, 24.8)	24.8 (23.5, 26.5)	0.328
Obesity		16	0 (0%)	0 (0%)	**0.328**
Type 2 diabetes		16	2 (25%)	5 (63%)	0.315
Arterial hypertension		16	2 (25%)	5 (63%)	0.315
Dyslipidemia		16	0 (0%)	5 (63%)	**0.026**
Anticoagulation		16	0 (0%)	1 (13%)	>0.999
Cirrhosis		16	5 (63%)	6 (75%)	>0.999
ECOG 0-1<br>ECOG 2-3		16	5 (63%)<br>3 (38%)	8 (100%)<br>0 (0%)	0.200
**Etiologies of liver disease**	At least ALD	16	1 (13%)	3 (38%)	0.569
	At least MASLD	16	3 (38%)	7 (88%)	0.119
	At least viral	16	2 (25%)	0 (0%)	0.467
	Mixed etiologies	16	3 (38%)	4 (50%)	>0.999
**Liver function**	Previous cirrhosis decompensation	16	3 (38%)	3 (38%)	>0.999
	Child-Pugh A	16	2 (25%)	1 (13%)	0.259
	Child-Pugh B		5 (63%)	7 (87%)	
	Child-Pugh C		3 (12%)	0 (0%)	
	No EV	16	4 (50%)	2 (25%)	0.608
	EV (regardless the size)		4 (50%)	6 (75%)	
	Large size EV		3 (37%)	2 (25%)	0.277
	Platelet count (x10^3/mm^3)°	16	188 (128, 277)	124 [(98, 265)	0.442
	Creatinine (µmol/l)°	16	60.0 (54.5, 72.0)	66.5 (51.9, 78.0)	0.721
	Total bilirubin (µmol/l)°	16	31.7 (15.0, 69.0)	31.1 (25.2, 37.7)	>0.999
	INR°	16	1.2 (1.1, 1.4)	1.4 (1.2, 1.5)	0.247
**HCC features**	Previous HCC treatment	14	4 (50%)	2 (33%)	0.627
	BCLC-B	16	4 (50%)	1 (13%)	0.282
	BCLC-C		4 (50%)	7 (88%)	
	AFP (ng/mL)°	16	414.0 (5.6, 13,897.5)	63.5([4.5, 1,014.4)	0.574
	AFP > 20 ng/mL	16	5 (63%)	4 (50%)	>0.999
	AFP > 400 ng/mL	16	4 (50%)	3 (38%)	>0.999
	> 3 lesions	16	4 (50%)	4 (50%)	>0.999
	Size of the largest lesion (mm)°	16	100.0 (80.0, 105.0)	59.5 (33.5, 110.0)	0.114
	Tumor size > 5cm	16	8 (100%)	5 (63%)	0.200
	Extrahepatic lesions	16	2 (25%)	1 (13%)	>0.999
	Vascular invasion	16	2 (25%)	3 (38%)	>0.999
**AtezoBev treatment**	Treatment duration (months)°	16	1.4 (0.7, 3.7)	1.4 (0.9, 2.2)	>0.999
	Second-line treatment	16	4 (50%)	2 (25%)	0.608

°median (IQR)

ALD : alcoholic-liver disease; AtezoBev : Atezolizumab-Bevacizumab; AFP: alpha-fetoprotein; BCLC: Barcelona Clinic Liver Cancer; EV: esophageal varices; HCC : hepatocellular carcinoma ; INR: international normalized ratio; mALBI grade : modified Albumin-Bilirubin grade; MASLD : metabolic dysfunction-associated steatotic liver disease

Supplementary data 9: Comparison between patients according to the occurrence of hypertension during treatment

Baseline characteristics		Elderly patients n=223		p-value
		**Yes n=39**	**No n=184**	
Gender (male)		32 (82%)	160 (87%)	0.421
Body mass index (Kg/m²) °		27 (24, 30)	26 (24, 29)	0.129
Obesity		10 (26%)	29 (16%)	0.154
Type 2 diabetes		23 (59%)	85 (46%)	0.147
Arterial hypertension		31 (79%)	133 (72%)	0.354
Dyslipidemia		18 (46%)	64 (35%)	0.181
Anticoagulation		8 (21%)	40 (22%)	0.853
Cirrhosis		25 (64%)	120 (65%)	0.895
**Performance Status (ECOG)**	0	18 (46%)	59 (33%)	0.452
	1	19 (49%)	97 (54%)	
	2	2 (5%)	18 (10%)	
	3	0 (0%)	4 (2%)	
**Etiologies of liver disease**	At least ALD	15 (38%)	78 (42%)	0.651
	At least MASLD	21 (54%)	80 (43%)	0.237
	At least viral	9 (23%)	41 (22%)	0.914
	Mixed etiologies	13 (33%)	75 (41%)	0.389
**History of decompensated cirrhosis**	Previous cirrhosis decompensation	6 (15%)	17 (9.3%)	0.254
	Previous hepatic encephalopathy	1 (2.6%)	10 (5.4%)	0.694
	Previous variceal bleeding	0 (0%)	6 (3.3%)	0.593
	Previous ascitic decompensation	5 (13%)	13 (7.1%)	0.327
	Previous ascitic infection	1 (2.6%)	3 (1.6%)	0.539
	Beta-blocker therapy	7 (18%)	50 (27%)	0.230
**Liver function**	Child-Pugh A	38 (97%)	159 (88%)	0.087
	Child-Pugh B	1 (2.6%)	22 (12%)	0.087
	mALBI grade 1	19 (53%)	62 (35%)	**0.043**
	mALBI grade 2-3	17 (47%)	116 (65%)	
	MELD score	8 (7, 11)	8 (7, 10)	0.844
	No EV	27 (68%)	117 (61%)	0.428
	EV (regardless the size)	12 (32%)	67 (39%)	
	Large size EV	6 (16%)	31 (18%)	0.762
	Platelet count (x10³/mm³)°	176 (121, 210)	185 (130, 272)	0.098
**Biology**	Creatinine (µmol/l)°	81 (64, 96)	83 (67, 103)	0.318
	Total bilirubin (µmol/l)°	12 (10, 16)	12 (9, 19)	0.951
	Albumin (g/L)°	38 (36, 41)	36 (33, 39)	**0.009**
	INR°	1.1 (1.0, 1.2)	1.1 (1.0, 1.2)	0.316
**HCC features**	Active monitoring of cirrhosis	12 (31%)	60 (33%)	0.807
	Previous HCC treatment	24 (73%)	106 (63%)	0.290
	BCLC-A	0 (0%)	1 (0.5%)	0.640
	BCLC-B	10 (26%)	60 (33%)	
	BCLC-C	28 (74%)	123 (67%)	
	AFP (ng/mL)°	30 (5,875)	59 (5,1065)	0.535
	Monofocal	5 (13%)	55 (30%)	**0.028**
	Size of the largest lesion (mm)°	56 (29,88)	50 (23,80)	0.421
	Tumor size > 5cm	20 (59%)	88 (52%)	0.452
	Extrahepatic lesions	13 (33%)	60 (33%)	0.930
	Vascular invasion	7 (18%)	51 (28%)	0.207
**AtezoBev treatment**	Treatment duration (months)°	8.3 (2.8,14.8)	4.1 (1.4,9.7)	**0.009**
	Second-line treatment	11 (31%)	51 (31%)	0.917

°median (IQR)

ALD : alcoholic-liver disease; AtezoBev : Atezolizumab-Bevacizumab; AFP: alpha-fetoprotein; BCLC: Barcelona Clinic Liver Cancer; EV: esophageal varices; HCC : hepatocellular carcinoma ; INR: international normalized ratio; mALBI grade : modified Albumin-Bilirubin grade; MASLD : metabolic dysfunction-associated steatotic liver disease; MELD score : Model for End-Stage Liver Disease

Supplementary data 10: Hypertension occurrence according to hypertension (HTN) prior history. This alluvial plot illustrates the occurrence of arterial hypertension during treatment, defined according to CTCAE criteria, stratified by hypertension status at baseline. The left side of the plot represents the presence or absence of hypertension at baseline, while the right side represents the development of on-treatment arterial hypertension.

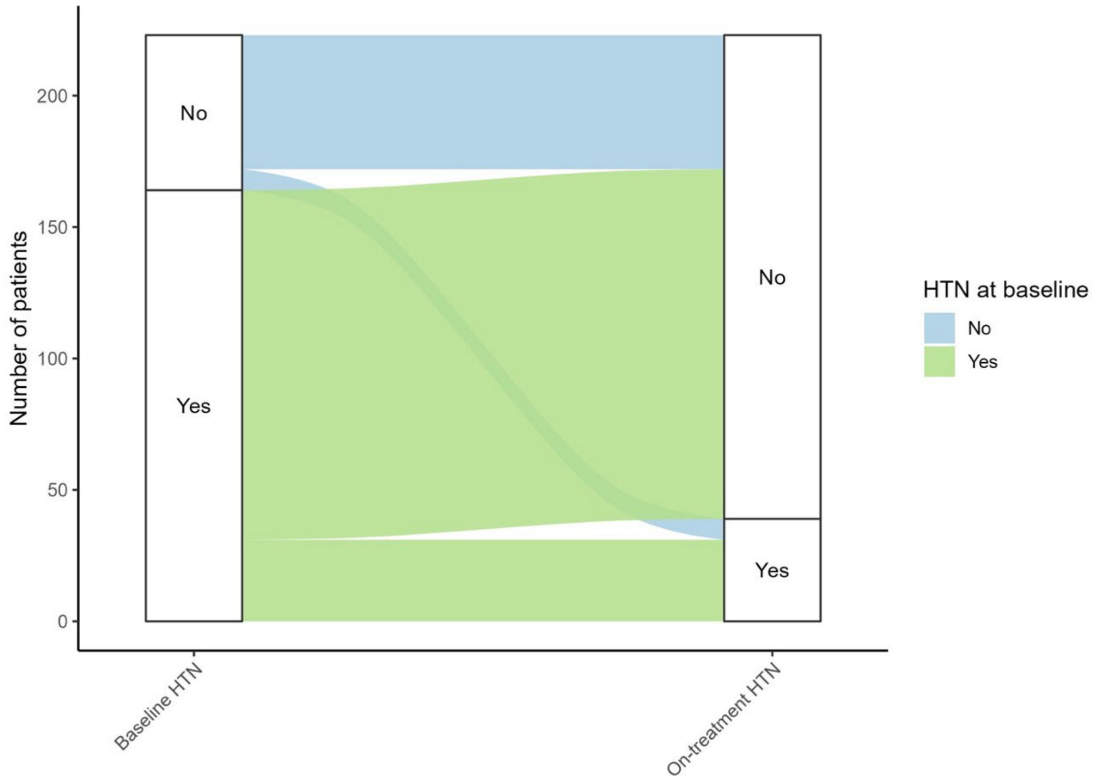

Supplementary data 11. Comparison between patients according to the occurrence of proteinuria during treatment

Baseline characteristics		Elderly patients n=223		p-value
		Yes n=20	No n=203	
Gender (male)		16 (80%)	176 (87%)	0.494
Body mass index (Kg/m^2) °		26 (24, 29)	26 (24, 29)	0.902
Obesity		4 (20%)	35 (18%)	0.761
Type 2 diabetes		13 (65%)	95 (47%)	0.120
Arterial hypertension		17 (85%)	147 (72%)	0.223
Dyslipidemia		4 (20%)	78 (38%)	0.103
Anticoagulation		2 (10%)	46 (23%)	0.259
Cirrhosis		12 (60%)	133 (66%)	0.622
**Performance Status (ECOG)**	0	9 (45%)	68 (35%)	0.756
	1	9 (45%)	107 (54%)	
	2	2 (10%)	18 (9.1%)	
	3	0 (0%)	4 (2.0%)	
**Etiologies of liver disease**	At least ALD	9 (45%)	84 (41%)	0.754
	At least MASLD	11 (55%)	90 (44%)	0.361
	At least viral	7 (35%)	43 (21%)	0.166
	Mixed etiologies	8 (40%)	80 (39%)	0.959
**History of decompensated cirrhosis**	Previous cirrhosis decompensation	1 (5.0%)	22 (11%)	0.702
	Previous hepatic encephalopathy	0 (0%)	11 (5.4%)	0.605
	Previous variceal bleeding	0 (0%)	6 (3.0%)	>0.999
	Previous ascitic decompensation	0 (0%)	18 (8.9%)	0.381
	Previous ascitic infection	1 (5.0%)	3 (1.5%)	0.315
	Beta-blocker therapy	2 (10%)	55 (27%)	0.095
**Liver function**	Child-Pugh A	20 (100%)	177 (89%)	0.239
	Child-Pugh B	0 (0%)	23 (12%)	0.239
	mALBI grade 1	11 (55%)	70 (36%)	0.097
	mALBI grade 2-3	9 (45%)	124 (64%)	
	MELD score	8 (6, 10)	8 (7, 10)	0.213
	No EV	15 (72%)	129 (61%)	0.342
	EV (regardless the size)	5 (28%)	74 (39%)	
	Large size EV	0 (0%)	37 (20%)	0.188
	Platelet count (x10^3/mm^3)°	183 (147, 246)	181 (126, 252)	0.763
**Biology**	Creatinine (µmol/l)°	74 (62, 94)	83 (67, 103)	0.342
	Total bilirubin (µmol/l)°	10 (7, 15)	12 (9, 19)	0.103
	Albumin (g/L)°	37 (35, 42)	36 (33, 39)	**0.043**
	INR°	1.1 (1.0, 1.2)	1.1 (1.0, 1.2)	0.316
**HCC features**	Active monitoring of cirrhosis	7 (35%)	65 (32%)	0.797
	Previous HCC treatment	12 (67%)	118 (64%)	0.853
	BCLC-A	0 (0%)	1 (0.5%)	0.378
	BCLC-B	4 (20%)	66 (33%)	
	BCLC-C	16 (80%)	135 (67%)	
	AFP (ng/mL)°	66 (5,394)	58 (5,973)	0.642
	Monofocal	3 (15%)	57 (28%)	0.204
	Size of the largest lesion (mm)°	46 (26,83)	51 (26,83)	0.515
	Tumor size > 5cm	8 (50%)	100 (53%)	0.806
	Extrahepatic lesions	5 (25%)	68 (33%)	0.440
	Vascular invasion	6 (30%)	52 (26%)	0.670
**AtezoBev treatment**	Treatment duration (months)°	13 (5.1,15.2)	4.2 (1.4,10.3)	**0.009**
	Second-line treatment	7 (37%)	55 (30%)	0.541

°median (IQR)

ALD : alcoholic-liver disease; AtezoBev : Atezolizumab-Bevacizumab; AFP: alpha-fetoprotein; BCLC: Barcelona Clinic Liver Cancer; EV: esophageal varices; HCC : hepatocellular carcinoma ; INR: international normalized ratio; mALBI grade : modified Albumin-Bilirubin grade; MASLD : metabolic dysfunction-associated steatotic liver disease; MELD score : Model for End-Stage Liver Disease

Supplementary data 12: Comparison between patients according to the occurrence of immune-related adverse events during treatment

Baseline characteristics		Elderly patients n=226		p-value
		Yes n=50	No n=176	
Gender (male)		48 (96%)	145 (82%)	**0.016**
Body mass index (Kg/m^2) °		26 (24, 29)	26 (24, 29)	0.660
Obesity		9 (18%)	31 (18%)	0.990
Type 2 diabetes		22 (44%)	86 (49%)	0.543
Arterial hypertension		40 (80%)	126 (72%)	0.235
Dyslipidemia		19 (38%)	63 (36%)	0.775
Anticoagulation		15 (30%)	35 (20%)	0.134
Cirrhosis		37 (74%)	110 (63%)	0.132
**Performance Status (ECOG)**	0	20 (41%)	59 (35%)	0.199
	1	27 (55%)	90 (53%)	
	2	1 (2.0%)	19 (11%)	
	3	1 (2.0%)	3 (1.8%)	
**Etiologies of liver disease**	At least ALD	21 (42%)	73 (41%)	0.947
	At least MASLD	18 (36%)	84 (48%)	0.141
	At least viral	16 (32%)	35 (20%)	0.071
	Mixed etiologies	17 (34%)	73 (41%)	0.341
**History of decompensated cirrhosis**	Previous cirrhosis decompensation	17 (9.3%)	6 (15%)	0.254
	Previous hepatic encephalopathy	3 (6.0%)	8 (4.5%)	0.711
	Previous variceal bleeding	2 (4.0%)	4 (2.3%)	0.616
	Previous ascitic decompensation	3 (6.0%)	16 (9.1%)	0.773
	Previous ascitic infection	1 (2.0%)	3 (1.7%)	>0.999
	Beta-blocker therapy	12 (24%)	46 (26%)	0.760
**Liver function**	Child-Pugh A	45 (94%)	156 (89%)	0.424
	Child-Pugh B	3 (6.3%)	19 (11%)	0.424
	mALBI grade 1	26 (55%)	57 (34%)	**0.007**
	mALBI grade 2-3	21 (45%)	113 (66%)	
	MELD score	8 (7, 12)	8 (7, 10)	0.440
	No EV	32 (62%)	112 (61%)	0.905
	EV (regardless the size)	18 (38%)	64 (39%)	
	Large size EV	5 (15%)	30 (19%)	0.545
	Platelet count (x10^3/mm^3)°	168 (137, 222)	188 (126, 267)	0.470
**Biology**	Creatinine (µmol/l)°	85 (66, 99)	80 (66, 103)	0.587
	Total bilirubin (µmol/l)°	14 (8, 18)	12 (9, 18)	0.476
	Albumin (g/L)°	38 (36, 41)	36 (32, 39)	**0.002**
	INR°	1.1 (1.0, 1.2)	1.1 (1.0, 1.2)	0.325
**HCC features**	Active monitoring of cirrhosis	15 (30%)	59 (34%)	0.622
	Previous HCC treatment	32 (65%)	100 (65%)	0.920
	BCLC-A	1 (2.0%)	0 (0%)	0.079
	BCLC-B	19 (38%)	51 (29%)	
	BCLC-C	30 (60%)	124 (71%)	
	AFP (ng/mL)°	37 (5,430)	65 (5,1079)	0.429
	Monofocal	14 (29%)	47 (27%)	0.795
	Size of the largest lesion (mm)°	45 (22,89)	52 (27,80)	0.685
	Tumor size > 5cm	21 (50%)	88 (53%)	0.699
	Extrahepatic lesions	12 (24%)	61 (35%)	0.155
	Vascular invasion	15 (30%)	46 (26%)	0.587
**AtezoBev treatment**	Treatment duration (months)°	10.6 (2.8,19.0)	4.1 (1.5,8.9)	**<0.001**
	Second-line treatment	9 (20%)	53 (33%)	0.090

°median (IQR)

ALD : alcoholic-liver disease; AtezoBev : Atezolizumab-Bevacizumab; AFP: alpha-fetoprotein; BCLC: Barcelona Clinic Liver Cancer; EV: esophageal varices; HCC : hepatocellular carcinoma ; INR: international normalized ratio; mALBI grade : modified Albumin-Bilirubin grade; MASLD : metabolic dysfunction-associated steatotic liver disease; MELD score : Model for End-Stage Liver Disease
