# Supplementary material for: Atezolizumab–bevacizumab in very elderly with hepatocellular carcinoma: Age alone is not a limiting factor except in ALBI grade 3
Source: JHEP Rep. 2026 Mar 17;8(6):101827. doi: 10.1016/j.jhepr.2026.101827 (PMC13195595; doi:10.1016/j.jhepr.2026.101827)
Supplement: Multimedia component 4 [file mmc4.pdf]

JHEP|Reports

Atezolizumab–bevacizumab in very elderly with hepatocellular carcinoma: Age alone is not a limiting factor except in ALBI grade 3

Authors

Chloé Métivier, Claudia Campani, Manon Allaire, …, Charlotte Costentin, Aurore Baron, Isabelle Ollivier Hourmand

Correspondence

metivier-c@chu-caen.fr (C. Métivier).

Graphical abstract

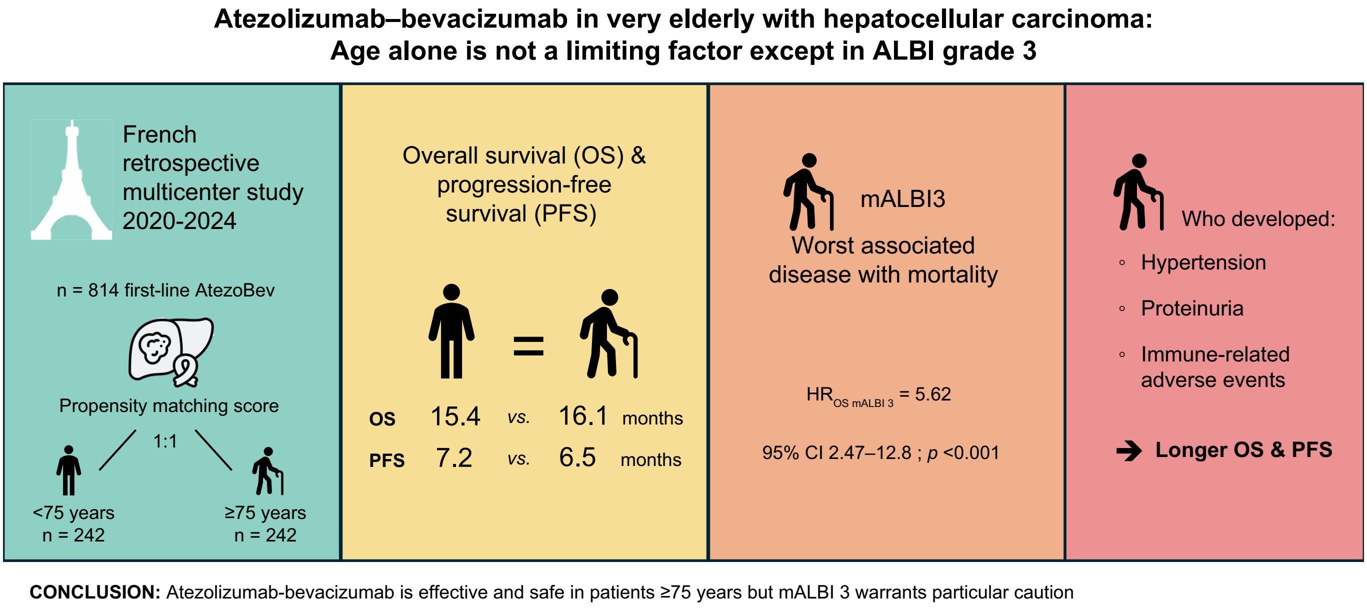

Highlights:

- Patients aged ≥75 years with advanced hepatocellular carcinoma are common.
- Evidence on the safety and efficacy of atezolizumab plus bevacizumab in this age group remains limited.
- Overall survival and progression-free survival appear comparable between patients aged <75 and those ≥75 years.
- Particular caution is warranted in patients aged ≥75 years with mALBI grade 3.

Impact and implications:

Very elderly patients (≥75) are underrepresented in immunotherapy trials, leaving uncertainty about the benefit–risk of atezolizumab plus bevacizumab in this subgroup. In real-world practice, European ancestry patients ≥75 with advanced hepatocellular carcinoma are common. Our study indicates that atezolizumab–bevacizumab is generally safe and effective in this population, with mALBI grade 3 as a notable high-risk exception. Although retrospective, the use of propensity score methods mitigates confounding and supports the robustness of our conclusions.

https://doi.org/10.1016/j.jhepr.2026.101827

© 2026 The Authors. Published by Elsevier B.V. on behalf of European Association for the Study of the Liver (EASL). This is an open access article under the CC BY license (http://creativecommons.org/licenses/by/4.0/).

JHEP|Reports

Atezolizumab–bevacizumab in very elderly with hepatocellular carcinoma: Age alone is not a limiting factor except in ALBI grade 3

Chloé Métivier[1,2,*,†], **Claudia Campani**[3,4,†], Manon Allaire[5], Rémy Morello[2,6], Sarah Mouri[5], Eleonore Spitzer[5], Mohamed Bouattour[7,8], Clémence Hollande[7,8], Sabrina Sidali[7], Jean Charles Nault[3,4], Nathalie Ganne-Carrié[4], Pierre Nahon[4], Giuliana Amaddeo[9], Hélène Regnault[9], Paul Vigneron[9], Jean Marie Péron[10], Leila Sadek[10], Cecile Cussac[10], Marie Lequoy[11], Violaine Ozenne[11], Marie-Pierre Galais[12], Claire Pérignon[1], Louise Lebedel[1], Marion Habireche[1], Apolline Commin[1], Thông Dao[1], Charlotte Costentin[13,14], Aurore Baron[15], Isabelle Ollivier Hourmand[1,2]

JHEP Reports **2026**. vol. 8 | 1–10

Check for updates

Background & Aims: Evidence on atezolizumab plus bevacizumab (AtezoBev) in very elderly people with hepatocellular carcinoma (HCC) remains rare in the European ancestry population. We compared outcomes in patients aged ≥75 years with younger patients.

Methods: This multicenter retrospective study included patients treated with first-line AtezoBev for advanced HCC. Patients aged ≥75 years were matched 1:1 with those <75 years using propensity scores. Overall survival (OS), progression-free survival (PFS), tumor response, and adverse events (AEs) were analyzed.

Results: Among 814 patients, 566 (69.5%) were <75 years (median age 64, 84% male) and 248 (30.5%) ≥75 years (median age 78, 86% male). After matching, 484 were analyzed. After a median follow-up of 28.0 months, OS and PFS were similar in ≥75 and <75 (15.4 *vs*. 16.07 months; *p* = 0.936), (7.2 vs. 6.5 months; *p* = 0.706) respectively. Age ≥ 75 years was not associated with PFS nor OS. In patients aged ≥75 years, modified albumin–bilirubin grade 3 (mALBI) was the only factor associated with disease progression (HR 4.37, 95% CI 2.04,9.37; *p* <0.001) and mortality (HR 5.62, 95% CI 2.47,12.8; *p* <0.001). In mALBI 3 median OS and PFS were 5.43 and 2.3 months, respectively. Immune-related AEs were less frequent in ≥75 including (22.1% *vs.* 36.9%; *p* <0.001) or excluding dysthyroidism (15.9% *vs.* 25.9%; *p* = 0.01). In univariate analysis, OS and PFS were longer in patients ≥75 years who developed hypertension (*p* = 0.04 and *p* = 0.09), proteinuria (*p* <0.0001 and *p* = 0.015) and Immune-related AEs (*p* = 0.02 and *p* = 0.007). Hypertension during treatment was associated with proteinuria (odds ratio = 13.1; 95% CI 4.1–42.4), without difference at baseline (*p* = 0.35).

Conclusions: AtezoBev is effective and safe in patients ≥75 years but mALBI 3 warrants particular caution.

Clinical trials registration number: NCT06416683.

© 2026 The Authors. Published by Elsevier B.V. on behalf of European Association for the Study of the Liver (EASL). This is an open access article under the CC BY license (http://creativecommons.org/licenses/by/4.0/).

Introduction

Primary liver cancer is the sixth most common cancer and the third leading cause of cancer-related death worldwide, with hepatocellular carcinoma (HCC) accounting for 75–85%.[1] Half of patients are diagnosed at an advanced and/or unresectable stage, for which immunotherapy has become the standard first-line systemic therapy since the publication of the IMbrave150 trial in 2020.[2,3] In this study, the median age was 64 years, whereas French national registry reports a median age at HCC diagnosis of 69 years in men and 73 years in women.[4]

Despite immunotherapy widespread adoption, pivotal trials included few elderly patients, and data on the safety and efficacy of immune checkpoint inhibitor therapy in this population remain limited. A subgroup analysis of the IMbrave150 trial confirmed overall survival (OS) and progression-free survival (PFS) benefit in patients aged ≥65 years with a similar safety profile, but 55 patients only were older than 75 years.[5] Japanese studies have reported similar efficacy and good tolerability of immunotherapy (>90% atezolizumab plus bevacizumab [AtezoBev]) beyond 75 years of age[6,7] while an international study by Vithayathil *et al.*[8] showed no significant OS differences according to age, albeit using a 65-year cut-off. In the French CHIEF cohort study, the mean age was 69 years, with only 49 patients over 70 years.[9]

In real-world practice, elderly patients are frequently under-represented or excluded from immunotherapy, leading to uncertainty about the benefit–risk balance of AtezoBev in this

* Corresponding author. Address: Department of Hepatogastroenterology, University Hospital of Caen, Côte de Nacre, 14033, Caen, France. Tel.: +33 231 064 544; fax: +33 231 064 545.
E-mail address: metivier-c@chu-caen.fr (C. Métivier).
† Authors share co-first authorship.

https://doi.org/10.1016/j.jhepr.2026.101827

subgroup. Given the frailty, multiple comorbidities and modified pharmacokinetics[10,11] associated with advanced age, further evidence is needed to guide treatment decisions. The aim of the study was to assess, in real-world practice, the efficacy, safety, and prognostic factors of AtezoBev in European ancestry patients aged ≥75 years with advanced and/or unresectable HCC, compared with those younger than <75 years.

Materials and methods

Study design

This was a retrospective, observational, multicenter study conducted across eight academic tertiary care setting centers in France (Beaujon Hospital, Avicenne Hospital, La Pitié-Salpêtrière Hospital, Henri Mondor Hospital, Saint Antoine Hospital, Sud Francilien Hospital Center, Toulouse University Hospital, and Caen University Hospital) over the period from January 2020 to December 2024. In accordance with Commission nationale de l'informatique et des libertés (CNIL) guidelines, the database was pseudo-anonymized and recorded on the secure server of the University Hospital of Caen with restricted access to the project team. This research project constitutes 'Research on Data' according to the Article L. 1121-1 of the Public Health Code (Law No. 2012-3000 of March 5, 2012, and its implementing decree No. 2016-1537 of November 16, 2016). This automated processing of health data

Table 1. Baseline characteristics of patients treated with AtezoBev included in the analysis after propensity score matching (n = 484).

Baseline characteristics	Available data	Elderly patients (≥75 years) n = 242	Available data	Non-elderly patients (<75 years) n = 242	*p* value
Sex (male)	242	209 (86)	242	203 (84)	0.443
Body mass index (kg/m^2) °	239	26.0 (23.8, 29.1)	242	26.0 (22.5, 30.1)	0.669
Obesity	239	43 (18)	241	62 (26)	**0.043**
Type 2 diabetes	242	117 (48)	242	104 (43)	0.236
Arterial hypertension	242	179 (74)	242	144 (60)	**<0.001**
Dyslipidemia	242	88 (36)	242	70 (29)	0.081
Anticoagulation	237	53 (22)	233	24 (10)	**<0.001**
Cirrhosis	242	160 (66)	242	171 (71)	0.252
ECOG 0–1	224	199 (89)	200	186 (93)	0.139
ECOG 2–3		25 (11)		14 (7)	
Etiologies of liver disease					
At least ALD	242	107 (44)	242	99 (41)	0.462
At least MASLD	242	110 (45)	242	110 (45)	>0.999
At least viral	242	54 (22)	242	67 (28)	0.172
Mixed etiologies	242	99 (41)	242	97 (40)	0.853
Liver function					
Previous cirrhosis decompensation	238	28 (12)	234	24 (10)	0.567
MELD		8.0 (7.0, 10.0)		8.0 (7.0, 10.0)	
Child–Pugh A	239	214 (90)	240	219 (91)	0.485
Child–Pugh B		25 (10)		20 (8.6)	
Child–Pugh C		0 (0)		1 (0)	
mALBI grade 1	233	69 (30)	232	86 (37)	**0.038**
mALBI grade 2a		73 (31)		46 (20)	
mALBI grade 2b		83 (36)		92 (40)	
mALBI grade 3		8 (3.4)		8 (3.4)	
No EV	225	138 (61)	231	138 (60)	0.876
EV (regardless the size)		88 (39)		92 (40)	
Large size EV		41 (18)		44 (19)	0.317
Platelet count (× 10^3/mm^3)	237	179 (125, 250)	234	173 (117, 252)	0.547
Creatinine (μmol/L)	235	81 (66, 103)	234	70 (59, 87)	**<0.001**
Total bilirubin (μmol/L)	236	12 (9, 19)	233	13 (9, 18)	0.472
Albumin (g/L)	233	36 (33, 39)	236	36 (33, 40)	0.566
INR°	227	1.1 (1.0, 1.2)	222	1.1 (1.0, 1.2)	0.148
HCC features					
Previous HCC treatment	220	145 (66)	235	151 (64)	0.712
BCLC-A	241	2 (1)	242	4 (2)	0.173
BCLC-B		80 (33)		93 (38)	
BCLC-C		160 (66)		145 (60)	
AFP (ng/ml)	229	59 (5, 9)	234	31 (6, 6)	0.801
AFP >20 ng/ml	229	132 (58)	234	123 (53)	0.272
AFP >400 ng/ml	229	74 (32)	234	67 (29)	0.389
>3 lesions	241	129 (54)	238	120 (51)	0.591
Size of the largest lesion (mm)	223	51 (24, 80)	214	47 (24, 80)	0.684
Tumor size >5 cm	223	116 (52)	214	105 (49)	0.537
Extrahepatic lesions	242	74 (31)	242	71 (29)	0.766
Vascular invasion	242	65 (27)	242	69 (29)	0.684
AtezoBev treatment					
Treatment duration (months)	242	4.8 (1.5, 12)	242	5.1 (2.1, 11.7)	0.207
Second-line treatment	205	62 (30)	203	79 (39)	0.066

Data are presented as n (%) or median (IQR). *p* values in bold indicate statistical significance (p <0.05). AFP, alpha-fetoprotein; ALD, alcoholic liver disease; AtezoBev, atezolizumab–bevacizumab; BCLC: Barcelona Clinic Liver Cancer; ECOG, Eastern Cooperative Oncology Group; EV, esophageal varices; HCC, hepatocellular carcinoma; INR, international normalized ratio; mALBI grade, modified albumin–bilirubin grade; MASLD, metabolic dysfunction-associated steatotic liver disease; MELD, model for end-stage liver disease.

complies with the European Regulation of April 27, 2016, concerning the protection of individuals regarding the processing of personal data and the free movement of such data. The protocol was approved by the following Research Ethics Committees (ID Number 2024112619344200000180001697, APHP210978/IDRCB Number: 2021-A01343-38).

Patient selection

Adults (≥18 years) who received first-line AtezoBev at participating centers, identified through pharmacy records, were eligible if they had advanced or unresectable HCC confirmed histologically or on imaging according to EASL criteria.[2] When no hepatic target was available, diagnosis was confirmed on histological analysis of metastasis. Cirrhosis was diagnosed based on histological findings or a combination of clinical, radiological, and biological criteria, and its etiology was documented by the treating physician. Patients with benign liver tumors, other primary liver malignancies, or prior systemic therapy were excluded. Patients aged 75 years or older were considered elderly.

Data collection

For each patient, baseline clinical, radiological, and laboratory data were collected immediately before initiation of AtezoBev. Variables included age, sex, comorbidities (type 2 diabetes, arterial hypertension, body mass index [BMI]), etiology of liver disease, presence of cirrhosis and its complications; tumor characteristics (number of nodules, size of the largest lesion, macrovascular invasion, and extrahepatic metastases); Barcelona Clinic Liver Cancer (BCLC) stage; and liver function parameters (Child–Pugh score and modified albumin–bilirubin [mALBI] grade). The ALBI grade was calculated based on serum albumin and total bilirubin values using the following formula: ALBI score: ($\log_{10}$ bilirubin (µmol/L) × 0.66) + (albumin (g/L) × -0.085). ALBI grade was defined by the score of the following: ≤-2.60 = grade 1, >-2.60 to ≤-1.39 = grade 2, >-1.39 = grade 3. ALBI grade 2 was further divided into two subgrades (2a and 2b) using ALBI score -2.270 as the cut-off value. The four ALBI grades were named as mALBI grade. Blood tests comprised total bilirubin, INR, alpha-fetoprotein

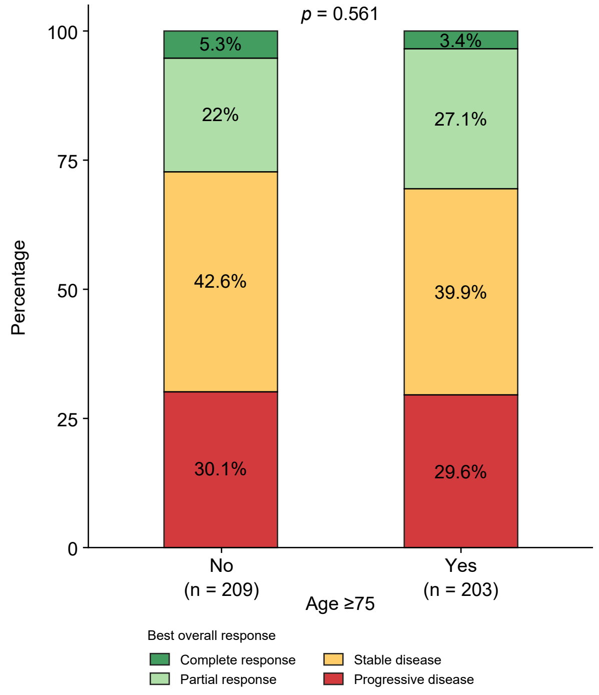

Fig. 1. Overall response and disease control by age group (n = 412). Stacked bar plots compare outcomes between patients aged <75 years (‘No’) and ≥75 years (‘Yes’) after propensity score matching. Distribution of best overall response categories (Complete Response, Partial Response, Stable Disease, Progressive Disease). Percentages of each class are reported within the bars, and sample sizes (n) are shown for each age group on the x-axis. Only patients who underwent radiological tumor assessment were included; patients who died or experienced symptomatic progression before radiological evaluation were excluded corresponding to the exclusion of 33 non-elderly and 39 elderly patients, respectively.

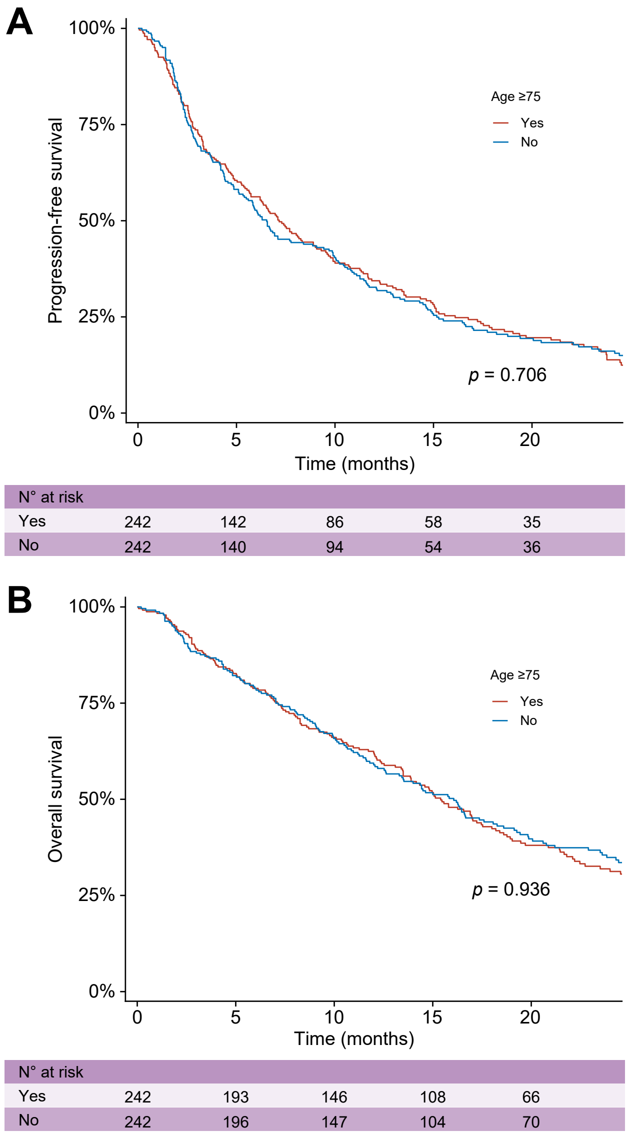

Fig. 2. Kaplan–Meier curves for progression-free and overall survival according to age group. Kaplan–Meier estimates of progression-free survival (A) and overall survival (B) according to age group (<75 years *vs.* ≥75 years) after propensity score matching. Survival curves were compared using the log-rank test, n = 412.

Table 2. Baseline predictive factors of progression-free survival in patients aged ≥75 years treated with AtezoBev included in the analysis after propensity score matching (Cox proportional hazard regression models, n = 242).

			Univariate analysis			Multivariate analysis		
	**Patients**	**Events**	**HR**	**95% CI**	***p* value**	**HR**	**95% CI**	***p* value**
Sex (male)	209	170	0.77	0.52, 1.13	0.186			
Obesity	239	197	0.97	0.67, 1.41	0.878			
Type 2 diabetes	243	200	0.87	0.66, 1.15	0.340			
Arterial hypertension	242	200	0.76	0.55, 1.06	0.104			
Dyslipidemia	242	200	1.18	0.89, 1.57	0.254			
Anticoagulation	237	195	0.91	0.65, 1.27	0.572			
Cirrhosis	242	200	0.85	0.63, 1.14	0.276			
ECOG 0–1	199	164	—	—	—			
ECOG 2–3	25	22	1.28	0.82, 2.00	0.274			
At least ALD	242	200	1.07	0.81, 1.41	0.637			
At least MASLD	242	200	1.03	0.78, 1.37	0.815			
At least viral	242	200	0.89	0.63, 1.24	0.484			
Mixed etiologies	242	200	0.99	0.75, 1.32	0.960			
Esophageal varices	225	186	1.12	0.84, 1.50	0.452			
Large size EV	41	36	1.07	0.91, 1.27	0.400			
Previous cirrhosis decompensation	237	196	1.54	0.97, 2.46	0.067			
Platelet count	237	196	1.00	1.00, 1.00	0.234			
Albumin	233	193	0.94	0.91, 0.97	**<0.001**			
Total bilirubin	236	195	1.02	1.00, 1.03	**0.025**			
Creatinine	235	194	1.00	1.00, 1.00	0.530			
INR	227	186	1.57	0.83, 2.93	0.162			
MELD	231	190	1.01	0.97, 1.05	0.537			
Child–Pugh A	214	175	-2.24	-1.44, 3.48	**-<0.001**			
Child–Pugh B	25	23						
mALBI grade 1	69	52	—	—	—	—	—	—
mALBI grade 2a	73	59	1.32	0.90, 1.92	0.150	1.36	0.92, 2.01	0.124
mALBI grade 2b	83	74	1.77	1.24, 2.53	**0.002**	1.81	1.25, 2.62	**0.002**
mALBI grade 3	8	8	4.14	1.94, 8.81	**<0.001**	4.37	2.04, 9.37	**<0.001**
Previous HCC treatment	220	185	1.14	0.84, 1.56	0.404			
BCLC-A	1	1	—	—	—			
BCLC-B	80	63	0.13	0.02, 0.93	0.052			
BCLC-C	160	136	0.16	0.02, 1.19	0.073			
AFP (ng/ml)	229	187	1.00	1.00, 1.00	**0.030**	1.00	1.00, 1.00	**0.012**
AFP >20 ng/ml	229	110	1.32	0.98, 1.76	0.064			
AFP >400 ng/ml	229	65	1.31	0.97, 1.77	0.083			
>3 lesions	241	200	1.09	0.83, 1.44	0.540			
Size of the largest lesion (mm)	223	183	1.00	1.00, 1.00	0.324			
Tumor size >5 cm	223	183	0.91	0.68, 1.21	0.507			
Extrahepatic lesions	242	200	1.32	0.98, 1.79	0.068			
Vascular invasion	242	200	1.01	0.74, 1.38	0.928			

The ECOG performance status was not included in the multivariate analysis because the proportion of missing values exceeded the predefined 10% threshold for imputation. *p* values in bold indicate statistical significance (p <0.05).
The Child–Pugh score, MELD score, albumin, and bilirubin were not entered in the multivariate analysis to avoid collinearity with ALBI grade. AFP, alpha-fetoprotein; ALBI, albumin–bilirubin grade; ALD, alcoholic liver disease; AtezoBev, atezolizumab–bevacizumab; BCLC: Barcelona Clinic Liver Cancer; EV, esophageal varices; HCC, hepatocellular carcinoma; HR, hazard ratio; INR, international normalized ratio; mALBI grade, modified albumin–bilirubin grade; MASLD, metabolic dysfunction-associated steatotic liver disease; MELD, model for end-stage liver disease.

(AFP), albumin, creatinine, and platelet count. The diagnosis of metabolic dysfunction-associated steatotic liver disease (MASLD) was made according to the presence of hepatic steatosis (identified either through imaging or histological analysis) in combination with at least one cardiometabolic risk factor (current or prior BMI >30 kg/m^2, type 2 diabetes mellitus, hypertension, or abnormal lipid profiles) and none or low alcohol intake (<20 g/day in women and <30 g/day in men). Cirrhosis was defined as having at least an alcoholic, viral, or metabolic etiology when it was related, respectively, to chronic alcohol consumption, viral infection, or metabolic syndrome, with or without an associated additional cause of chronic liver disease (alcoholic, viral, metabolic). During follow-up, adverse events (AEs) were graded according to the Common Terminology Criteria for AEs, version 5.0. Tumor response to treatment was assessed and categorized as complete response (CR), partial response (PR), stable disease (SD), or progressive disease (PD) according to RECIST 1.1 criteria.[2] Objective response rate (ORR) was defined as CR or PR, while disease control rate (DCR) was defined as CR, PR, or SD considering the best response during follow-up. In addition, subsequent treatments administered after AtezoBev were also collected. Patients were followed until death, progression, or December 20, 2024 for patients who remained alive and progression-free. The causes of death were recorded, as well as the time between death and the end of treatment, when applicable.

Statistical analysis

Continuous variables are described as median and IQR, and categorical variables as numbers and percentages. The non-parametric Mann–Whitney *U* test was used to compare

Table 3. Baseline predictive factors of mortality in patients aged ≥75 years treated with AtezoBev included in the analysis after propensity score matching (Cox proportional hazard regression models, n = 242).

			Univariate analysis			Multivariate analysis		
	Patients	Events	HR	95% CI	*p* value	HR	95% CI	*p* value
Sex (male)	242	140	1.02	0.64, 1.61	0.948			
Obesity	239	158	0.81	0.52, 1.26	0.351			
Type 2 diabetes	243	161	0.91	0.67, 1.24	0.564			
Arterial hypertension	242	161	0.87	0.62, 1.22	0.424			
Dyslipidemia	242	161	1.36	0.99, 1.87	0.056			
Anticoagulation	237	156	1.05	0.73, 1.52	0.775			
Cirrhosis	242	161	0.84	0.60, 1.16	0.289			
ECOG 0–1	199	128	—	—	—			
ECOG 2–3	25	20	1.60	1.00, 2.57	0.051			
At least ALD	242	161	1.54	0.98, 2.41	0.062			
At least MASLD	242	161	0.79	0.52, 1.19	0.256			
At least viral	242	161	0.67	0.39, 1.15	0.143			
Mixed etiologies	242	161	1.06	0.78, 1.45	0.697			
Esophageal varices	225	149	1.21	0.87, 1.67	0.259			
Large size EV	41	30	1.11	0.92, 1.33	0.280			
Previous cirrhosis decompensation	237	157	1.28	0.76, 2.14	0.356			
Platelet count	237	159	1.00	1.00, 1.00	0.270			
Albumin	233	158	0.93	0.90, 0.96	**<0.001**			
Total bilirubin	236	158	1.01	1.00, 1.03	**0.040**			
Creatinine	235	158	1.00	1.00, 1.00	0.633			
INR	227	152	1.92	1.07, 3.43	**0.029**	1.42	0.69, 2.93	0.337
MELD	231	156	1.04	1.00, 1.07	0.062			
Child–Pugh A	214	137	—	—	—			
Child–Pugh B	25	22	2.24	1.42–3.54	**<0.001**			
mALBI grade 1	69	38	—	—	—	—	—	–
mALBI grade 2a	73	51	1.49	0.98, 2.28	0.062	1.59	1.02, 2.50	**0.043**
mALBI grade 2b	83	61	1.91	1.27, 2.88	**0.002**	1.90	1.20, 2.93	**0.006**
mALBI grade 3	8	8	5.53	2.54, 12.0	**<0.001**	5.62	2.47, 12.8	**<0.001**
Previous HCC treatment	220	148	0.81	0.58, 1.13	0.210			
BCLC-A	1	1	—	—	—			
BCLC-B	80	46	0.12	0.02, 0.91	**0.040**			
BCLC-C	160	114	0.18	0.02, 1.32	0.092			
AFP (ng/ml)	229	150	1.00	1.00, 1.00	**<0.001**			
AFP >20 ng/ml	229	92	1.44	1.03, 2.00	**0.031**	1.38	0.98, 1.94	0.068
AFP >400 ng/ml	229	59	1.30	0.93, 1.82	0.124			
>3 lesions	241	161	1.03	0.75, 1.40	0.865			
Size of the largest lesion (mm)	223	148	1.00	1.00, 1.00	0.247			
Tumor size >5 cm	223	148	1.32	0.95, 1.82	0.096			
Extrahepatic lesions	242	161	1.25	0.89, 1.75	0.194			
Vascular invasion	242	161	1.29	0.92, 1.81	0.136			

ECOG performance status was not included in the multivariate analysis because the proportion of missing values exceeded the predefined 10% threshold for imputation. *p* values in bold indicate statistical significance (*p* <0.05). Child–Pugh score, MELD score, albumin, and bilirubin were not entered in the multivariate analysis to avoid collinearity with ALBI grade. AFP, alpha-fetoprotein; ALD, alcoholic liver disease; AtezoBev, atezolizumab–bevacizumab; BCLC, Barcelona Clinic Liver Cancer; EV, esophageal varices; HCC, hepatocellular carcinoma; INR, international normalized ratio; mALBI grade, modified albumin–bilirubin grade; MASLD, metabolic dysfunction-associated steatotic liver disease; MELD, model for end-stage liver disease.

quantitative variables and X^2 test for categorical variables. Follow-up of the patients was recorded until December 20, 2024. OS was calculated from the date of AtezoBev initiation to the date of death or at the time of the last follow-up. PFS was calculated from the date of AtezoBev initiation to the date of disease progression or death. Kaplan–Meier curves and the log-rank test were used to evaluate OS and PFS. Univariable and multivariable survival analyses were carried out using the Cox model. Variables included in the multivariable models were selected based on univariable analyses, and all covariates with a value of *p* <0.05 were entered into the multivariable models. Collinearity among covariates was evaluated using variance inflation factors (VIFs), and no significant multicollinearity was observed, with all VIF values <5. The proportional hazards assumption was assessed using Schoenfeld residuals. To account for baseline differences between very elderly patients (≥75 years) and younger counterparts (<75 years), a propensity score matching (PSM) approach was applied. The propensity score for being aged ≥75 years was estimated using a multivariable logistic regression model including clinically relevant covariates: presence of cirrhosis, serum albumin and bilirubin concentrations, size of the largest tumor nodule, presence of more than three nodules, extrahepatic metastases, macrovascular invasion, viral etiology (hepatitis B and C), alcohol- or metabolism-related etiology, and history of hepatic decompensation. Eastern Cooperative Oncology Group (ECOG) performance status was not included in the propensity score model because of the high proportion of missing data, which precluded reliable imputation. Missing data for covariates included in the propensity score model were imputed before matching. Continuous variables with <10% missing data were

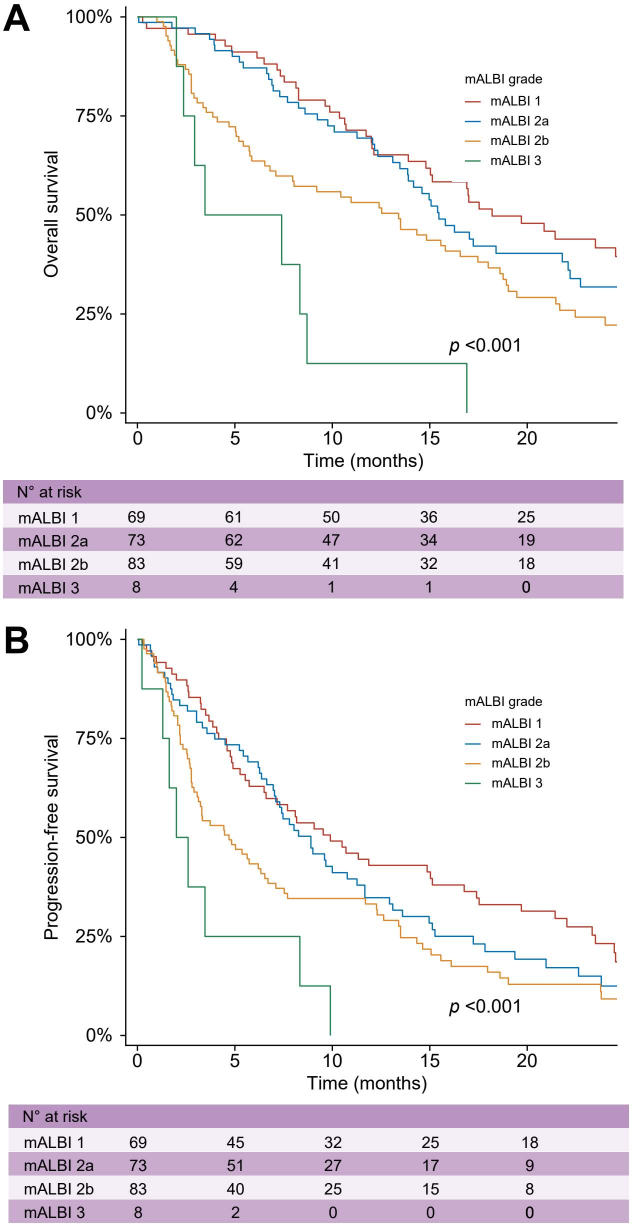

Fig. 3. Overall and progression-free survival according to mALBI grade in the elderly group. Kaplan–Meier curves showing (A) overall survival (OS) and (B) progression-free survival stratified by mALBI grade at treatment initiation in patients ≥75 years old after propensity score matching. The number of patients at risk is shown below each plot. mALBI, modified albumin–bilirubin grade.

imputed using the median value, and categorical variables with >10% missing data were imputed using the most frequent category (mode). Matching was performed using the nearest-neighbor method without replacement, with a 1:1 ratio and a caliper width of 0.2 of the standard deviation of the logit of the propensity score. Baseline characteristics were compared before and after matching and graphical assessment of balance was performed using density plots and Love plot (Supplementary Data 1). A two-sided value of p <0·05 was considered statistically significant. All statistical analyses were performed using R version 4.5.1 (R Foundation for Statistical Analysis, Vienna, Austria).

Results

Baseline characteristics of patients

Between January 2020 and December 2024, 814 patients with advanced and/or unresectable HCC were treated with Atezo-Bev as first-line systemic therapy. A total of 169 patients were included from Beaujon Hospital, 140 patients from Avicenne Hospital, 125 patients from La Pitié-Salpêtrière Hospital, 109 patients from Henri Mondor Hospital, 64 patients from Saint Antoine Hospital, six patients from Sud Francilien Hospital Center, 96 patients from Toulouse University Hospital, and 105 patients from Caen University Hospital. No differences in patient characteristics were observed between centers (Supplementary Data 2). Of these, 566 patients (69.5%) were <75 years (median age 64; 84% male) and 248 (30.5%) were ≥75 years, defined as the elderly group (median age 78; 86% male) (Supplementary Data 3). Compared with non-elderly patients, elderly patients had more arterial hypertension (75% *vs.* 54%), dyslipidemia (36% *vs.* 22%), and anticoagulation use (23% *vs.* 15%), but less cirrhosis (65% *vs.* 78%), fewer prior decompensations (12% *vs.* 24%), and less viral etiology (22% *vs.* 48%). Patients aged ≥75 years had more Child–Pugh A disease (90% *vs.* 80%); tumor burden was broadly similar, but AFP was lower (median 54 *vs.* 95 ng/ml) and vascular invasion less frequent (27% *vs.* 42%) (Supplementary Data 4). Median OS (<75 years *vs.* elderly: 14.4 *vs.* 15.5 months) and PFS (<75 years *vs.* elderly: 6.1 *vs.* 7.3 months) were similar regardless of the age status in the whole cohort Supplementary Data 5.

Owing to significant differences in liver function, cause of liver disease, and HCC burden, we performed PSM and 484 patients were included in the final analysis (Supplementary Data 1). After matching, patients aged ≥75 years more often received anticoagulation (22% *vs.* 10%; p <0.001), had more hypertension (74% *vs.* 60%; p <0.001) and less obesity (18% *vs.* 26%; p = 0.043), while liver disease parameters and HCC characteristics were otherwise comparable between groups (Table 1).

Comparable outcomes in patients ≥75 years *vs.* <75 years after PSM

Median duration of AtezoBev was similar between patients ≥75 years (4.8 months [1.5–12.0]) and those <75 years (5.1 months [2.1–11.7]) (p = 0.207). Atezolizumab was continued as monotherapy in 21% and 24% of patients, respectively (p = 0.162). The best response rate was also comparable (Fig. 1): ORR 30.5% *vs.* 27.3% and DCR 70.4% *vs.* 69.9% in ≥75 *vs.* <75 years, respectively.

After a median follow-up of 28.0 months (95% CI 26.6–30.0), median PFS was 6.7 months (6.0, 8.0) in the whole cohort, 7.2 months (6.2, 9.0) in patients ≥75 years, and 6.5 months (5.5, 9.1) in those <75 years (p = 0.706; Fig. 2A).

In the whole cohort, mALBI grade 2b (HR 1.59; 95% CI 1.22, 2.06; p <0.001) and mALBI grade 3 (HR 3.77; 95% CI 2.12–6.70; p = 0.006), as well as the presence of extrahepatic metastases (HR 1.48; 95% CI 1.17–1.88; p = 0.001) and AFP >400 ng/ml (HR 1.48; 95% CI 1.16, 1.88; p = 0.002), were independently associated with PFS in multivariate analysis, but

not an age ≥75 years (Supplementary Data 6). A trend toward an association was also observed for ECOG 2–3 (HR 1.42; 95% CI 0.97–2.08; p = 0.068.

Across 325 deaths, cause distribution was similar in patients ≥75 and <75 years: HCC progression 49.7% *vs.* 48.8%, liver decompensation 8.1% *vs.* 6.7%, non-liver causes 1.9% *vs.* 1.8%, with unknown cause in 40.4% *vs.* 42.7%. Median OS was 15.8 months (14.0, 17.0) in the whole cohort, 15.4 months (13.5, 17.5) in elderly patients and 16.1 months (13.4, 18.6) in those <75 years (p = 0.936; Fig. 2B). In the multivariate analysis, mALBI grade 2b (HR 1.60; 95% CI 1.13, 2.26; p = 0.007) and mALBI grade 3 (HR 4.58; 95% CI 2.36, 8.88; p <0.001), as well as ECOG 2–3 (HR 1.56; 95% CI 1.01–2.42; p = 0.046) and AFP >400 ng/ml (HR 1.46; 95% CI 1.09, 1.96; p = 0.011), were independently associated with mortality, but not an age ≥75 years (Supplementary Data 7).

mALBI grade 3 after PSM is predictive of poorer outcomes in patients ≥75 years treated with AtezoBev

In the cohort of patients aged ≥75 years treated with AtezoBev (n = 242), univariate analysis identified several baseline factors associated with both progression and mortality, particularly parameters reflecting liver function (Tables 2 and 3). A clear gradient in mortality was observed across mALBI grades (p <0.001), ranging from 55.1% in mALBI 1 to 100% in mALBI 3 (four from HCC progression, four unknown). The mALBI classification showed a clear stepwise association with survival outcomes. In multivariable Cox models adjusted for significant covariates, the mALBI grade remained independently associated with both PFS and OS, confirming that impaired hepatic reserve strongly influences long-term outcomes in elderly patients receiving AtezoBev. Compared with patients with mALBI 1, those classified as mALBI 3 exhibited significantly higher risks of disease progression (HR 4.37, 95% CI 2.04, 9.37, p <0.001) (Table 2) and death (HR 5.62, 95% CI 2.47, 12.8, p <0.001) (Table 3). In mALBI 3 median OS and PFS were 5.43 months and 2.3 months respectively (Fig. 3). Baseline characteristics of patients with an mALBI score of 3 were similar between elderly and non-elderly patients, except for a higher prevalence of dyslipidemia and use of beta-blockers among elderly patients (Supplementary Data 8). mALBI grade 3 was also associated with a poor prognosis in non-elderly patients (Fig. 5).

Distribution of adverse events in ≥75 years after PSM

Any grade AEs occurred in 80% and 75% of patients, in <75 years and ≥75 years (p = 0.254). Acute variceal bleeding occurred in 16% and 13% (p = 0.336). The only difference between groups concerned the immune-related adverse events (IRAEs) which were more frequent in patients <75 years compared with those ≥75 years, both when including thyroid dysfunction (37% *vs.* 22%; p <0.001) and when excluding it (25.9% *vs.* 15.9%; p = 0.010) (Table 4). In univariate analysis among the 242 elderly patients, OS and PFS were longer in those who developed hypertension (p = 0.040 and p = 0.090, respectively), proteinuria (p <0.001 and p = 0.015, respectively) and IRAEs (p = 0.020 and p = 0.007, respectively). Among them, 39 developed on-treatment hypertension and 184 did not. Baseline hypertension was similar in the two groups (79% [31/39] *vs.* 72% [133/184]; p = 0.354) (Supplementary Data 9). Proteinuria occurred in 27.3% of patients who developed hypertension during treatment, compared with only 2.8% among those without hypertension (p <0.001) (Fig. 4). The presence of hypertension was strongly associated with the occurrence of proteinuria (OR [odds ratio] = 13.1; 95% CI 4.1–42.4). Treatment duration was longer in patients who developed hypertension (median 8.3 months [2.8–14.8] *vs.* 4.1 months [1.4–9.7]; p = 0.009). Notably, baseline hypertension did not predict on-treatment hypertension (p = 0.400) (Supplementary Data 10).

In addition, 20 patients also developed on-treatment proteinuria. Baseline hypertension was similar between patients who developed or who did not develop proteinuria under treatment (85% [17/20] *vs.* 72% [147/203]; p = 0.23) (Supplementary Data 11). However, hypertension occurred more frequently in those with proteinuria than in those without (55% *vs.* 14%; p <0.001). Treatment duration was also longer in patients who developed proteinuria (median 13.0 [5.1–15.2]

Table 4. Incidence of treatment-related adverse events in elderly *vs.* non-elderly patients treated with AtezoBev.

Adverse events	Overall N = 484 (%)	Elderly patients (≥75 years) n = 242 (%)	Non-elderly patients (<75 years) n = 242 (%)	*p* value
Any AE	337 (77)	168 (75)	169 (80)	0.254
Grade 3–4 AE	100 (20)	60 (21)	40 (19)	0.721
Asthenia	199 (46)	110 (50)	89 (41)	0.079
Ascites	94 (20)	52 (22)	42 (18)	0.268
Hepatic encephalopathy	38 (8)	20 (8.5)	18 (7.7)	0.756
Variceal bleeding	67 (14)	30 (13)	37 (16)	0.336
Arterial hypertension	66 (15)	39 (17)	27 (13)	0.144
Proteinuria	39 (9)	20 (9)	19 (9)	0.949
Hand–foot syndrome	11 (3)	5 (2)	6 (3)	0.720
Immune-related AE including dysthyroidism				
Any immune-related AE	130 (29)	50 (22)	80 (37)	**<0.001**
Steroid use	31 (25)	16 (31)	15 (21)	0.207
Hepatitis	10 (2)	4 (2)	6 (3)	0.538
Myositis	12 (3)	3 (1)	9 (4)	0.079
Skin rash	58 (13)	24 (11)	34 (16)	0.114
Thyroid dysfunction	51 (12)	19 (9)	32 (15)	**0.040**
Immune-related AE excluding dysthyroidism	92 (21)	36 (15.9)	56 (25.9)	**0.010**

AE, adverse events. *p* values in bold indicate statistical significance (*p* <0.05)

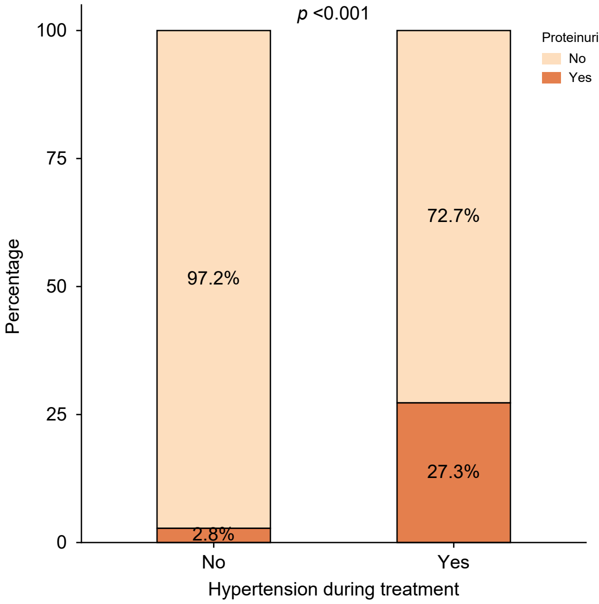

Fig. 4. Association between hypertension and proteinuria during treatment. Stacked bar plot showing the proportion of patients who developed proteinuria according to the presence or absence of hypertension during treatment.

months) than in those without proteinuria (4.2 [1.4–10.3 months]; p = 0.003).

IRAEs occurred in 130 patients. Notably, acute variceal bleeding was more common in those who developed such IRAEs (27% [13/50]) than in those who did not (8% [14/176]; p <0.001). Treatment duration was also longer in patients with IRAEs (median 10.6 [2.8–19.0] months *vs.* 4.1 [1.5–8.9] months; p <0.001) (Supplementary Data 12).

Discussion

In our large European ancestry cohort, patients aged ≥75 years with advanced or unresectable HCC treated with AtezoBev had a median OS of 15.1 months, comparable to that in younger patients, without excess toxicity and similar response rate. However, mortality was significantly higher in elderly patients with mALBI grade 3, supporting cautious use of AtezoBev in this subgroup, while the occurrence of treatment-related AEs correlated with better outcomes.

Advanced age is associated with higher rates of tyrosine-kinase inhibitor dose reductions and treatment discontinuation, often because of intolerance, thereby challenging the initiation of systemic therapy in the elderly population.[11,12] Consistent with previous reports, our results indicate that AtezoBev remains safe and effective in older patients without major bevacizumab discontinuation. However, real-world data in very elderly patients, particularly in populations of European ancestry, are scarce. Extending conclusions from studies with lower age cut-offs, smaller sample size, or Asian-only cohorts remains controversial given the frailty and comorbidities characteristics of this age group.[3,5–8] In the recent Japanese propensity score matched study by Kaneko *et al.*,[7] outcomes were similar between 112 patients aged ≥75 years and 112 patients aged <75 years, with a median OS of 25.6 months and 19.7 months respectively (p = 0.24). The longer survival

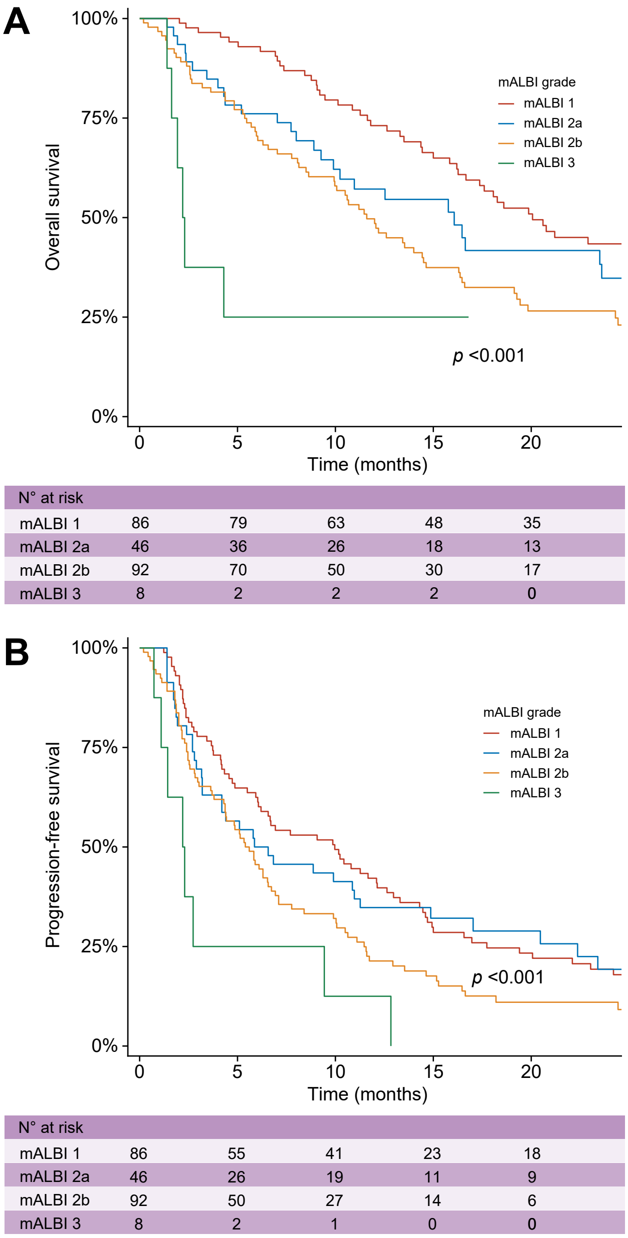

Fig. 5. Overall and progression-free survival according to mALBI grade in the non-elderly group. Kaplan–Meier curves showing (A) overall survival and (B) progression-free survival stratified by mALBI grade at treatment initiation in patients <75 years old after propensity score matching. The number of patients at risk is shown below each plot. mALBI: modified albumin–bilirubin grade.

observed in Japanese cohort compared with ours likely reflects differences in baseline characteristics, including a greater proportion of viral diseases (34% *vs.* 22%), earlier BCLC stages (BCLC B60.7% *vs.* 33 %), and a lower baseline AFP level (15.1 *vs.* 59 ng/ml) (7). Liver function, as reflected by mALBI grade, was independently associated with both OS and PFS. In our study mALBI 3 was the strongest prognostic factor for adverse outcomes, confirming its pivotal role in patients' prognosis. Nevertheless, the original ALBI classification has recognized limitations, as most real-world patients cluster within ALBI 2, making this group highly heterogeneous in terms

of residual liver function and prognosis. To overcome this limitation, the mALBI grade was developed. In a large cohort of patients with unresectable HCC treated with lenvatinib, median OS was approximately 22.5 months for mALBI 2a, 10.3 for mALBI 2b and 5.1 for mALBI3, while Child–Pugh failed to distinguish outcomes as effectively.[13] Our findings confirm the independent prognostic value of mALBI even in the setting of AtezoBev, particularly among elderly patients. In our cohort aged ≥75 years, mALBI grades showed a stepwise and significant association with OS and PFS. Patients with mALBI 3 had a significantly greater risk of disease progression (HR 4.37) and mortality (HR 5.62) compared with those with mALBI 1, even after adjustment for clinical covariates.

In our series treatment duration and overall tolerability were comparable across age groups, except for immune-related AEs which were more frequent in younger patients. This observation may reflect immunosenescence and reduce immune-reactive profile beyond 75 years.[14] Moreover, a similar proportion of elderly and younger patients received second-line therapy. Together, these findings support the safety of AtezoBev in very elderly European ancestry patients and confirm that immunotherapy does not compromise subsequent treatment. Treatment duration was longer among patients who developed hypertension, proteinuria or IRAEs (including or excluding thyroid dysfunction). No relationship was observed between baseline hypertension and the occurrence of hypertension or proteinuria during treatment. Yang *et al.*[15] reported that lower baseline vascular endothelial growth factor-D in patients with proteinuria were related to a better prognosis[7,15,16] suggesting that proteinuria may be a surrogate marker of effective anti-angiogenic treatment. IRAEs have already been correlated with improved radiological response, particularly when hypothyroidism develops, although associations with OS remain to be elucidated.[16,17]

Despite relative good tolerance and efficacy of AtezoBev in this series of very elderly patients, mALBI 3 could not really benefit from immunotherapy. Other recent evidence shows that systemic chemotherapy often provides little survival or functional benefit for very old, while causing substantial toxicity.[18] Consequently, ASCO's recent geriatric oncology guidance emphasizes routine geriatric assessment to identify vulnerabilities and to individualize or withhold systemic treatment when expected benefit is minimal, supporting avoidance of potentially futile, costly chemotherapy in appropriately selected very old patients.[19] For this reason our results raise the question of AtezoBev futility in patients with altered hepatic function in this particular population.

The strengths of our study include its real-world, multicenter design within a European ancestry population, the large sample size and the use of PSM to minimize confounding. However, several limitations should be acknowledged. First, the retrospective design inherently limits data completeness and standardization across centers. Detailed information on previous antihypertensive therapy, treatment adherence, and dose adjustments of bevacizumab was not systematically available. Central radiological review could not be performed, which may have introduced variability in response assessment. In addition, multivariate analysis including time-dependent factors were not conducted, preventing assessment of how on-treatment events such as hypertension, proteinuria, or immune-related AE influenced survival outcomes. Lastly, although our results suggest a particularly poor prognosis in patients with mALBI grade 3, potentially questioning the initiation of immunotherapy in this subgroup, these findings must be interpreted with caution given the small sample size in both elderly and non-elderly patients. Larger studies are warranted to confirm these observations.

AtezoBev is efficient and safe as first-line treatment in very elderly patients of European ancestry with advanced and/or unresectable HCC when liver function is preserved. Caution is highly warranted in elderly patients with mALBI grade 3.

Affiliations

[1]*Department of Hepatogastroenterology, Caen University Hospital, Caen, France;* [2]*INSERM Unit U1086 ANTICIPE, Caen, France;* [3]*Functional Genomics of Solid Tumors Team, Cordeliers Research Center, Sorbonne University, Inserm, Paris University, Paris, France;* [4]*Department of Hepatology, AP-HP Avicenne, Training and Research Unit for Health, Medicine and Human Biology, Paris 13 University, Sorbonne, Paris, Cité Community of Universities and Institutions, Paris, France;* [5]*Department of Hepatogastroenterology, AP-HP Sorbonne University, Pitié-Salpêtrière Hospital, Paris, France;* [6]*Biostatistics Department, Caen University Hospital, Caen, France;* [7]*AP-HP, Beaujon Hospital, Liver Cancer and Innovative Therapy, Clichy, France;* [8]*INSERM U1149, Inflammation Research Center (CRI), Paris, France;* [9]*Department of Hepatogastroenterology, AP-HP, Henri Mondor Hospital, Créteil, France;* [10]*Department of Hepatology, Toulouse University Hospital, Toulouse, France;* [11]*Department of Hepatology, AP-HP, Saint Antoine Hospital, INSERM UMRS 938, Saint Antoine Research Center, Sorbonne University, Paris, France;* [12]*Digestive Oncology Department, Francois Baclesse Center, Caen, France;* [13]*Grenoble Alpes University, Institute of Advanced Biosciences, UGA/Inserm U 1209/CNRS 5309 Research Center, La Tronche, France;* [14]*Department of Hepatogastroenterology and Digestive Oncology, Grenoble Alpes University Hospital, La Tronche, France;* [15]*Hepato-biliary Center, Paul Brousse hospital, Inserm uURM, Villejuif, Paris Saclay University, Paris, France*

Abbreviations

AEs, adverse events; AFP: alpha-fetoprotein; ALD, alcoholic liver disease; AtezoBev, atezolizumab–bevacizumab; BCLC, Barcelona Clinic Liver Cancer; BMI, body mass index; CR, complete response; DCR, disease control rate; ECOG, Eastern Cooperative Oncology Group; HCC, hepatocellular carcinoma; HR, hazard ratio; IRAEs, immune-related adverse events; mALBI, modified albumin–bilirubin grade; MASLD, metabolic dysfunction-associated steatotic liver disease; MELD, model for end-stage liver disease; OR, odds ratio; ORR, objective response rate; OS, overall survival; PD, progressive disease; PFS, progression-free survival; PR, partial response; PSM, propensity score matching; SD, stable disease; VIFs, variance inflation factors.

Financial support

No financial support was received to produce this manuscript.

Conflicts of interest

PN has received honoraria from and/or consults for AstraZeneca, Bristol-Myers Squibb, Eisai, and Roche. He received research grants from AstraZeneca, Bristol-Myers Squibb, and Eisai. IOH has received honoraria from and/or travel expenses from AstraZeneca, Roche, and AbbVie. JMP has received honoraria from AstraZeneca, Roche, AbbVie, Gilead, and IPSEN. CH has received honoraria from AbbVie, Roche, AstraZeneca, Jazz Pharmaceuticals, and Servier. PV has received honoraria from Roche. MB declares consulting fees from Bayer, MSD, Sirtex Medical, and Roche; advisory board fees from Bayer, MSD, Sirtex Medical, Eisai, AstraZeneca, Ipsen, Servier, Taiho, BMS, and Terumo, payment or honoraria for lectures from Bayer, Roche, MSD, Sirtex Medical, and AstraZeneca. CC declares research grants, speaker or travel expenses from Roche, Ipsen, AbbVie, and Gilead. J-CN has received research grants from Bayer and Ipsen. Other authors declare that they have no conflicts of interest related to the

study. NG-C received travel and congress fees, consulting fees or honoraria for lectures, presentations, speakers bureaus from AbbVie, Gilead, and Roche. Please refer to the accompanying ICMJE disclosure forms for further details.

Authors' contributions

Study conceptualization: CM, IOH, MA. Acquisition of data: all authors. Data statistical analysis: CC, CM, RM. Data interpretation: CM, IOH, CC, MA. Drafted the manuscript: CM, IOH. Critical revision of the manuscript: all authors.

Data availability

Data that support the findings of this study are available upon reasonable request from the corresponding author.

Supplementary data

Supplementary data to this article can be found online at https://doi.org/10.1016/j.jhepr.2026.101827.

References

[1] Sung H, Ferlay J, Siegel RL, et al. Global Cancer Statistics 2020: GLOBOCAN estimates of incidence and mortality worldwide for 36 cancers in 185 countries. CA Cancer J Clin 2021;71:209–249.
[2] European Association for the Study of the Liver. EASL clinical practice guidelines: management of hepatocellular carcinoma. J Hepatol 2018;69:182–236.
[3] Finn RS, Qin S, Ikeda M, et al. Atezolizumab plus bevacizumab in unresectable hepatocellular carcinoma. N Engl J Med 2020;382:1894–1905.
[4] Defossey G, Le Guyader-Peyrou S, Uhry Z, et al. Estimations nationales de l'incidence et de la mortalité par cancer en France métropolitaine entre 1990 et 2018, vol. 1. Tumeurs solides; 2019. p. 372.
[5] Li D, Toh HC, Merle P, et al. Atezolizumab plus bevacizumab versus sorafenib for unresectable hepatocellular carcinoma: results from older adults enrolled in the IMbrave150 randomized clinical trial. Liver Cancer 2022;11:558–571.
[6] Tada T, Kumada T, Hiraoka A, et al. Safety and efficacy of atezolizumab plus bevacizumab in elderly patients with hepatocellular carcinoma: a multicenter analysis. Cancer Med 2022;11:3796–3808.
[7] Kaneko S, Asahina Y, Murakawa M, et al. Effect of immunotherapy on late elderly patients with unresectable hepatocellular carcinoma: a real-world clinical study. Cancer Med 2025;14:e71171.
[8] Vithayathil M, D'Alessio A, Fulgenzi CAM, et al. Impact of older age in patients receiving atezolizumab and bevacizumab for hepatocellular carcinoma. Liver Int 2022;42:2538–2547.
[9] Allaire M, Thiam EM, Amaddeo G, et al. Real-world outcomes of atezolizumab-bevacizumab in hepatocellular carcinoma: the prospective French CHIEF cohort. Liver Int 2025;45:e70337.
[10] Soto-Perez-de-Celis E, Li D, Yuan Y, et al. Functional versus chronological age: geriatric assessments to guide decision making in older patients with cancer. Lancet Oncol 2018;19:e305–e316.
[11] Rimassa L, Personeni N, Czauderna C, et al. Systemic treatment of HCC in special populations. J Hepatol 2021;74:931–943.
[12] Hatanaka T, Kakizaki S, Hiraoka A, et al. Comparative analysis of the therapeutic outcomes of atezolizumab plus bevacizumab and lenvatinib for hepatocellular carcinoma patients aged 80 years and older: multicenter study. Hepatol Res 2024;54:382–391.
[13] Tada T, Kumada T, Hiraoka A, et al. Real-life practice experts for HCC (RELPEC) Study Group and the HCC 48 Group (hepatocellular carcinoma experts from 48 clinics in Japan). Impact of modified albumin-bilirubin grade on survival in patients with HCC who received lenvatinib. Sci Rep 2021;11:14474.
[14] Paderi A, Fancelli S, Caliman E, et al. Safety of immune checkpoint inhibitors in elderly patients: an observational study. Curr Oncol 2021;28:3259–3267.
[15] Yang Z, Suda G, Sho T, et al. Association of proteinuria with improved prognosis in unresectable hepatocellular carcinoma treated with atezolizumab and bevacizumab, and the predictive role of serum vascular endothelial growth factor D levels: a multicenter retrospective study. Hepatol Res 2025;55:433–443.
[16] Persano M, Rimini M, Tada T, et al. Adverse events as potential predictive factors of activity in patients with advanced HCC treated with atezolizumab plus bevacizumab. Target Oncol 2024;19:645–659.
[17] Suzuki K, Yasui Y, Tsuchiya K, et al. Impact of immune-related adverse events in patients with hepatocellular carcinoma treated with atezolizumab plus bevacizumab. J Gastroenterol Hepatol 2024;39:1183–1189.
[18] Fornas G, Montón-Bueno J, Tortajada T, et al. Systemic anticancer therapy near the end of life: an analysis of factors influencing treatment in advanced cancer patients. ESMO Open 2025;10:105064.
[19] Verduzco-Aguirre HC, Temin S, Bergerot CD, et al. Geriatric assessment: ASCO global guideline clinical insights. JCO Glob Oncol 2025;11:e2500309.

Keywords: Hepatocellular carcinoma; Atezolizumab–bevacizumab; Very elderly; ≥75 years.
Received 16 February 2026; accepted 6 March 2026; Available online 17 March 2026

Journal of Hepatology, Volume *8*

Supplemental information

Atezolizumab–bevacizumab in very elderly with hepatocellular carcinoma: Age alone is not a limiting factor except in ALBI grade 3

Chloé Métivier, Claudia Campani, Manon Allaire, Rémy Morello, Sarah Mouri, Eleonore Spitzer, Mohamed Bouattour, Clémence Hollande, Sabrina Sidali, Jean Charles Nault, Nathalie Ganne-Carrié, Pierre Nahon, Giuliana Amaddeo, Hélène Regnault, Paul Vigneron, Jean Marie Péron, Leila Sadek, Cecile Cussac, Marie Lequoy, Violaine Ozenne, Marie-Pierre Galais, Claire Pérignon, Louise Lebedel, Marion Habireche, Apolline Commin, Thông Dao, Charlotte Costentin, Aurore Baron, and Isabelle Ollivier Hourmand

Atezolizumab–bevacizumab in very elderly with hepatocellular carcinoma: age alone is not a limiting factor except in ALBI grade 3

Chloé Métivier, Claudia Campani, Manon Allaire, Rémy Morello, Sarah Mouri, Eleonore Spitzer, Mohamed Bouattour, Clémence Hollande, Sabrina Sidali, Jean Charles Nault, Nathalie Ganne-Carrié, Pierre Nahon, Giuliana Amaddeo, Hélène Regnault, Paul Vigneron, Jean Marie Péron, Leila Sadek, Cecile Cussac, Marie Lequoy, Violaine Ozenne, Marie-Pierre Galais, Claire Pérignon, Louise Lebedel, Marion Habireche, Apolline Commin, Thông Dao, Charlotte Costentin, Aurore Baron, Isabelle Ollivier Hourmand

Table of contents

Supplementary data 1…………………………………………………………………….2

Supplementary data 2…………………………………………………………………….3

Supplementary data 3…………………………………………………………………….4

Supplementary data 4…………………………………………………………………….5

Supplementary data 5…………………………………………………………………….6

Supplementary data 6…………………………………………………………………….7

Supplementary data 7…………………………………………………………………….8

Supplementary data 8…………………………………………………………………….9

Supplementary data 9…………………………………………………………………...10

Supplementary data 10………………………………………………………………….11

Supplementary data 11………………………………………………………………….12

Supplementary data 12………………………………………………………………….13

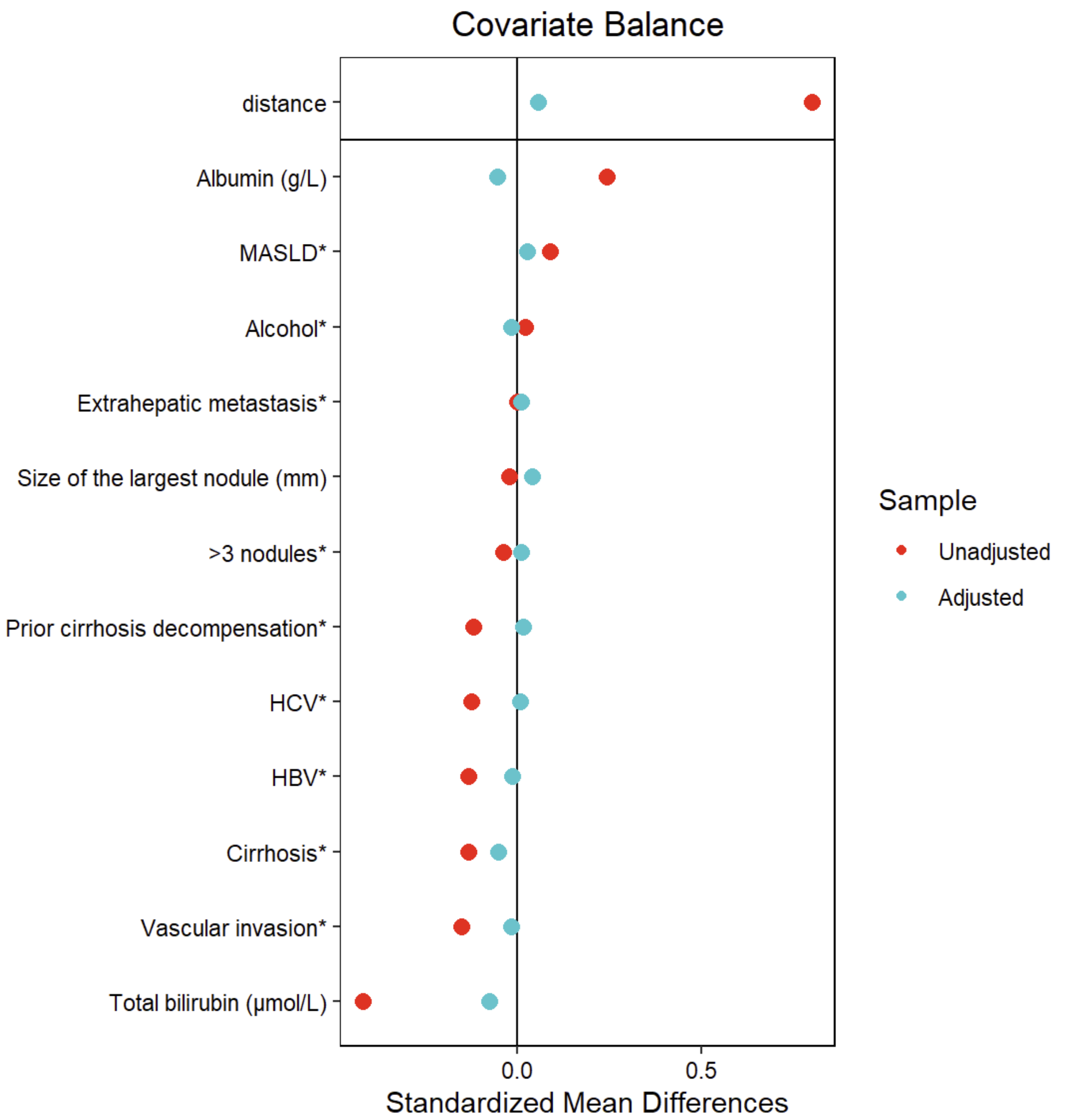

Supplementary data 1: Covariate balance before and after propensity score matching

Love plot showing standardized mean differences for baseline covariates before (red dots) and after (blue dots) matching. Each dot represents the difference in means (for continuous variables) or proportions (for categorical variables) between treatment groups. The vertical line at zero indicates perfect balance. After matching, all covariates demonstrated improved balance with standardized mean differences approaching zero. An asterisk (*) denotes categorical variables. HBV, hepatitis B virus; HCV, hepatitis C virus; MASLD, metabolic dysfunction–associated steatotic liver disease.

Supplementary data 2: Baseline characteristics of patients treated with atezolizumab-bevacizumab before propensity score matching according to the center (n=814)

Baseline characteristics		N	Overall N = 814	Avicenne N = 140	Beaujon N = 169	Sud Francilien N = 6	CHU Caen N = 105	Henri Mondor N = 109	Pitié Salpêtrière N = 125	Saint Antoine N = 64	Toulouse N = 96	p-value
	Elderly patient	814	248 (30%)	35 (25%)	46 (27%)	5 (83%)	60 (57%)	32 (29%)	35 (28%)	11 (17%)	24 (25%)	>0.999
	Gender (male)	814	698 (86%)	115 (82%)	149 (88%)	6 (100%)	87 (83%)	98 (90%)	105 (84%)	59 (92%)	79 (82%)	>0.999
	Obesity	810	172 (21%)	26 (19%)	30 (18%)	1 (17%)	33 (31%)	16 (15%)	25 (20%)	11 (17%)	30 (31%)	>0.999
	Type 2 diabetes	814	336 (41%)	51 (36%)	64 (38%)	5 (83%)	41 (39%)	52 (48%)	52 (42%)	28 (44%)	43 (45%)	>0.999
	Arterial hypertension	814	490 (60%)	86 (61%)	91 (54%)	6 (100%)	45 (65%)	66 (61%)	74 (59%)	38 (59%)	61 (64%)	>0.999
	Dyslipidemia	814	215 (26%)	36 (26%)	38 (22%)	5 (83%)	31 (30%)	38 (35%)	30 (24%)	13 (20%)	24 (25%)	>0.999
	Anticoagulation	794	136 (17%)	23 (16%)	27 (17%)	2 (33%)	15 (14%)	19 (19%)	27 (22%)	7 (11%)	16 (17%)	>0.999
	Cirrhosis	813	605 (74%)	100 (72%)	112 (66%)	5 (83%)	70 (67%)	83 (76%)	107 (86%)	53 (83%)	75 (78%)	>0.999
	ECOG 0-1<br>ECOG 2-3	700	644 (93%)<br>56 (7%)	125 (89%)<br>8 (11%)	141 (83%)<br>16 (17%)	4 (67%)<br>2 (3%)	100 (95%)<br>5 (5%)	65 (87%)<br>10 (13%)	117 (94%)<br>8 (6%)	3 (100%)<br>0 (0%)	89 (93%)<br>7 (7%)	>0.999
Etiologies of liver disease	At least ALD	814	326 (40%)	48 (34%)	49 (29%)	3 (50%)	42 (40%)	54 (50%)	54 (43%)	24 (38%)	52 (54%)	>0.999
	At least MASLD	814	339 (42%)	62 (44%)	57 (34%)	3 (50%)	39 (37%)	42 (39%)	62 (50%)	31 (48%)	43 (45%)	>0.999
	At least viral	814	324 (40%)	66 (47%)	66 (39%)	0 (0%)	27 (26%)	46 (42%)	55 (44%)	31 (48%)	33 (34%)	>0.999
	Mixed etiologies	814	366 (45%)	105 (75%)	36 (21%)	3 (50%)	20 (19%)	38 (35%)	98 (78%)	24 (38%)	42 (44%)	>0.999
Liver function	Previous cirrhosis decompensation	798	159 (20%)	21 (15%)	23 (14%)	1 (17%)	16 (15%)	17 (17%)	40 (32%)	13 (20%)	28 (29%)	>0.999
	Child-Pugh A	807	671 (83%)	97 (71%)	147 (89%)	5 (83%)	95 (90%)	87 (80%)	99 (79%)	50 (79%)	91 (95%)	>0.999
	Child-Pugh B		132 (16%)	39 (28%)	19 (11%)	1 (17%)	10 (10%)	22 (20%)	23 (18%)	13 (21%)	5 (5.2%)	
	Child-Pugh C		4 (0.5%)	1 (0.7%)	0 (0%)	0 (0%)	0 (0%)	0 (0%)	3 (2.4%)	0 (0%)	0 (0%)	
	mALBI grade 1	787	227 (29%)	34 (25%)	56 (33%)	4 (67%)	38 (36%)	23 (21%)	35 (28%)	14 (22%)	23 (30%)	>0.999
	mALBI grade 2a		182 (23%)	28 (20%)	31 (18%)	1 (17%)	25 (24%)	32 (30%)	30 (24%)	17 (27%)	18 (24%)	
	mALBI grade 2b		322 (41%)	62 (45%)	67 (40%)	0 (0%)	38 (36%)	48 (44%)	47 (38%)	29 (46%)	31 (41%)	
	mALBI grade 3		56 (7.1%)	13 (9.5%)	14 (8.3%)	1 (17%)	4 (3.8%)	5 (4.6%)	12 (9.7%)	3 (4.8%)	4 (5.3%)	
	No EV	770	416 (54%)	70 (53%)	71 (47%)	3 (50%)	72 (63%)	55 (56%)	62 (50%)	28 (47%)	55 (57%)	>0.999
	EV (regardless the size)		354 (46%)	62 (47%)	80 (53%)	3 (50%)	33 (37%)	43 (44%)	61 (50%)	31 (53%)	41 (43%)	>0.999
	Large size EV		177 (23%)	32 (25%)	45 (30%)	2 (33%)	8 (13%)	21 (12%)	30 (24%)	14 (23%)	22 (23%)	>0.999
	Creatinine (µmol/l)°	793	73.0 (62.0, 91.0)	72.0 (60.0, 96.5)	74.0 (62.0, 90.0)	81.0 (66.0, 123.0)	72.0 (59.3, 88.0)	76.0 (65.0, 92.0)	74.5 (64.0, 89.5)	72.0 (60.0, 91.0)	72.5 (61.0, 93.0)	0.646
	Total bilirubin (µmol/l)°	793	13.4 (9.0, 21.0)	15.0 (9.0, 21.5)	13.5 (9.0, 19.0)	13.8 (11.0, 19.0)	15.0 (10.0, 21.8)	13.8 (9.0, 23.7)	14.0 (9.0, 23.0)	13.0 (9.0, 23.0)	12.0 (9.5, 17.6)	0.723
	Albumin (g/L)°	791	36.0 (32.0, 39.0)	34.5 (31.0, 39.0)	35.2 (30.0, 40.0)	38.5 (37.0, 40.8)	37.0 (33.0, 40.0)	35.0 (32.0, 38.0)	36.0 (32.0, 39.0)	35.0 (31.0, 38.0)	36.5 (32.0, 39.0)	0.074
	INR°	756	1.1 (1.0, 1.2)	1.1 (1.1, 1.3)	1.1 (1.0, 1.2)	1.1 (1.0, 1.1)	1.1 (1.0, 1.3)	1.2 (1.1, 1.3)	1.1 (1.0, 1.3)	1.2 (1.1, 1.3)	1.0 (1.0, 1.1)	>0.999
HCC features	Previous HCC treatment	775	454 (59%)	77 (55%)	95 (56%)	1 (17%)	64 (93%)	54 (50%)	61 (49%)	44 (71%)	58 (60%)	>0.999
	BCLC-A	813	6 (0.7%)	1 (0.7%)	0 (0%)	0 (0%)	0 (0%)	3 (2.8%)	1 (0.8%)	0 (0%)	1 (1.0%)	>0.999
	BCLC-B		253 (31%)	44 (31%)	63 (37%)	0 (0%)	11 (10%)	32 (29%)	47 (38%)	28 (44%)	28 (29%)	
	BCLC-C		554 (68%)	95 (68%)	106 (63%)	5 (100%)	94 (90%)	74 (68%)	77 (62%)	36 (56%)	67 (70%)	
	AFP (ng/mL)°	786	76.0 (6.7, 1,763)	174.5 (13.0, 3,511)	115.0 (6.7, 2,940)	12.0 (4.4, 458.8)	25.7 (5.6, 251.5)	79.0 (9.1, 1,348.0)	118.0 (7.0, 1,804.0)	43.0 (6.0, 950.0)	12.8 (4.0, 975.0)	0.026
	AFP > 20 ng/mL	786	480 (61%)	98 (71%)	112 (66%)	2 (33%)	52 (50%)	70 (64%)	75 (60%)	33 (53%)	38 (45%)	>0.999
	AFP > 400 ng/mL	786	280 (36%)	57 (41%)	72 (43%)	2 (33%)	23 (22%)	36 (33%)	48 (38%)	18 (29%)	24 (29%)	>0.999
	> 3 lesions	800	443 (55%)	86 (62%)	87 (53%)	4 (67%)	49 (47%)	53 (51%)	58 (48%)	53 (83%)	53 (55%)	>0.999
	Tumor size > 5cm	743	399 (54%)	80 (60%)	92 (60%)	5 (100%)	32 (38%)	57 (56%)	68 (57%)	28 (47%)	37 (46%)	>0.999
	Extrahepatic lesions	814	249 (31%)	33 (24%)	50 (30%)	1 (17%)	40 (38%)	37 (34%)	32 (26%)	18 (28%)	38 (40%)	>0.999
	Vascular invasion	814	301 (37%)	54 (39%)	71 (42%)	4 (67%)	17 (16%)	46 (42%)	49 (39%)	20 (31%)	40 (42%)	>0.999

°median (IQR)

ALD : alcoholic-liver disease; AFP: alpha-fetoprotein; BCLC: Barcelona Clinic Liver Cancer; EV: esophageal varices; HCC : hepatocellular carcinoma ; INR: international normalized ratio; mALBI grade : modified Albumin-Bilirubin grade; MASLD : metabolic dysfunction-associated steatotic liver disease

Supplementary data 3: Flow chart of patients' selection

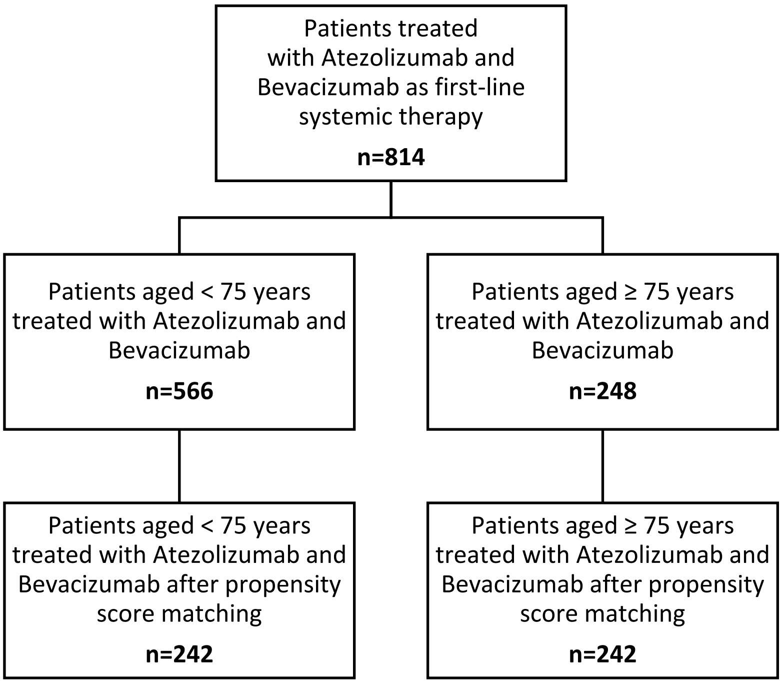

Supplementary data 4: Baseline characteristics of patients treated with atezolizumab-bevacizumab before propensity score matching (n=814)

Baseline characteristics		Available data	Whole cohort	Available data	Elderly patients n=248	Available data	Non-elderly patients n=566	p-value
	Gender (male)	814	698 (86%)	248	214 (86%)	566	484 (86%)	0.770
	Body mass index (Kg/m^2) °	809	25.8 (22.8-29.4)	244	26.1 (23.8,29.1)	565	25.6 (22.4,29.8)	0.228
	Obesity	810	172 (21%)	244	44 (18%)	566	128 (23%)	0.133
	Type 2 diabetes	814	336 (411%)	248	117 (48%)	566	104 (43%)	0.236
	Arterial hypertension	814	490 (605)	248	185 (75%)	566	305 (54%)	**<0.001**
	Dyslipidemia	814	215 (26%)	248	90 (36%)	566	125 (22%)	**<0.001**
	Anticoagulation	794	136 (17%)	242	55 (23%)	552	81 (15%)	**0.006**
	Cirrhosis	813	605 (75%)	248	162 (65%)	566	443 (78%)	**<0.001**
	ECOG 0-1	700	644 (92%)	229	203 (89%)	471	26 (11%)	**0.023**
	ECOG 2-3		56 (8.0%)		26 (11%)		30 (6.4%)	
**Etiologies of liver disease**	At least ALD	813	96 (125)	248	110 (44%)	566	216 (38%)	0.097
	At least MASLD	814	110 (14%)	248	111 (45%)	566	228 (40%)	0.233
	At least viral	813	148 (188)	248	54 (22%)	566	270 (48%)	**<0.001**
	Mixed etiologies	814	366 (45%)	248	99 (40%)	566	267 (47)	0.056
**Liver function Biology**	Previous cirrhosis decompensation	798	159 (20%)	242	28 (12%)	556	131 (24%)	**<0.001**
	MELD score	775	8 (7.0-11.0)	237	8 (7,10)	538	8 (7,11)	0.641
	Child-Pugh A	807	671 (83%)	247	220 (90%)	560	451 (80%)	**0.002**
	Child-Pugh B		132 (16%)		25 (10%)		107 (19%)	
	Child-Pugh C		4 (0.5%)		0 (0%)		4 (1%)	
	mALBI grade 1	787	227 (29%)	239	70 (29%)	548	157 (29%)	**<0.001**
	mALBI grade 2a		182 (23%)		76 (32%)		106 (19%)	
	mALBI grade 2b		322 (41%)		85 (36%)		237 (43%)	
	mALBI grade 3		56 (7.1%)		8 (3.3%)		48 (8.8%)	
	No EV	770	416 (54%)	231	141 (61%)	539	275 (51%)	0.033
	EV (regardless the size)	770	354 (46%)		90 (39%)		264 (49%)	
	Large size EV	770	177 (22.7%)		42 (18%)		135 (25%)	0.317
	Platelet count (x10^3/mm^3)°	795	171 (117, 250)	246	179 (125, 250)	549	166 (115,255)	0.283
	Creatinine (µmol/l)°	793	73 (62-91)	241	81 (66, 103)	552	71 (60,86)	**<0.001**
	Total bilirubin (µmol/l)°	793	13.4 (9-21)	241	12 (9, 18)	552	14 (10, 22)	**<0.001**
	Albumin (g/L)°	791	36 (32-39)	239	36 (33, 39)	552	35 (31, 39)	**0.012**
	INR°	756	1.1 (1.0-1.2)	233	1.1 (1.0, 1.2)	523	1.1 (1.0, 1.3)	**<0.001**
**HCC features**	Previous HCC treatment	775	454 (59%)	226	148 (65%)	549	306 (56%)	**0.012**
	BCLC-A	813	6 (0.7%)	247	1(1%)	566	5(1%)	0.552
	BCLC-B		253 (31%)		83 (33%)		170 (30%)	
	BCLC-C		554 (68%)		163 (66%)		391 (69%)	
	AFP (ng/mL)°	786	76 (6.7-1763)	235	54 (5,919)	551	95 (9,2500)	**0.010**
	AFP>20 ng/mL	786	480 (61%)	235	163 (69%)	551	384 (70%)	0.933
	AFP>400 ng/mL	786	333 (42%)	235	85 (86%)	551	209 (38%)	0.687
	>3 lesions	800	443 (55%)	247	132 (53%)	553	311 (56%)	0.462
	Size of the largest lesion (mm)°	743	52 (26-90)	229	51 (24, 80)	514	54 (28,100)	0.172
	Tumor size > 5cm	743	399 (54%)	229	120 (52%)	514	279 (54%)	0.635
	Extrahepatic lesions	814	249 (31%)	248	76 (31%)	566	173 (31%)	0.982
	Vascular invasion	814	301 (37%)	248	66 (27%)	566	235 (42%)	**<0.001**
**AtezoBev treatment**	Treatment duration (months)°	814	4.7 (1.9-11.3)	248	4.9 (1.5,11.7)	566	4.5 (2.0,10.9)	0.927
	Second-line treatment	695	229 (33%)	210	65 (31%)	485	164 (31%)	0.427

°median (IQR)

ALD : alcoholic-liver disease; AtezoBev : Atezolizumab-Bevacizumab; AFP: alpha-fetoprotein; BCLC: Barcelona Clinic Liver Cancer; EV: esophageal varices; HCC : hepatocellular carcinoma ; INR: international normalized ratio; mALBI grade : modified Albumin-Bilirubin grade; MASLD : metabolic dysfunction-associated steatotic liver disease; MELD score : Model for End-Stage Liver Disease

Supplementary data 5: Overall Survival and Progression-Free Survival in the whole cohort of patients (n=814)

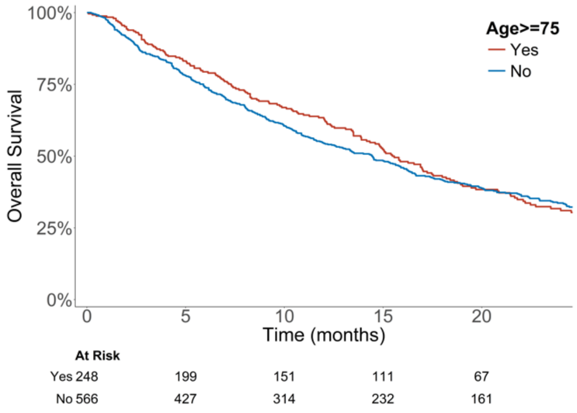

P=0.486

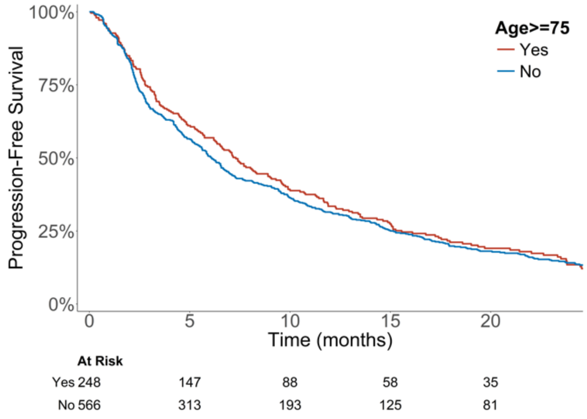

P=0.372

Supplementary data 6: Baseline predictive factors of progression free survival in the propensity-score matched population regardless of age (n=484) (Cox proportional hazard regression models)

	Available data (n=484)	Univariate analysis			Multivariate analysis		
		HR	95 CI	p value	HR	95 CI	p value
Age ≥ 75 years		0.96	0.79, 1.17	0.706			
Gender (male)	484	0.77	0.59, 1.01	**0.050**	0.81	0.60,1.09	0.163
Obesity	481	0.86	0.67, 1.09	0.209			
Type 2 diabetes	484	0.83	0.68, 1.01	0.060			
Arterial hypertension	484	0.82	0.67, 1.01	0.061			
Dyslipidemia	484	1.04	0.85, 1.28	0.677			
Anticoagulation	470	0.91	0.70, 1.18	0.490			
Cirrhosis	483	0.92	0.74, 1.13	0.431			
ECOG 0-1<br>ECOG 2-3	424	-<br>1.68	-<br>1.19, 2.37	-<br>**0.003**	-<br>1.42	-<br>0.97-2.08	-<br>0.068
At least ALD	484	0.99	0.82, 1.21	0.936			
At least MASLD	484	0.89	0.74, 1.09	0.263			
At least viral	484	1.07	0.86, 1.34	0.527			
Mixed etiologies	484	0.97	0.80, 1.19	0.795			
Esophageal varices	456	1.09	0.89, 1.34	0.385			
Large size EV	456	1.04	0.93, 1.16	0.514			
Previous cirrhosis decompensation	473	1.36	1.00, 1.84	**0.048**	1.13	0.80, 1.62	0.486
Platelet count	471	1.00	1.00, 1.00	**0.018**			
Albumin°	469	0.95	0.93, 0.97	**<0.001**			
Total bilirubin°	469	1.01	1.01, 1.02	**0.001**			
Creatinine	469	1.00	1.00, 1.00	0.430			
INR	449	1.34	0.80, 2.25	0.267			
MELD Score°	458	1.01	0.98, 1.05	0.360			
Child-Pugh C°<br>Child-Pugh B<br>Child-Pugh A	479	-<br>1.57<br>0.62	-<br>1.08, 2.27<br>0.43, 0.89	-<br>**0.017**<br>**0.011**			
mALBI grade 1<br>mALBI grade2a<br>mALBI grade2b<br>mALBI grade3	465	-<br>1.20<br>1.63<br>3.74	-<br>0.92, 1.56<br>1.29, 2.07<br>2.21, 6.34	-<br>0.182<br>**<0.001**<br>**<0.001**	-<br>1.27<br>1.59<br>3.77	-<br>0.94, 1.72<br>1.22, 2.06<br>2.12,6.70	**-**<br>0.123<br>**<0.001**<br>**<0.001**
Previous HCC treatment	455	1.07	0.85, 1.35	0.571			
BCLC-A<br>BCLC-B<br>BCLC-C	483	-<br>0.53<br>0.61	-<br>0.21, 1.28<br>0.25, 1.48	-<br>0.158<br>0.275			
AFP (ng/mL)	463	1.00	1.00, 1.00	**0.012**			
AFP >20 (ng/mL)	463	1.46	1.19, 1.79	**<0.001**			
AFP > 400 ng/mL	463	1.50	1.22, 1.86	**<0.001**	1.48	1.16, 1.88	**0.002**
>3 lesions	479	1.03	0.85, 1.26	0.741			
Size of the largest lesion (mm)	437	1.00	1.00, 1.00	0.746			
Tumor size > 5cm	437	1.04	0.85, 1.28	0.678			
Extrahepatic lesions	484	1.24	1.00, 1.53	**0.046**	1.48	1.17, 1.86	**0.001**
Vascular invasion	484	0.95	0.77, 1.19	0.675			

* ECOG performance status was not included in the multivariate analysis because the proportion of missing values exceeded the predefined 10% threshold for imputation.

° Child-Pugh score, MELD score, albumin and bilirubin were not entered in the multivariate analysis in order to avoid collinearity with ALBI grade 3 classification.

ALD : alcoholic-liver disease; AtezoBev : Atezolizumab-Bevacizumab; AFP: alpha-fetoprotein; BCLC: Barcelona Clinic Liver Cancer; EV: esophageal varices; HCC : hepatocellular carcinoma ; INR: international normalized ratio; mALBI grade : modified Albumin-Bilirubin grade; MASLD : metabolic dysfunction-associated steatotic liver disease; MELD score : Model for End-Stage Liver Disease

Supplementary data 7: Baseline predictive factors of mortality in propensity-score matched population regardless of age (n=484) (Cox proportional hazard regression models)

	Available data (n=484)	Univariate analysis			Multivariate analysis		
		**HR**	**95 CI**	**p value**	**HR**	**95 CI**	**p value**
Age ≥ 75 years	484	0.99	0.80-1.23	0.937			
Gender (male)	484	1.00	0.73, 1.35	0.980			
Obesity	481	0.86	0.65, 1.13	0.274			
Type 2 diabetes	484	0.90	0.73, 1.12	0.362			
Arterial hypertension	484	0.95	0.76, 1.19	0.655			
Dyslipidemia	484	1.23	0.97, 1.54	0.083			
Anticoagulation	470	1.01	0.75, 1.35	0.973			
Cirrhosis	483	0.98	0.78, 1.24	0.890			
ECOG 0-1<br>ECOG 2-3	424	-<br>1.89	-<br>1.30, 2.75	**-**<br>**<0.001**	-<br>1.56	-<br>1.01-2.42	-<br>**0.046**
At least ALD	484	1.30	0.96, 1.77	0.095			
At least MASLD	484	1.01	0.76, 1.33	0.954			
At least viral	484	0.81	0.57, 1.16	0.250			
Mixed etiologies	484	1.00	0.80, 1.25	0.983			
Esophageal varices	456	1.26	1.00, 1.58	**0.045**	1.02	0.77, 1.36	0.867
Large size EV	456	1.13	1.00, 1.28	0.051			
Previous cirrhosis decompensation	473	1.33	0.92, 1.92	0.126			
Platelet count	471	1.00	1.00, 1.00	0.073			
Albumin°	469	0.94	0.92, 0.96	**<0.001**			
Total bilirubin°	469	1.02	1.01, 1.03	**<0.001**			
Creatinine	469	1.00	1.00, 1.00	0.953			
INR	449	1.64	0.99-2.74	0.056			
MELD Score°	458	1.04	1.01, 1.07	**0.014**			
Child-Pugh C°<br>Child-Pugh B<br>Child-Pugh A	479	-<br>2.02<br>0.48	-<br>1.44, 2.85<br>0.34, 0.67	-<br>**<0.001**<br>**<0.001**			
mALBI grade 1<br>mALBI grade2a<br>mALBI grade2b<br>mALBI grade3	465	-<br>1.46<br>1.99<br>5.10	-<br>1.08, 1.97<br>1.52, 2.61<br>2.88, 9.03	**-**<br>**0.014**<br>**<0.001**<br>**<0.001**	-<br>1.45<br>1.60<br>4.58	-<br>0.99, 2.12<br>1.13, 2.26<br>2.36, 8.88	-<br>0.057<br>**0.007**<br>**<0.001**
Previous HCC treatment	455	0.71	0.57, 0.90	**0.004**	0.98	0.71, 1.35	0.904
BCLC-A<br>BCLC-B<br>BCLC-C	483	-<br>1.15<br>1.54	-<br>0.36, 3.64<br>0.49, 4.83	-<br>0.812<br>0.459			
AFP (ng/mL)	463	1.00	1.00, 1.00	**<0.001**			
AFP> 20 ng/mL		1.46	1.20, 1.91	**0.001**			
AFP > 400 ng/mL	463	1.51	1.20, 1.93	**<0.001**	1.46	1.09, 1.96	**0.011**
>3 lesions	479	1.08	0.87, 1.34	0.500			
Size of the largest lesion (mm)	437	1.00	1.00, 1.00	**0.017**			
Tumor size > 5cm	437	1.37	1.09, 1.73	**0.007**	1.08	0.79, 1.48	0.636
Extrahepatic lesions	484	1.17	0.92, 1.48	0.201			
Vascular invasion	484	1.25	0.98, 1.58	0.069			

* ECOG performance status was not included in the multivariate analysis because the proportion of missing values exceeded the predefined 10% threshold for imputation.

° Child-Pugh score, MELD score, albumin and bilirubin were not entered in the multivariate analysis in order to avoid collinearity with ALBI grade 3 classification.

ALD : alcoholic-liver disease; AtezoBev : Atezolizumab-Bevacizumab; AFP: alpha-fetoprotein; BCLC: Barcelona Clinic Liver Cancer; EV: esophageal varices; HCC : hepatocellular carcinoma ; INR: international normalized ratio; mALBI grade : modified Albumin-Bilirubin grade; MASLD : metabolic dysfunction-associated steatotic liver disease; MELD score : Model for End-Stage Liver Disease

Supplementary data 8: Baseline characteristics of patients with an mALBI score of 3 treated with atezolizumab-bevacizumab (n=16)

Baseline characteristics		Available data	Non-elderly patients (< 75 years) n=8	Elderly patients (≥ 75 years) n=8	p-value
Age (years)		16	57.5 (53.7, 61.7)	78.5 (76.0, 80.5)	**<0.001**
Gender (male)		16	5 (63%)	6 (75%)	>0.999
Body mass index (Kg/m^2) °		16	23.9 (18.2, 24.8)	24.8 (23.5, 26.5)	0.328
Obesity		16	0 (0%)	0 (0%)	**0.328**
Type 2 diabetes		16	2 (25%)	5 (63%)	0.315
Arterial hypertension		16	2 (25%)	5 (63%)	0.315
Dyslipidemia		16	0 (0%)	5 (63%)	**0.026**
Anticoagulation		16	0 (0%)	1 (13%)	>0.999
Cirrhosis		16	5 (63%)	6 (75%)	>0.999
ECOG 0-1<br>ECOG 2-3		16	5 (63%)<br>3 (38%)	8 (100%)<br>0 (0%)	0.200
**Etiologies of liver disease**	At least ALD	16	1 (13%)	3 (38%)	0.569
	At least MASLD	16	3 (38%)	7 (88%)	0.119
	At least viral	16	2 (25%)	0 (0%)	0.467
	Mixed etiologies	16	3 (38%)	4 (50%)	>0.999
**Liver function**	Previous cirrhosis decompensation	16	3 (38%)	3 (38%)	>0.999
	Child-Pugh A	16	2 (25%)	1 (13%)	0.259
	Child-Pugh B		5 (63%)	7 (87%)	
	Child-Pugh C		3 (12%)	0 (0%)	
	No EV	16	4 (50%)	2 (25%)	0.608
	EV (regardless the size)		4 (50%)	6 (75%)	
	Large size EV		3 (37%)	2 (25%)	0.277
	Platelet count (x10^3/mm^3)°	16	188 (128, 277)	124 [(98, 265)	0.442
	Creatinine (µmol/l)°	16	60.0 (54.5, 72.0)	66.5 (51.9, 78.0)	0.721
	Total bilirubin (µmol/l)°	16	31.7 (15.0, 69.0)	31.1 (25.2, 37.7)	>0.999
	INR°	16	1.2 (1.1, 1.4)	1.4 (1.2, 1.5)	0.247
**HCC features**	Previous HCC treatment	14	4 (50%)	2 (33%)	0.627
	BCLC-B	16	4 (50%)	1 (13%)	0.282
	BCLC-C		4 (50%)	7 (88%)	
	AFP (ng/mL)°	16	414.0 (5.6, 13,897.5)	63.5([4.5, 1,014.4)	0.574
	AFP > 20 ng/mL	16	5 (63%)	4 (50%)	>0.999
	AFP > 400 ng/mL	16	4 (50%)	3 (38%)	>0.999
	> 3 lesions	16	4 (50%)	4 (50%)	>0.999
	Size of the largest lesion (mm)°	16	100.0 (80.0, 105.0)	59.5 (33.5, 110.0)	0.114
	Tumor size > 5cm	16	8 (100%)	5 (63%)	0.200
	Extrahepatic lesions	16	2 (25%)	1 (13%)	>0.999
	Vascular invasion	16	2 (25%)	3 (38%)	>0.999
**AtezoBev treatment**	Treatment duration (months)°	16	1.4 (0.7, 3.7)	1.4 (0.9, 2.2)	>0.999
	Second-line treatment	16	4 (50%)	2 (25%)	0.608

°median (IQR)

ALD : alcoholic-liver disease; AtezoBev : Atezolizumab-Bevacizumab; AFP: alpha-fetoprotein; BCLC: Barcelona Clinic Liver Cancer; EV: esophageal varices; HCC : hepatocellular carcinoma ; INR: international normalized ratio; mALBI grade : modified Albumin-Bilirubin grade; MASLD : metabolic dysfunction-associated steatotic liver disease

Supplementary data 9: Comparison between patients according to the occurrence of hypertension during treatment

Baseline characteristics		Elderly patients n=223		p-value
		**Yes n=39**	**No n=184**	
Gender (male)		32 (82%)	160 (87%)	0.421
Body mass index (Kg/m^2) °		27 (24, 30)	26 (24, 29)	0.129
Obesity		10 (26%)	29 (16%)	0.154
Type 2 diabetes		23 (59%)	85 (46%)	0.147
Arterial hypertension		31 (79%)	133 (72%)	0.354
Dyslipidemia		18 (46%)	64 (35%)	0.181
Anticoagulation		8 (21%)	40 (22%)	0.853
Cirrhosis		25 (64%)	120 (65%)	0.895
**Performance Status (ECOG)**	0	18 (46%)	59 (33%)	0.452
	1	19 (49%)	97 (54%)	
	2	2 (5%)	18 (10%)	
	3	0 (0%)	4 (2%)	
**Etiologies of liver disease**	At least ALD	15 (38%)	78 (42%)	0.651
	At least MASLD	21 (54%)	80 (43%)	0.237
	At least viral	9 (23%)	41 (22%)	0.914
	Mixed etiologies	13 (33%)	75 (41%)	0.389
**History of decompensated cirrhosis**	Previous cirrhosis decompensation	6 (15%)	17 (9.3%)	0.254
	Previous hepatic encephalopathy	1 (2.6%)	10 (5.4%)	0.694
	Previous variceal bleeding	0 (0%)	6 (3.3%)	0.593
	Previous ascitic decompensation	5 (13%)	13 (7.1%)	0.327
	Previous ascitic infection	1 (2.6%)	3 (1.6%)	0.539
	Beta-blocker therapy	7 (18%)	50 (27%)	0.230
**Liver function**	Child-Pugh A	38 (97%)	159 (88%)	0.087
	Child-Pugh B	1 (2.6%)	22 (12%)	0.087
	mALBI grade 1	19 (53%)	62 (35%)	**0.043**
	mALBI grade 2-3	17 (47%)	116 (65%)	
	MELD score	8 (7, 11)	8 (7, 10)	0.844
	No EV	27 (68%)	117 (61%)	0.428
	EV (regardless the size)	12 (32%)	67 (39%)	
	Large size EV	6 (16%)	31 (18%)	0.762
	Platelet count (x10^3/mm^3)°	176 (121, 210)	185 (130, 272)	0.098
**Biology**	Creatinine (µmol/l)°	81 (64, 96)	83 (67, 103)	0.318
	Total bilirubin (µmol/l)°	12 (10, 16)	12 (9, 19)	0.951
	Albumin (g/L)°	38 (36, 41)	36 (33, 39)	**0.009**
	INR°	1.1 (1.0, 1.2)	1.1 (1.0, 1.2)	0.316
**HCC features**	Active monitoring of cirrhosis	12 (31%)	60 (33%)	0.807
	Previous HCC treatment	24 (73%)	106 (63%)	0.290
	BCLC-A	0 (0%)	1 (0.5%)	0.640
	BCLC-B	10 (26%)	60 (33%)	
	BCLC-C	28 (74%)	123 (67%)	
	AFP (ng/mL)°	30 (5,875)	59 (5,1065)	0.535
	Monofocal	5 (13%)	55 (30%)	**0.028**
	Size of the largest lesion (mm)°	56 (29,88)	50 (23,80)	0.421
	Tumor size > 5cm	20 (59%)	88 (52%)	0.452
	Extrahepatic lesions	13 (33%)	60 (33%)	0.930
	Vascular invasion	7 (18%)	51 (28%)	0.207
**AtezoBev treatment**	Treatment duration (months)°	8.3 (2.8,14.8)	4.1 (1.4,9.7)	**0.009**
	Second-line treatment	11 (31%)	51 (31%)	0.917

°median (IQR)

ALD : alcoholic-liver disease; AtezoBev : Atezolizumab-Bevacizumab; AFP: alpha-fetoprotein; BCLC: Barcelona Clinic Liver Cancer; EV: esophageal varices; HCC : hepatocellular carcinoma ; INR: international normalized ratio; mALBI grade : modified Albumin-Bilirubin grade; MASLD : metabolic dysfunction-associated steatotic liver disease; MELD score : Model for End-Stage Liver Disease

Supplementary data 10: Hypertension occurrence according to hypertension (HTN) prior history. This alluvial plot illustrates the occurrence of arterial hypertension during treatment, defined according to CTCAE criteria, stratified by hypertension status at baseline. The left side of the plot represents the presence or absence of hypertension at baseline, while the right side represents the development of on-treatment arterial hypertension.

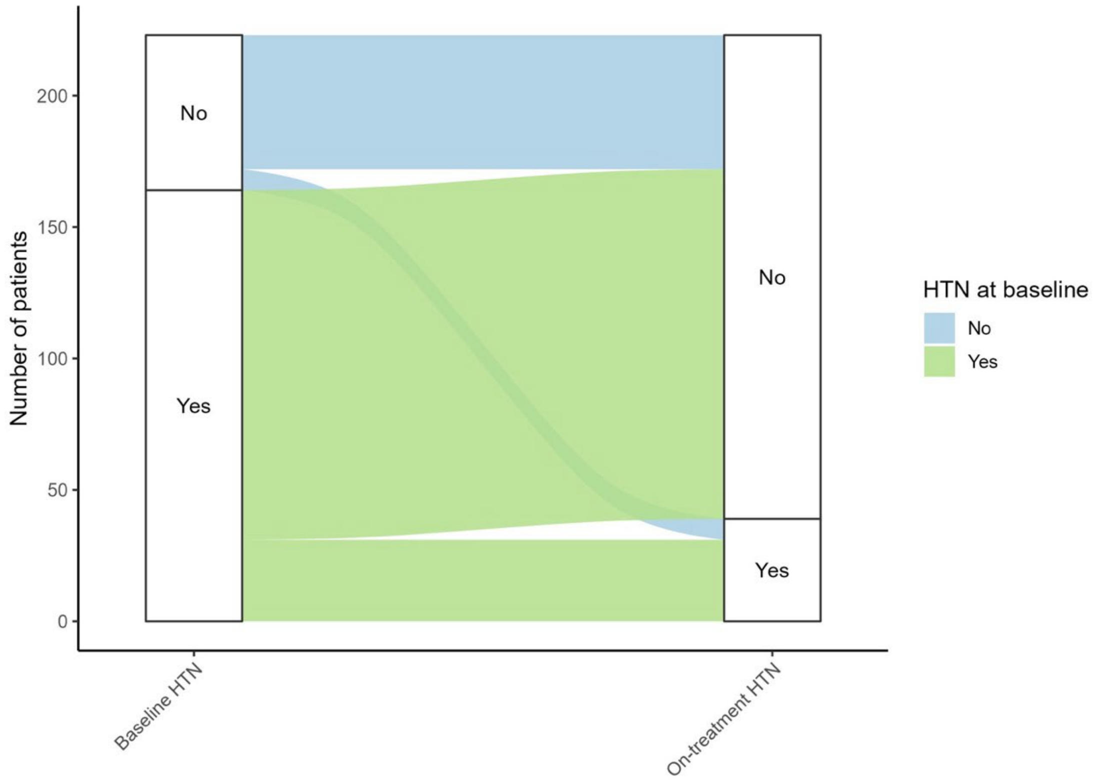

Supplementary data 11. Comparison between patients according to the occurrence of proteinuria during treatment

Baseline characteristics		Elderly patients n=223		p-value
		Yes n=20	No n=203	
Gender (male)		16 (80%)	176 (87%)	0.494
Body mass index (Kg/m^2) °		26 (24, 29)	26 (24, 29)	0.902
Obesity		4 (20%)	35 (18%)	0.761
Type 2 diabetes		13 (65%)	95 (47%)	0.120
Arterial hypertension		17 (85%)	147 (72%)	0.223
Dyslipidemia		4 (20%)	78 (38%)	0.103
Anticoagulation		2 (10%)	46 (23%)	0.259
Cirrhosis		12 (60%)	133 (66%)	0.622
**Performance Status (ECOG)**	0	9 (45%)	68 (35%)	0.756
	1	9 (45%)	107 (54%)	
	2	2 (10%)	18 (9.1%)	
	3	0 (0%)	4 (2.0%)	
**Etiologies of liver disease**	At least ALD	9 (45%)	84 (41%)	0.754
	At least MASLD	11 (55%)	90 (44%)	0.361
	At least viral	7 (35%)	43 (21%)	0.166
	Mixed etiologies	8 (40%)	80 (39%)	0.959
**History of decompensated cirrhosis**	Previous cirrhosis decompensation	1 (5.0%)	22 (11%)	0.702
	Previous hepatic encephalopathy	0 (0%)	11 (5.4%)	0.605
	Previous variceal bleeding	0 (0%)	6 (3.0%)	>0.999
	Previous ascitic decompensation	0 (0%)	18 (8.9%)	0.381
	Previous ascitic infection	1 (5.0%)	3 (1.5%)	0.315
	Beta-blocker therapy	2 (10%)	55 (27%)	0.095
**Liver function**	Child-Pugh A	20 (100%)	177 (89%)	0.239
	Child-Pugh B	0 (0%)	23 (12%)	0.239
	mALBI grade 1	11 (55%)	70 (36%)	0.097
	mALBI grade 2-3	9 (45%)	124 (64%)	
	MELD score	8 (6, 10)	8 (7, 10)	0.213
	No EV	15 (72%)	129 (61%)	0.342
	EV (regardless the size)	5 (28%)	74 (39%)	
	Large size EV	0 (0%)	37 (20%)	0.188
	Platelet count (x10^3/mm^3)°	183 (147, 246)	181 (126, 252)	0.763
**Biology**	Creatinine (µmol/l)°	74 (62, 94)	83 (67, 103)	0.342
	Total bilirubin (µmol/l)°	10 (7, 15)	12 (9, 19)	0.103
	Albumin (g/L)°	37 (35, 42)	36 (33, 39)	**0.043**
	INR°	1.1 (1.0, 1.2)	1.1 (1.0, 1.2)	0.316
**HCC features**	Active monitoring of cirrhosis	7 (35%)	65 (32%)	0.797
	Previous HCC treatment	12 (67%)	118 (64%)	0.853
	BCLC-A	0 (0%)	1 (0.5%)	0.378
	BCLC-B	4 (20%)	66 (33%)	
	BCLC-C	16 (80%)	135 (67%)	
	AFP (ng/mL)°	66 (5,394)	58 (5,973)	0.642
	Monofocal	3 (15%)	57 (28%)	0.204
	Size of the largest lesion (mm)°	46 (26,83)	51 (26,83)	0.515
	Tumor size > 5cm	8 (50%)	100 (53%)	0.806
	Extrahepatic lesions	5 (25%)	68 (33%)	0.440
	Vascular invasion	6 (30%)	52 (26%)	0.670
**AtezoBev treatment**	Treatment duration (months)°	13 (5.1,15.2)	4.2 (1.4,10.3)	**0.009**
	Second-line treatment	7 (37%)	55 (30%)	0.541

°median (IQR)

ALD : alcoholic-liver disease; AtezoBev : Atezolizumab-Bevacizumab; AFP: alpha-fetoprotein; BCLC: Barcelona Clinic Liver Cancer; EV: esophageal varices; HCC : hepatocellular carcinoma ; INR: international normalized ratio; mALBI grade : modified Albumin-Bilirubin grade; MASLD : metabolic dysfunction-associated steatotic liver disease; MELD score : Model for End-Stage Liver Disease

Supplementary data 12: Comparison between patients according to the occurrence of immune-related adverse events during treatment

Baseline characteristics		Elderly patients n=226		p-value
		Yes n=50	No n=176	
Gender (male)		48 (96%)	145 (82%)	**0.016**
Body mass index (Kg/m²) °		26 (24, 29)	26 (24, 29)	0.660
Obesity		9 (18%)	31 (18%)	0.990
Type 2 diabetes		22 (44%)	86 (49%)	0.543
Arterial hypertension		40 (80%)	126 (72%)	0.235
Dyslipidemia		19 (38%)	63 (36%)	0.775
Anticoagulation		15 (30%)	35 (20%)	0.134
Cirrhosis		37 (74%)	110 (63%)	0.132
**Performance Status (ECOG)**	0	20 (41%)	59 (35%)	0.199
	1	27 (55%)	90 (53%)	
	2	1 (2.0%)	19 (11%)	
	3	1 (2.0%)	3 (1.8%)	
**Etiologies of liver disease**	At least ALD	21 (42%)	73 (41%)	0.947
	At least MASLD	18 (36%)	84 (48%)	0.141
	At least viral	16 (32%)	35 (20%)	0.071
	Mixed etiologies	17 (34%)	73 (41%)	0.341
**History of decompensated cirrhosis**	Previous cirrhosis decompensation	17 (9.3%)	6 (15%)	0.254
	Previous hepatic encephalopathy	3 (6.0%)	8 (4.5%)	0.711
	Previous variceal bleeding	2 (4.0%)	4 (2.3%)	0.616
	Previous ascitic decompensation	3 (6.0%)	16 (9.1%)	0.773
	Previous ascitic infection	1 (2.0%)	3 (1.7%)	>0.999
	Beta-blocker therapy	12 (24%)	46 (26%)	0.760
**Liver function**	Child-Pugh A	45 (94%)	156 (89%)	0.424
	Child-Pugh B	3 (6.3%)	19 (11%)	0.424
	mALBI grade 1	26 (55%)	57 (34%)	**0.007**
	mALBI grade 2-3	21 (45%)	113 (66%)	
	MELD score	8 (7, 12)	8 (7, 10)	0.440
	No EV	32 (62%)	112 (61%)	0.905
	EV (regardless the size)	18 (38%)	64 (39%)	
	Large size EV	5 (15%)	30 (19%)	0.545
	Platelet count (x10³/mm³)°	168 (137, 222)	188 (126, 267)	0.470
**Biology**	Creatinine (µmol/l)°	85 (66, 99)	80 (66, 103)	0.587
	Total bilirubin (µmol/l)°	14 (8, 18)	12 (9, 18)	0.476
	Albumin (g/L)°	38 (36, 41)	36 (32, 39)	**0.002**
	INR°	1.1 (1.0, 1.2)	1.1 (1.0, 1.2)	0.325
**HCC features**	Active monitoring of cirrhosis	15 (30%)	59 (34%)	0.622
	Previous HCC treatment	32 (65%)	100 (65%)	0.920
	BCLC-A	1 (2.0%)	0 (0%)	0.079
	BCLC-B	19 (38%)	51 (29%)	
	BCLC-C	30 (60%)	124 (71%)	
	AFP (ng/mL)°	37 (5,430)	65 (5,1079)	0.429
	Monofocal	14 (29%)	47 (27%)	0.795
	Size of the largest lesion (mm)°	45 (22,89)	52 (27,80)	0.685
	Tumor size > 5cm	21 (50%)	88 (53%)	0.699
	Extrahepatic lesions	12 (24%)	61 (35%)	0.155
	Vascular invasion	15 (30%)	46 (26%)	0.587
**AtezoBev treatment**	Treatment duration (months)°	10.6 (2.8,19.0)	4.1 (1.5,8.9)	**<0.001**
	Second-line treatment	9 (20%)	53 (33%)	0.090

°median (IQR)

ALD : alcoholic-liver disease; AtezoBev : Atezolizumab-Bevacizumab; AFP: alpha-fetoprotein; BCLC: Barcelona Clinic Liver Cancer; EV: esophageal varices; HCC : hepatocellular carcinoma ; INR: international normalized ratio; mALBI grade : modified Albumin-Bilirubin grade; MASLD : metabolic dysfunction-associated steatotic liver disease; MELD score : Model for End-Stage Liver Disease
